# Plasma metabolomics of oral squamous cell carcinomas based on NMR and MS approaches provides biomarker identification and survival prediction

**DOI:** 10.1038/s41598-023-34808-2

**Published:** 2023-05-26

**Authors:** Giovana Mussi Polachini, Tialfi Bergamin de Castro, Luis Fabiano Soares Smarra, Tiago Henrique, Carlos Henrique Diniz de Paula, Patricia Severino, Rossana Veronica Mendoza López, André Lopes Carvalho, Ana Carolina de Mattos Zeri, Ismael Dale Cotrim Guerreiro Silva, Eloiza H. Tajara

**Affiliations:** 1 Department of Molecular Biology, School of Medicine of São José Do Rio Preto - FAMERP, Av. Brigadeiro Faria Lima, 5416, Vila São Pedro, São José do Rio Preto, SP CEP 15090-000 Brazil; 2 Department of Genetics and Evolutionary Biology, Institute of Biosciences, University of São Paulo, São Paulo, SP Brazil; 3 Albert Einstein Research and Education Institute, Hospital Israelita Albert Einstein, São Paulo, SP Brazil; 4 Center for Translational Research in Oncology, Institute of Cancer of São Paulo State, São Paulo, SP Brazil; 5 Molecular Oncology Research Center, Barretos Cancer Hospital, Barretos, SP Brazil; 6 Ilum School of Science, Brazilian Center for Research in Energy and Materials – CNPEM, Campinas, SP Brazil; 7 Department of Gynecology, Federal University of São Paulo, São Paulo, SP Brazil; 8 Fleury Laboratories, São Paulo, SP Brazil

**Keywords:** Cancer, Molecular biology, Biomarkers

## Abstract

Metabolomics has proven to be an important omics approach to understand the molecular pathways underlying the tumour phenotype and to identify new clinically useful markers. The literature on cancer has illustrated the potential of this approach as a diagnostic and prognostic tool. The present study aimed to analyse the plasma metabolic profile of patients with oral squamous cell carcinoma (OSCC) and controls and to compare patients with metastatic and primary tumours at different stages and subsites using nuclear magnetic resonance and mass spectrometry. To our knowledge, this is the only report that compared patients at different stages and subsites and replicates collected in diverse institutions at different times using these methodologies. Our results showed a plasma metabolic OSCC profile suggestive of abnormal ketogenesis, lipogenesis and energy metabolism, which is already present in early phases but is more evident in advanced stages of the disease. Reduced levels of several metabolites were also associated with an unfavorable prognosis. The observed metabolomic alterations may contribute to inflammation, immune response inhibition and tumour growth, and may be explained by four nonexclusive views—differential synthesis, uptake, release, and degradation of metabolites. The interpretation that assimilates these views is the cross talk between neoplastic and normal cells in the tumour microenvironment or in more distant anatomical sites, connected by biofluids, signalling molecules and vesicles. Additional population samples to evaluate the details of these molecular processes may lead to the discovery of new biomarkers and novel strategies for OSCC prevention and treatment.

## Introduction

In the past 2 decades, we have seen tremendous growth in the knowledge on the human genome and transcriptome, which has provided hundreds of opportunities to develop new treatments for human diseases, although not all have yielded the success that was initially hoped for. Many data were generated and important questions were answered regarding gene structure and expression, revealing unexpected transcription controls and intricate signalling networks^1,2^. Although proteins are usually viewed as cellular end effectors, metabolites have emerged as the true end-point mediators able to interact and liaise with other molecules to generate responses to normal and abnormal stimuli, thus participating in gene expression and enzymatic activity regulation and affecting function and phenotype^3^.

The set of thousands of metabolites in a biological system, defined as the metabolome, changes continually in quality and relative concentrations in tissues and fluids, and these variations are dependent on several parameters, such as sex, age, nutritional and lifestyle factors, and healthy and disease states. Considering the metabolites present in the whole human body, the last version of the Human Metabolome Database/HMDB (https://hmdb.ca/; 5) includes 220,945 metabolite entries in addition to pathways and associated diseases. The HMDB also provides information on more than 35,000 metabolites that have already been detected or are expected to be present in the blood.

Several methodologies, either targeted or nontargeted, have been used to assess specific or as many metabolites as possible, respectively, such as nuclear magnetic resonance (NMR) and mass spectrometry (MS)^1^. NMR spectroscopy is a highly reproducible quantitative tool for nontargeted metabolic profiling that performs several analyses in samples that include biofluids and tissue extracts. It allows us to look at the atomic level of molecules and to evaluate particular metabolites (such as ions and protein-bound or inorganic metabolites), which cannot be performed by MS^1^. Unfortunately, it has low sensitivity and detects few metabolites. In addition, for example in plasma, protein bound to small molecules may affect the quantitative NMR analysis and the visibility of several relevant compounds^2^. In turn, MS shows a higher sensitivity and, depending on the platform, is able to measure the concentration of almost 80% of the serum metabolites. Although each methodology has limitations, combining or modifying platforms maximizes metabolite detection^3,4^. This is the case for high-resolution magic angle spinning (HR–MAS) MR spectroscopy^5^, gas chromatography–MS (GC‒MS), liquid chromatography–MS (LC‒MS), and capillary electrophoresis–MS (CE-MS)^6^. However, despite the progress made in the last decade, many metabolites are still uncharacterized, and the statistical validation for large-scale spectral assignation is limited^7,8^.

In cancer research, metabolomics has proven to be an important omics approach that is able to identify the molecular pathways underlying the tumour phenotype as well as clinically useful markers. The literature on head and neck carcinomas has illustrated the potential of this approach as a diagnostic and prognostic tool using serum/plasma samples, saliva, urine, tumour tissues, cell lines, from patients before or after radio-, chemo- or concurrent radio/chemotherapy (references in Supplementary Table S1). The results obtained from these tumours have shown a complex metabolic profile that may reflect a range of processes^9^, such as both a low^10^ or a high^11–14^ dependence on the glycolytic pathway; increased glutaminolysis/glutamate metabolism^14–17^; a large influx of amino acids into the citric acid cycle^15^; altered choline metabolism that may indicate membrane synthesis during active proliferation^10,14,15,17–19^ or lipolysis to deliver energy for tumour growth^11,15,20^.

Alterations in many oncogenes and tumour suppressor genes, including *TP53*, *MYC* and *RAS*, persistent activation of PI3K–Akt signalling, and enhanced mTOR activity, are necessary to meet the high demands of proliferating cells and place metabolic switching at the centre of malignant transformation rather than as a secondary consequence. In head and neck carcinomas as well as in other tumour types, various genetic alterations have been described that contribute to a dysregulated metabolic phenotype. For example, *TP53* mutations promote the consumption of glucose, which is the primary substrate for the generation of ATP and an essential source of carbon for synthesizing other macromolecules^21^. Changes in EGFR/Ras signalling also induce increased consumption of glucose and glutamine for anabolic processes and promote autophagy and extracellular protein and lipid sequestration^22^. The Ras/Raf/MAPK pathway regulates many other pathways, such as the phosphatidylinositol kinase (PI3K-Akt-mTOR) pathway (reviewed by^23,24^, and induces phosphorylation and stabilization of the transcription factor Myc. The final targets of these pathways, including Myc, are connected to energy production (glycolysis and glutaminolysis), anabolic reactions^25^, the expression of glycolytic enzymes, activation of reductive carboxylation of α-ketoglutarate, and the production of citrate for fatty acid synthesis and lipogenesis (reviewed in^23,24,26^.

Head and neck (HN) tumours, the most common type being squamous cell carcinomas (SCCs), affect the epithelium of distinct head and neck topologies, including the pharynx and larynx, and more frequently the oral cavity^27^ and show different clinical courses. The five-year survival rate is low in HNSCC, although a significant increase in the disease-specific survival of patients has been observed in recent years^28–30^ One reason for the low survival is that patients with tumours in early stages frequently exhibit few symptoms, resulting in delayed diagnosis and severe morbidity. Even if an early diagnosis is made, recurrence—a frequent event associated with a poor survival prognosis in HNSCC—is unpredictable. Clinically and histologically similar lesions can also show significant differences in behaviour and responses to therapy, both of which are influenced by tobacco use and other risky lifestyles, infection with human papillomavirus (HPV), tumour mutation/expression profile and host factors related to carcinogen/drug metabolism or immune mechanisms^31–36^. The oral microbiota can potentially also contribute to a tumour-permissive or a tumour-protective environment able to modulate susceptibility and the course of the disease^37^. Such variables partially answer why individuals with similar diseases have different clinical outcomes, although the mechanisms underlying several of these associations are still not fully determined. Metabolomics has emerged as a promising tool that may contribute to answering this question and improving our understanding of the mechanisms driving HNSCC development and progression.

The present study aimed to analyse the plasma metabolic profile of patients with oral squamous cell carcinoma (OSCC) and controls and to compare patients with metastatic and primary tumours at different stages and subsites of disease using NMR and MS methodologies. To our knowledge, few studies have investigated the OSCC metabolome of different disease stages, and only one used two methodologies (MS and NMR) to evaluate saliva^38^. Therefore, the present study is the first report on the metabolome of different OSCC stages and subsites, in which plasma samples were analysed by NMR and MS and the results compared from replicates collected in diverse institutions at different times.

## Results and discussion

### Dataset

#### OSCC patients show a low body mass index, which suggest an altered metabolic state leading to the release of nutrients in blood and ultimately to tumour growth

A total of 72 OSCC patients from four institutions were enrolled in the present study: 64 (88.9%) males and 8 (11.1%) females with an average age of 58.6 years, range 42–80 years. Of them, 55 (77.5%) were current smokers, and 31 (43.7%) were current drinkers (Table 1; Supplementary Table S2). Specific subsites included 28 (38.9%) oral tongue (C02), 41 (56.9%) floor of mouth (C04), 2 (2.8%) cheek mucosa (C06), and 1 (1.4%) gum (C03). Regarding pathological TNM (pTNM) staging, 35 (48.6%) patients were lymph node negative (N0), and 37 (51.4%) were lymph node positive (N+); 30 (41.6%) cases were classified as T1T2 and 42 (58.4%) cases as T3T4. For 15 nonsurgical cases, clinical TNM staging was used. Sixty-one controls were also included in the present study: 50 (82.0%) males and 11 (18.0%) females with an average age of 58.2 years, range 37–78 years. Of them, 10 (16.4%) were current smokers and 34 (55.7%) were current drinkers (Table 1; Supplementary Table S3).

**Table 1:** Clinicopathological data of 72 patients with oral squamous cell carcinoma and 61 controls. Most patients are male, older than 40 years, current smokers and alcoholic, have large tumors derived from tongue C02 and floor of mouth C04 subsites with nodal metastases.

Parameter	Group	Patient	Control
		N (%)	N (%)
Gender	Male	64 (88.9)	50 (82.0)
	Female	08 (11.1)	11 (18.0)
Age at diagnosis (years)	Median	58.6	58.2
	Range	42–80	37–78
BMI (kg/m^2^)—technician measurements	Median	23.4	
	Range	15.2–34.7	
BMI (kg/m^2^)—self-reported	Median	22.8	27.8
	Range	13.6–32.4	17.6–45.7
Smoking	Current	55 (77.5)	10 (16.4)
	Former	16 (22.5)	32 (52.5)
	Never		13 (21.3)
	Passive smoker		06 (9.8)
	NA	01	
Alcohol abuse	Current	31 (43.7)	34 (55.7)
	Former	40 (56.3)	21 (34.4)
	Never		06 (9.8)
	NA	01	
Subsite	Oral tongue (C02)	28 (38.9)	
	Floor of mouth (C04)	41 (56.9)	
	Cheek mucosa (C06)	02 (2.8)	
	Gum (C03)	01 (1.4)	
TNM Classification	T1	13 (18.0)	
	T2	17 (23.6)	
	T3	12 (16.7)	
	T4	30 (41.7)	
	N0	35 (48.6)	
	N1	07 (9.7)	
	N2	28 (38.9)	
	N3	02 (2.8)	

The median body mass index (BMI) of patients based on technician measurements was 23.4 kg/m^2^ (range from 13.6 to 32.4 kg/m^2^), and BMI based on self-reports was 22.8 (range from 15.2 to 34.7 kg/m^2^), which shows good agreement between them. The self-reported BMI of controls was 27.5 kg/m^2^ (range from 17.6 to 45.7 kg/m^2^), which was higher than the self-reported BMI of patients (t test; *p* < 0.0001). This difference may be partially explained by difficulty eating due to cancer pain. This may also be explained by the number of current and former smokers among patients (n = 55 and n = 16) and controls (n = 10 and n = 32), considering that smoking and smoking cessation have been associated with lower and higher BMIs, respectively, although their effects appear to be modest^39^. Another explanation is that an altered metabolic state in cancer patients may lead to weight loss (consequently to a reduced BMI) and to tissue wasting with the release of nutrients in the blood, which ultimately can promote tumour growth^40^.

### ^1^H NMR

#### Plasma NMR metabolic profiles discriminate OSCC patients and stage subgroups from controls

A total of 47 OSCC patients and 49 controls were analysed by NMR (Supplementary Tables S2 and S3), 53 metabolites were identified, and 22 were shared between different subgroups (Supplementary Table S4). The 47 patients included 28 N0, 19 N+, 21 C02, 25 C04, 22 T1T2 and 25 T3T4. The reason for the smaller number of cases analyzed by NMR than those by MS was that most NMR experiments were performed after MS analyses and the available amount of plasma necessary for NMR was insufficient in 12 control and 14 patient cases.

**Figure 1:**
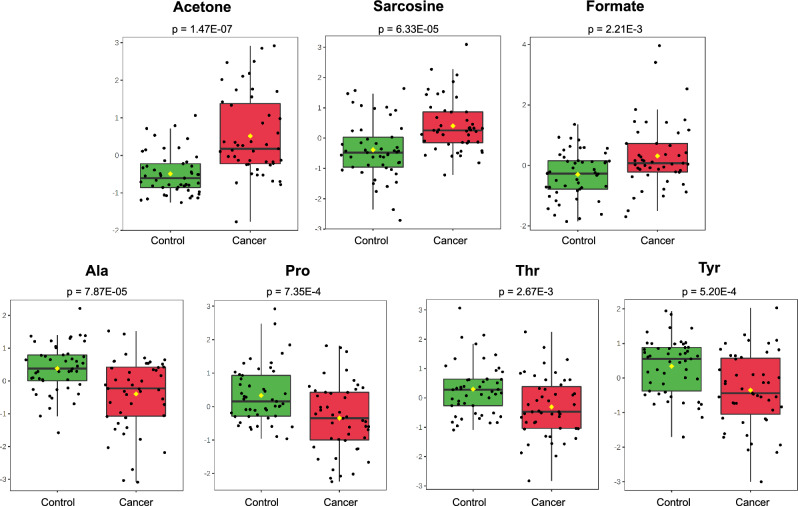
Plasma NMR metabolic profiles discriminate OSCC patients from controls and suggests elevated ketogenesis, active oxidative metabolism and epigenetic alterations in patients. T-test *p* values (*p* ≤ 0.05) and box plots of acetone, sarcosine, formate, alanine (Ala), proline (Pro), threonine (Thr) and tyrosine (Tyr) in cancer and control samples.

Although the number of cases was low in several subgroups, statistical analyses (t test, volcano, and PLS-DA) showed that 10 metabolites (acetate, acetoacetate, acetone, alanine, creatine, formate, proline, sarcosine, threonine, tyrosine) could distinguish OSCC patients (Fig. 1) and both the early-stage and advanced-stage cancer subgroups from controls (Supplementary Table S5; Supplementary Figs. S1−S3), but no differences were detected between subsites C02 and C04, or between stages N0 and N+, or T1T2 and T3T4**.** The expression profile of seven metabolites was shared by cancer and T3T4 stage (acetone, alanine, formate, proline, sarcosine, threonine and tyrosine), whereas acetate, creatine (both with lower levels) or acetoacetate (with higher levels) were only observed in N0, T1T2 or N+ cases, respectively, compared to controls. The Supplementary Figure S4 shows *a* representative unprocessed spectrum and the spectral region containing the alanine peak after analysis of plasma samples.

The PLS-DA classification presented satisfactory values for consistency and predictability of the models (R2 and Q2, respectively). The *p* values based on validation by permutation ranged from ≤ 0.001 to 0.023. The highest Q2 was found in the comparison of T2T3 with controls (0.4) (Supplementary Fig. S5).

**Figure 2:**
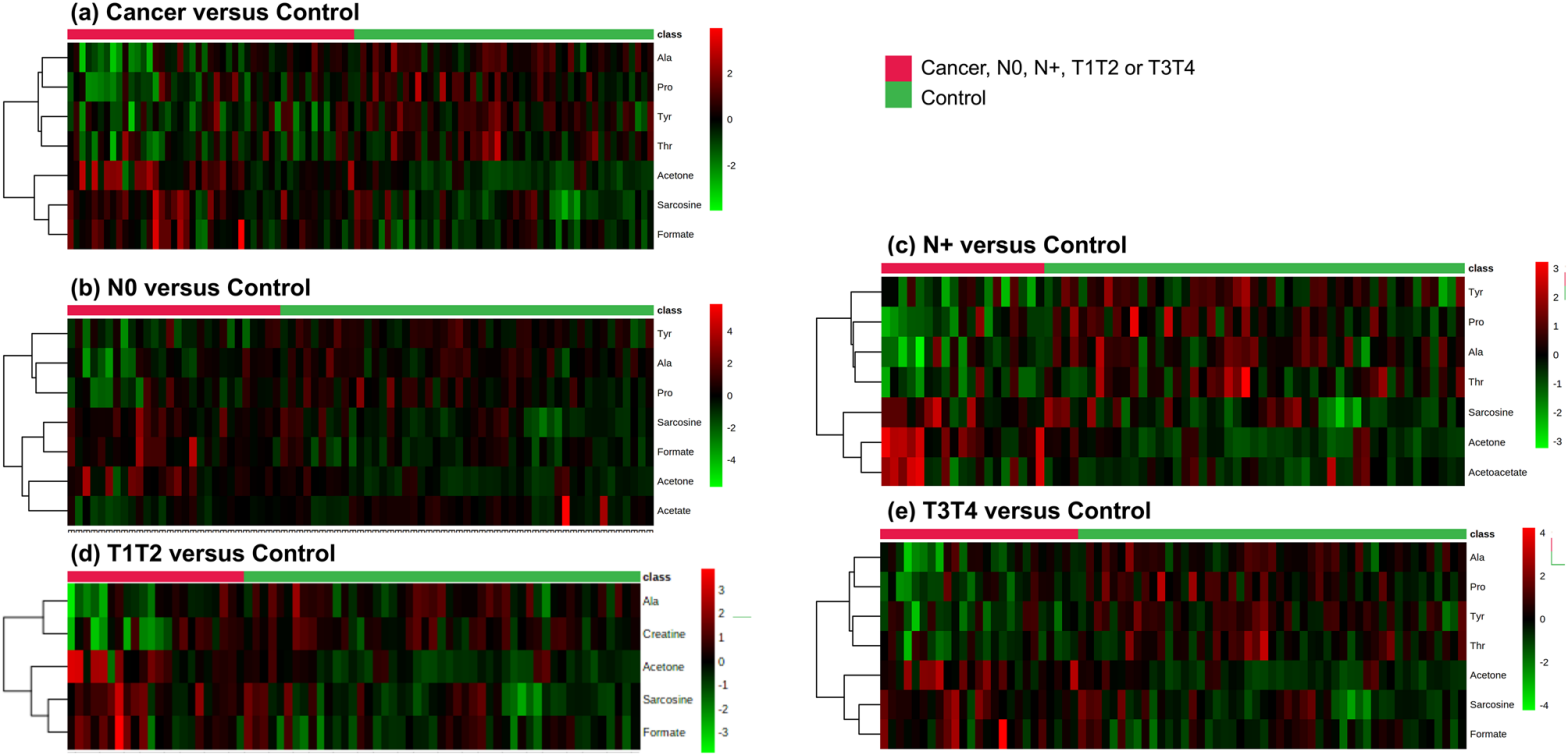
Plasma NMR metabolic profiles discriminate OSCC stage subgroups from controls. Heatmap using the top metabolites differentially expressed in (**a**) Cancer versus Control; (**b**) N0, (**c**) N+, (**d**) T1T2, (e) T3T4 versus Control. Top bar: cancer samples in red, control samples in green. Vertical bar: the colors represent the mean concentration of metabolites—red and green values indicate over- and underexpression, respectively (t-test, *p* < 0.05).

**Figure 3:**
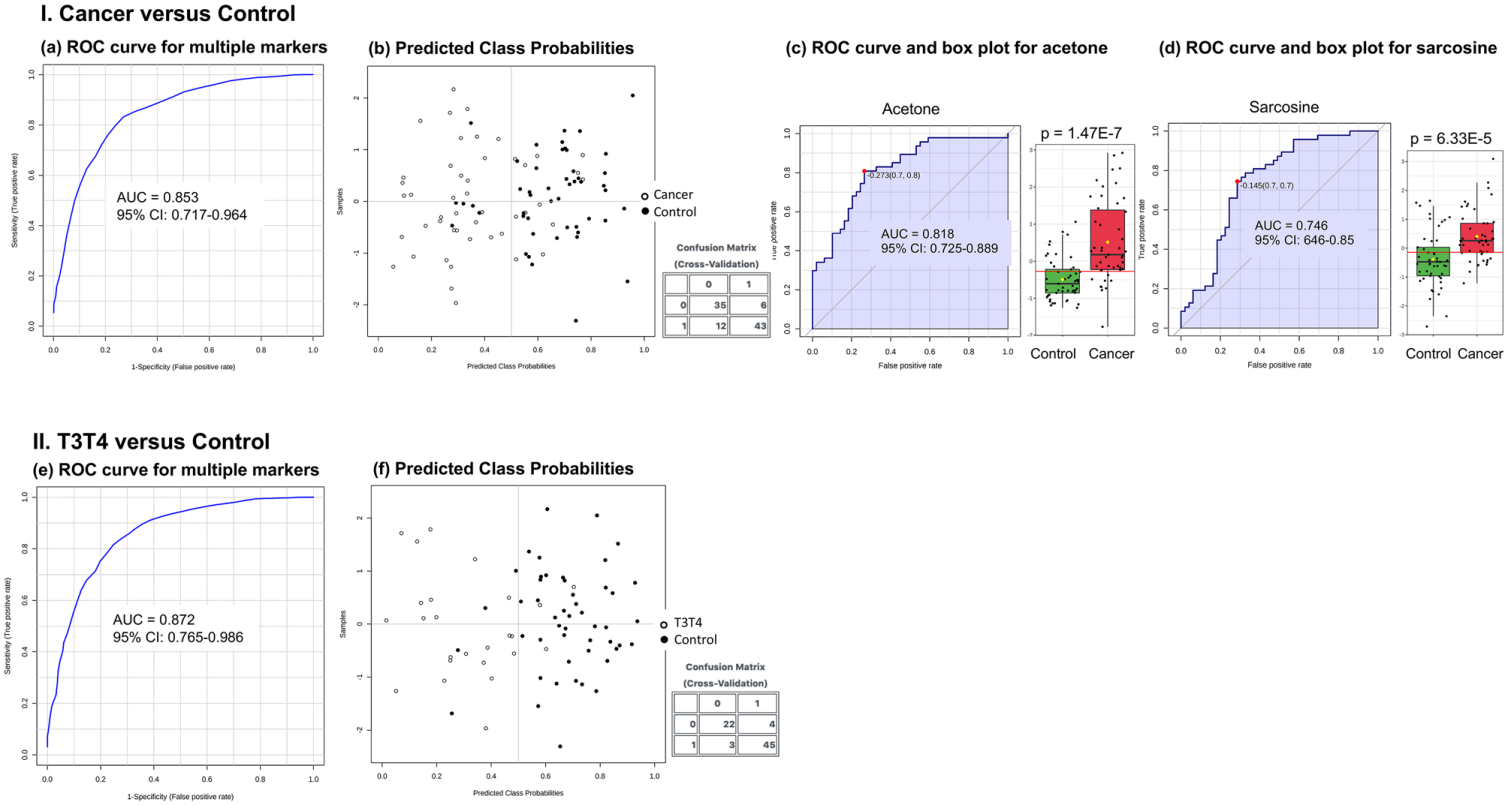
Multivariate ROC curve based on the NMR data was able to discriminate cases and subgroups from controls and showed a higher prediction power for larger tumours. I. Cancer versus Control. (**a**) ROC curve for multiple markers; (**b**) Plot of predicted class probabilities for all samples; (**c**) Univariate ROC curve and box plot of acetone; (**d**) Univariate ROC curve and box plot of sarcosine II. T3T4 versus Control. (**e**) ROC curve for multiple markers; (**f**) Plot of predicted class probabilities for all samples.

The heatmap generated with all identified metabolites or the top 7 differentially expressed metabolites (t test, *p* < 0.05) and with the higher VIP values (VIP scores > 1.0) confirmed the metabolic pattern observed on PLS-DA analysis and showed a clear segregation between patients, both the early-stage and advanced-stage cancer subgroups (except N0), and controls (Fig. 2; Supplementary Fig. S6). Multivariate ROC curve analyses using the entire set of variables were also able to discriminate cases and subgroups from controls. The pairwise comparisons of controls and total patients or N0, N+, T1T2, and T3T4 cases generated AUC values of 0.853, 0.811, 0.796, 0.757, and 0.872, respectively, showing a better prediction power for larger tumours (Fig. 3). For the same comparisons, the sensitivity and specificity were 74% and 85% (cases vs. controls); 0.79 and 0.88 (N0 vs. controls); 0.68 and 0.90 (N+ vs. controls); 0.64 and 0.84 (T1T2 vs. controls); and 0.88 and 0.93 (T3T4 vs. controls), respectively, confirming better performances for larger tumours (Fig. 3a,b,e,f; Supplementary Fig. S7). Univariate ROC curve and box plot of acetone and sarcosine are presented in Fig. 3c,d.

#### Plasma NMR metabolic profile of OSCC patients indicates elevated ketogenesis and fat metabolism

To our knowledge, three studies have investigated the OSCC metabolome of different disease stages by NMR. Tiziani et al.^11^ analysed serum from 15 patients and 10 controls and detected 23 differentially expressed metabolites in OSCC samples compared with controls and 19 in late-stage compared with early-stage cases. In turn, Lohavanichbutr et al.^38^, analysing saliva from 101 patients and 35 controls, identified 20 differentially expressed metabolites in cancer cases, 15 of which were also differentially expressed in T1T2 versus controls. Zhou et al.^41^, different from the other two studies, analysed 60 plasma samples from patients with OSCC, oral premalignant leukoplakia and controls to identify patients early, regardless of tumour location or pathological grade. Their results revealed a model able to differentiate cancer from premalignant and healthy cases.

Two metabolites with the same expression pattern were detected by Tiaziani and by the present study in late stages (acetone and alanine) and 4 in total cancer patients (acetone, alanine, sarcosine and tyrosine). Comparing the Lohavanichbutr group’s results and our study results, 4 metabolites were associated with cancer (alanine, proline, tyrosine and threonine), but no compounds in the T1T2 stages were shared by the two studies.

The metabolites cited above and others, acetoacetate, acetone, formate and sarcosine, showed high levels in our patients, and the opposite was found for acetate, alanine, creatine, proline, threonine and tyrosine (Fig. 1). Similar NMR results for acetone and threonine were observed by Gupta et al.^20^ in serum from OSCC cases. In accordance with our data, Boguszewicz et al.^42^ found increased serum levels of threonine and tyrosine after radiotherapy, which could represent a return to normal concentrations resulting from the treatment. The authors also found increased levels of acetoacetate and acetone and low levels of alanine associated with weight loss in patients treated with radio/chemotherapy, a result that is potentially consistent with our data of decreased BMI in OSCC cases^43^. In HNSCC tumour and lymph node metastatic tissues, a diverse panorama was viewed by Somashekar et al.^15^, including elevated levels of alanine and tyrosine, which may be caused by different influx and efflux rates from and to serum or by changes in biosynthetic pathways.

Circulating ketone bodies (acetone, acetoacetate [AcAc] and β-hydroxybutyrate [BHB]) are derived from acetyl-coenzyme A (ace-CoA) predominantly via lipid byproducts, although a small proportion is synthesized from amino acid metabolism^44^. Ketogenesis mainly occurs in the liver using fatty acids for energy in response to fasting conditions. The free fatty acids obtained from adipocytes are released into the bloodstream and enter liver cells, where they undergo β-oxidation to form ketone bodies or are esterified to phospholipids and triacylglycerol^45^. For this reason, high levels of ketone bodies are observed during weight loss due to low-energy diets or in various acute or chronic health conditions and are an indication of elevated fat metabolism^46^. In addition to nutrients, hormonal events affect ketogenesis: glucagon and catecholamines promote the process, and insulin inhibits it^47^. The starvation state in chronic alcohol abuse also contributes to ketogenesis, decreasing insulin secretion and increasing the production of glucagon, growth hormone, cortisol, and catecholamines. The result is downregulation of gluconeogenesis and upregulation of lipolysis. Therefore, alcohol use disorder may affect cellular energetics, leading to low glucose metabolism and a high metabolism of acetate and consequently to increased levels of plasma ketone bodies^48,49^. All these data support our results with respect to higher concentrations of ketone bodies and lower levels of ketonic amino acids in the plasma of patients (mostly alcoholics) than in controls.

The role of ketone bodies in cancer is emerging from studies in animal models and human cohorts, but the results have led to divergent conclusions^50^. For example, there is evidence that cancer cells are not efficient in metabolizing ketone bodies for energy and that ketones slow tumour cell proliferation, inhibiting glycolysis or lactate export from the cell and thus promoting anticancer effects^51^. Other studies suggest that cancer-associated fibroblasts produce ketone bodies to support tumour growth and metastasis^52,53^. Ketone bodies, particularly the ketone body β-hydroxybutyrate, exhibit signalling roles as inhibitors of histone deacetylases, thus potentially affecting gene expression via chromatin modifications^54,55^. In addition, ketone bodies have anti- and proinflammatory effects^50^ and may induce oxidative stress-dependent mechanisms^56^, which are potentially related to tumorigenesis.

#### OSCC patients show high plasma levels of formate and sarcosine, which suggest an active oxidative metabolism and epigenetic alterations

In addition to ketone bodies, another important observation in our results was the increase in formate and sarcosine levels in patient plasma (Fig. 1). High rates of formate accumulation are observed in oxidative cancers and are mainly derived from serine catabolism^57^. Formate can directly react with tetrahydrofolate/THF and enter the one-carbon cycle for the synthesis of purines^58,59^. Sarcosine (N-methylglycine) is a nonproteinogenic amino acid involved in the metabolism of glycine, which in turn can also provide one-carbon units and contribute to the synthesis of purines^59–61^.

Altered levels of sarcosine in serum/plasma and urine have been observed in several diseases, such as type 2 diabetes^62–64^, narcolepsy^65^, allergic rhinitis^66^, chorioretinopathy^67^, and even in healthy persons in response to exercise^68^ or in association with alcohol consumption^69^. Walters et al.^70^ showed in plasma from rats and humans that sarcosine is reduced with ageing and elevated by dietary restriction. As sarcosine activates macroautophagy in cultured cells and in vivo, these authors concluded that sarcosine contributes to increase resistance to stress and to proteostasis, mechanisms that decline with age. From their data, Walters and collaborators suggested that sarcosine is a biomarker of ageing and restrictive diets in mammals. In fact, the low levels of plasma non-essential and essential amino acids in our cancer cases may also be indicative of a restrictive diet.

Positive associations of sarcosine or sarcosine metabolism-related proteins with cancer have been observed in pheochromocytoma/paraganglioma^71^, renal cell carcinoma^72^, breast cancer^73^, and particularly in prostate cancer, in which it is considered a potential biomarker^60,74–76^. Although sarcosine has not been highlighted by most head and neck cancer studies, it was not surprising to observe high levels of this amino acid in the plasma of our OSCC patients considering that the disease has features of chronic alcohol abuse, starvation state and weight loss.

The biological mechanisms underlying sarcosine and tumorigenesis are not well defined but may involve the balance of methylation/demethylation and proliferation/apoptosis. Sarcosine is synthesized by two pathways interconnected by the enzyme GNMT (glycine N-methyltransferase): GNMT converts S-adenosylmethionine (SAM) to S-adenosylhomocysteine (SAH), which allows glycine methylation to sarcosine. Considering that methyl groups are transferred from SAM to many compounds, the deficiency of methyl-group donors or coenzymes of methyl group metabolism may affect SAM levels, thus altering DNA methylation and gene expression^60^. In prostate cells, Strmiska et al.^77^ showed that sarcosine is an important member of this process, acting as an epigenetic modifier and hence contributing to tumorigenesis.

### MS

The main cohort (set A) analysed by MS consisted of plasma samples from 61 patients and 61 controls from four institutions (25 of subsite C02 and 33 of subsite C04 and 28 N0, 33 N+, 24 T1T2 and 37 T3T4 stages) (Supplementary Tables S2 and S3). Of them, 36 samples from patients and 49 from controls were also analysed by NMR. Statistical analyses of metabolites, including t test, PLS-DA and ROC curve, were performed comparing total cases and controls, different cancer anatomical subsites and stages: C02 versus C04; each stage (N0, N+, T1T2, and T3T4) versus controls; each stage to the other (N+ vs. N0; T1T2 vs. T3T4).

**Scheme 1:**
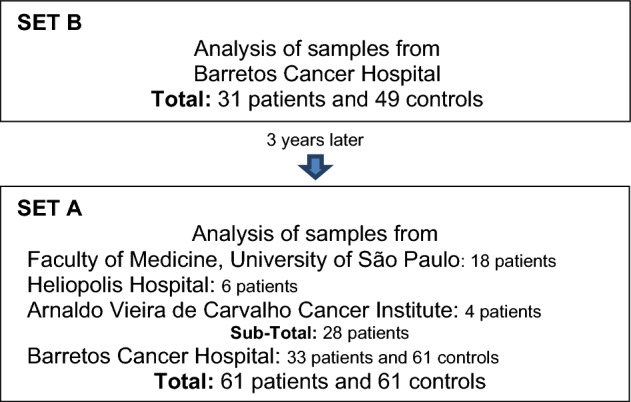
Analysis of set A and B samples.

To investigate the reproducibility and interinstitutional heterogeneity regarding sample processing, the assays were performed with the same plasma aliquots from 61 patients and 61 controls of four institutions (set A) and with 31 patients and 49 controls (set B, all of them present in set A) from a single institution (Barretos Cancer Hospital) (Supplementary Tables S2 and S3). The assays with set A and B were carried out 3 years apart (Scheme 1).

#### All patients and subgroups versus controls (set A)

As mentioned above, early diagnosis of oral carcinoma is critical for guiding clinical decisions, minimizing the adverse effects of treatment and determining prognostics. In contrast to late stages, patients during the initial stages of OSCC are often underdiagnosed, even after a careful physical examination. Consequently, most patients have advanced disease at the time of diagnosis, which indicates that early detection of this tumour is challenging and that biomarkers with high sensitivity and specificity are still limited^33,35^.

The targeted MS approach used in the present study allowed the quantification of 188 metabolites in plasma samples, 131 of which were selected for analysis after the filtering process. Several additional variables were also investigated, including metabolite ratios and the sums of aromatic (AAA), branched (BCAA), essential (EAA), nonessential (NEAA) and selected amino acids, acylcarnitines, lyso-phosphatidylcholines (lysoPC), phosphatidylcholines (PC aa and PC ae), and sphingomyelins (SM), which totalled 104 sums and ratios (Supplementary Table S6). Therefore, the total number of variables amounted to 235 metabolites and sums/ratios.

#### Plasma MS metabolic profile showed a small but abrupt shift from normal to early tumour phases, followed by pronounced changes in more advanced tumours

Statistical analyses of the comparison of the total group of patients with controls using a t test (*p* < 0.02) identified 72 plasma metabolite biomarker candidates, a set of them with *p* values lower than 8.5e−5, such as SM C24:1, PC aa C36:6, SM C16:0, Ala, PC aa C40:3, C5 and PC aa C42:4, and the ratios SM C24:1/Met/PC aa 40:3; (Ala/Gln)/(Tyr/Phe); Ala/PC aa C40:2 and Ala/Gln (Supplementary Table S7).

**Figure 4:**
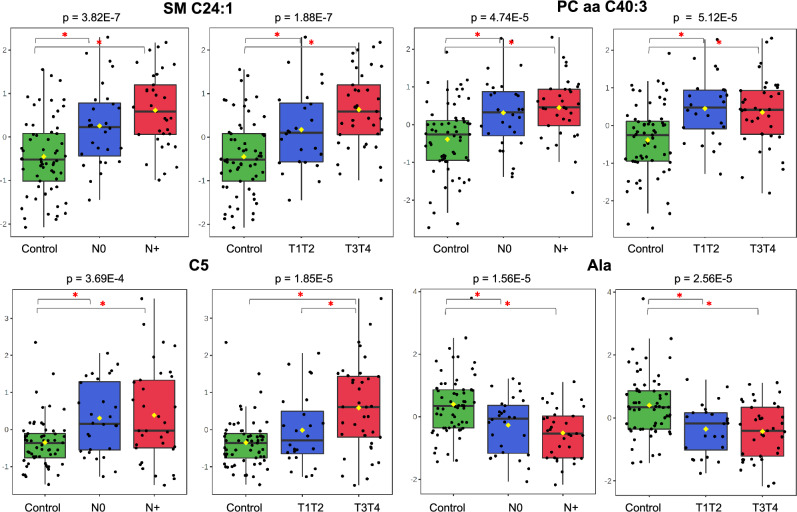
Plasma MS metabolic profile shows a shift from normal to early tumour phases followed by pronounced changes in more advanced tumours. Sphingomyelin SM C24:1, phosphatidylcholine PC aa C40:3, acylcarnitine C5 and amino acid Ala levels (Y-axis) in controls and in N0, N+, T1T2, T3T4 stages (X-axis) (one-way ANOVA, Fisher’s LSD).

**Figure 5:**
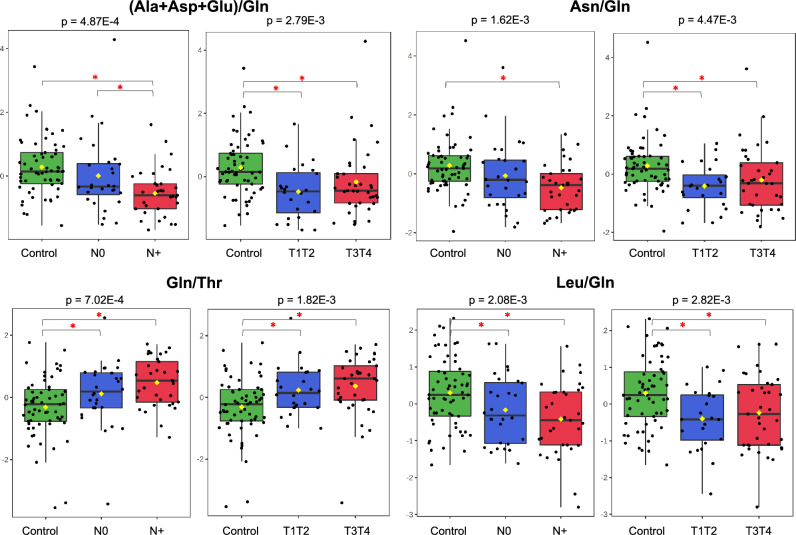
As deduced by the metabolite ratios, several amino acid levels based on the MS data show an expression pattern opposite to that of glutamine. (Ala + Asp + Glu)/Gln; Asn/Gln; Gln/Thr and Leu/Gln levels (Y-axis) in controls, and in N0, N+, T1T2, T3T4 stages (X-axis) (one-way ANOVA, Fisher’s LSD).

Advanced-stage cases (N+ or T3T4) versus controls showed overexpression of several phosphatidylcholines, acylcarnitines and sphingomyelins (Supplementary Tables S8 and S9). The same was observed for most (but not all) metabolites and ratios in plasma from patients with small tumours or lymph nodes clinically negative for metastases (N0 or T1T2). Although fewer biomarker candidates have been detected in this early-tumour group (Supplementary Tables S10 and S11), the results showed a small and abrupt shift in the plasma metabolome from normal to initial tumour phases, followed by progressive changes in more advanced tumours (Fig. 4). As deduced by individual levels and ratios, several amino acids follow this trend but with an expression pattern opposite to that of glutamine (Fig. 5). Najumudeen et al.^78^ demonstrated that *KRAS* activation promotes the expression of the glutamine transporter SLC7A5 (recently observed as upregulated in head and neck carcinomas by Li et al.,^79^). From their data, Najumudeen et al. suggested a role for glutamine as an exchange factor that promotes the uptake of extracellular amino acids and supports the disease progression.

The volcano plot, combining statistical significance from the t test with the magnitude of the fold change (FC), confirmed C5 as an important feature in the comparison of the total group of patients with controls (raw *p* value = 7.16E−5). Low raw *p* values (< 3E−6) were also observed in the advanced-stage subgroups for C5 (N+) and for the ratio (Tyr/Phe)/(Met/PC aa C40:3) (T3T4) (raw *p* value = 5.78E−8) (Supplementary Figs. S8–S10). The volcano plot for N0 and T1T2 cases identified the Asn/Asp, (Asn/Asp)/Glu and Gln/Glu/Asp ratios as important features, but with lower raw *p* values, confirming that these subgroups are metabolically closer to the normal condition (Supplementary Figs. S11 and S12).

PLS-DA also demonstrated better group separation and cross-validation results with higher values for consistency and predictability of the models (R2 and Q2, respectively) for advanced-stage cases versus controls. The highest Q2s were found in the comparison of N+ and T3T4 with controls (Q2 = 0.6), and the *p* values based on validation by permutation ranged < 0.001 (Supplementary Figs. S9 and S10). Lower Q2 were observed for early-stage cases versus controls (Supplementary Figs. S11 and S12). The same trend for group separation was observed by hierarchical clustering analysis using the top differentially expressed metabolites by t test (*p* < 0.05), which again revealed a sharp sample grouping for the total group of patients or advanced cases against controls but not for early tumours (Supplementary Figs. S13–S15).

A multivariate ROC curve analysis to discriminate cases from controls using the entire set of variables was also generated, and the area under the curve (AUC = 0.886, 95% confidence interval/CI: 0.822 to 0.945) indicated a good diagnostic ability. The plot of predicted class probabilities was able to classify 46 cases out of 61 and 52 controls out of 61, achieving a sensitivity of 75% and a specificity of 85%. The univariate ROC curve of the three best biomarker candidates—SM C24:1/Met/PC aa 40:3; (Ala/Gln)/(Tyr/Phe); and Met/PC aa 40:3—showed AUCs of 0.839, 0.782 and 0.770, respectively (Supplementary Fig. S16). For advanced-stage tumours, the AUC was higher (0.92), and the plot of predicted class probabilities showed high values of sensitivity and specificity (88% and 89% for N+ and 78% and 89% for T3T4, respectively). As expected, lower values of AUC (0.791 and 0.722, respectively) and lower sensitivity and specificity values (68% and 87% for N0 and 63% and 75% for T1T2, respectively) were obtained for N0 and T1T2 cases. Supplementary Figures S17 and S18 present ROC curves for multiple markers, plots of predicted class probabilities and univariate ROC curves and box plots for the top biomarker candidates in the comparisons of stages N+, T3T4, N0, T1T2 and controls.

#### Tongue and floor of mouth subsites are metabolically similar

To identify differences in metabolite concentrations between the C02 and C04 subsites, t tests and volcano plots (fold change threshold 2.0; *p* value threshold 0.05) were performed, but no significant differences were found. PLS-DA detected several VIP scores > 1.0 (as the VIP score of SDMA), although the estimate of the predictive ability of the model (Q2) calculated via cross validation showed negative values, which means that the model is not predictive (Supplementary Fig. S19). Multivariate ROC curve was generated, and the area under the curve (AUC = 0.578, 95% confidence interval/CI: 0.384 to 0.745) and the sensitivity and specificity values (56% and 61%, respectively) were obtained, confirming that C02 is metabolically close to C04 (Supplementary Fig. S20). The univariate ROC curve of SDMA also showed low AUC values. To our knowledge, the present study is the first to analyse the C02 and C04 subsites of oral carcinomas in a metabolomic context.

#### Subgroup from a single institution (set B)

*Plasma metabolite profile is independent of sample storage time and population background*. Despite the smaller number of samples, the comparison of cases and controls of set B showed basically the same metabolite pattern of set A. The data were confirmed by t test, PLS-DA, volcano, hierarchical clustering and ROC analyses (Supplementary Tables S12 and S13; Supplementary Figs. S21–S23). Therefore, the metabolite concentrations were independent of the freezing–storage time and the institution of origin or population background. Considering that the operating procedure and freezing scheme were the same in all centers, a differential preanalytical freezing delay time with impact on the stability of metabolites was avoided, an important issue in metabolomics as commented by^84^.

#### Circulating plasma metabolites in OSCC patients

*Oral carcinomas exhibit high plasma sphingolipid levels that suggest a crosstalk between cancer and normal cells for lipid or exosome release*. Within the patient group, there were increased levels of sphingomyelins, as well as of half of the phosphatidylcholines, whereas no significant changes were observed in lysophosphatidylcholine and hexose concentrations. The levels of aromatic, branched, essential or glucogenic amino acids were reduced, whereas those of glutamine, ornithine, and serine were relatively increased. Several ratios of carnitines, amino acids and phosphatidylcholines also exhibited strong relationships with the case/control groups.

Sphingolipids are preferentially derived from the condensation of the amino acid serine and the lipid palmitate, which, after sequential reactions, generates ceramide and subsequently sphingomyelins (SMs) by the insertion of a choline group into ceramide^80,81^. SMs are key structural components of cell membranes and regulators of the cell cycle, apoptosis, angiogenesis and inflammation, with a potential role in several diseases, including cancer^82^. In head and neck carcinomas, the literature has referred an important role of sphingolipid metabolism in tumour development, including the regulation of cell growth and apoptosis^83^. Literature data have also shown that C16- and C24-ceramide levels are increased in tumour tissues compared with normal matched samples, unlike C18-ceramide, which exhibits lower levels and is also positively related to higher stages of HNSCC tumours^84–86^.

**Figure 6:**
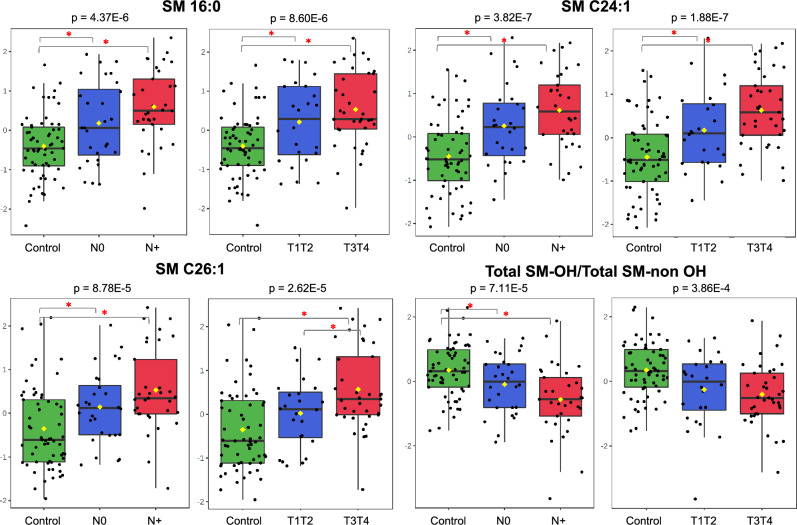
High plasma sphingolipid levels suggest a crosstalk between cancer and normal cells for lipid or exosome release. Sphingomyelins SM C16:0, SM C24:1, SM C26:1 and total SMs (nonOH) showed significantly higher levels (Y-axis) in N0, N+, T1T2, T3T4 stages (X-axis) compared with controls (one-way ANOVA, Fisher’s LSD).

In our plasma samples from patients, SM C16:0, SM C24:1 and SM 26:1 showed significantly higher levels in N0, N+, T1T2, T3T4 stages compared with levels in controls (*p* = 1.9E−7 to *p* = 8.8E−5) (Fig. 6). In addition to a differential synthesis and secretion of SMs, this finding may be due to signals from cancer cells for lipid release from adipocytes or other cells to support their increased membrane synthesis demand. This metabolic symbiosis was already described for adipocytes and cancer cells from breast tumours^87,88^, and the process depends on prior intracellular lipolysis and the release of free fatty acids by adipocytes or on a not yet well-evaluated extracellular lipolysis via lipoprotein lipases secreted by cancer cells^89^. A complementary possibility is raised by the results of Podbielska and collaborators^90^, who observed that the treatment of cell cultures with the proinflammatory cytokines TNF-α and IFN-γ increased sphingolipid levels of secreted exosomes, particularly of C16-, C24-, and C24:1-SM species. As chronic inflammation is an important factor for head and neck tumorigenesis^91^, the high levels of these SM species in plasma exosomes may be induced by cytokines of the tumour microenvironment.

The type, abundance and hydroxylation of SMs determine the cell membrane properties, such as the ability to adapt to temperature and tension changes^92,93^. It has also been proposed that the SM hydroxylation pattern can affect the regulation of G-proteins and modulate cell signalling^82^, which makes sense considering the presence of the sphingolipid-binding motif (SBM) in G protein-coupled receptors^94^. In the plasma of our patients, reduced levels of hydroxylated-SMs [SMs (OH)] and higher levels of nonhydroxylated-SMs [SMs (nonOH)] were observed (Fig. 6), which may reflect differential synthesis or changes in the uptake and release of these SM species, with consequent alteration of membrane compositions and altered activation of G-protein effectors.

#### Patients with oral carcinomas exhibit changes in phosphatidylcholine levels associated with advanced stages, which suggest alterations in choline transporters or in activities of biosynthetic enzymes

The phosphatidylcholines, also key components of cellular membranes along with SMs, showed altered plasma concentrations in OSCC patients. PCs consist of a polar choline headgroup and two fatty acid side chains of varied lengths and degrees of saturation linked to the sn1 and sn2 positions of a glycerol backbone^95^. The PC abundance, fatty acid chain lengths/saturation levels and position of double bonds affect cell membrane properties, such as fluidity, and may contribute to the pathogenesis of many diseases, including cancer^96,97^. In fact, a number of tumours exhibit phosphatidylcholine metabolism alterations, which are caused by abnormal uptake of choline by transporters on the membrane of tumour cells, by altered activity of enzymes in biosynthetic pathways, and by the levels of substrates^98^.

**Figure 7:**
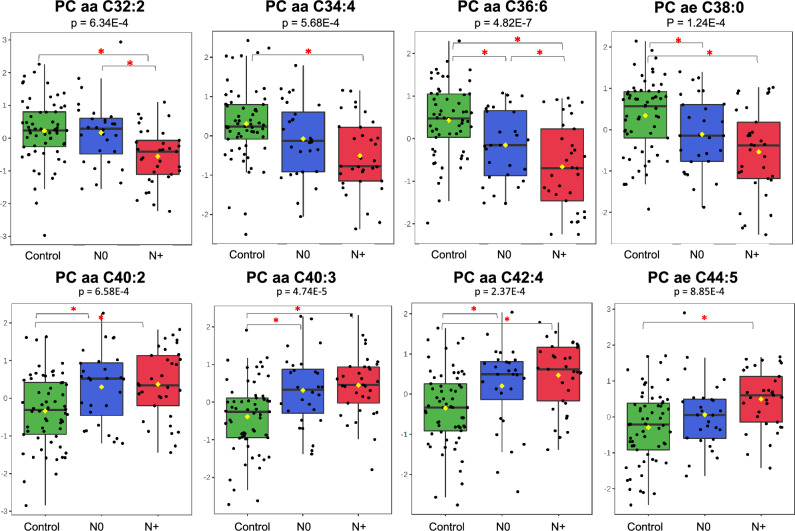
Alterations in choline transporters or in activities of biosynthetic enzymes are suggested by the MS data. Phosphatidylcholines with fatty acid chains totalling 32 to 38 carbons, or totalling more than 38 carbons were present at low or high levels (Y-axis), respectively, in N0 and N+ stages compared with controls (X-axis) (one-way ANOVA, Fisher’s LSD).

While gene/protein expression and enzyme activity are analysed by well-established methods, some lipidomics approaches face certain challenges. Although these approaches, such as the one used by the Absolute*IDQ*® p180 Kit of Biocrates, provide robust measurements of lipid species, their annotations do not take into account the lengths and saturation degree of each fatty acid chain of PCs and therefore cannot differentiate the fatty acids linked to the glycerol backbone. As a consequence, the concentration of a detected lipid may be a sum of two or more isobaric/isomeric species. For example, PC aa C36:6, a phosphatidylcholine that exhibited one of the lowest levels in the plasma of our patients, can represent a group of different species with distinct compositions (e.g., PC(18:3/18:3) vs. PC(14:0/22:6)), different sn-1 and sn-2 positions of fatty acids (e.g., PC(20:5/16:1) vs. PC(16:1/20:5)) and different stereochemistry/bond geometries, such as PC(14:1(9Z)/22:5(4Z,7Z,10Z,13Z,16Z)) vs. PC(14:1(9Z)/22:5(7Z,10Z,13Z,16Z,19Z))^97^. Despite this limitation, the t test and PLS-DA model showed decreased plasma levels of diacyl- and acyl-alkyl-PCs with fatty acid chains totalling 32 to 38 carbons in patients, whereas PCs with fatty acid chains totalling more than 38 carbons were present at high levels (Fig. 7). These findings may be related to alterations in diet and genetic variants that result in changes in the plasma lipid composition^99^. Another explanation would be that the phosphocholine cytidylyltransferase pathway (CDP), which produces mainly PCs with medium-sized chains, is impaired, whereas the liver phosphatidylethanolamine *N*-methyltransferase (PEMT) pathway, which generates PCs with longer polyunsaturated chains, is upregulated^100^. The findings may also be caused, as for sphingomyelins and other lipids, by signals derived from cancer cells that promote extracellular lipolysis or exosome release from adipocytes^101^. A potential stimulus behind this process includes nutrient deprivation, hypoxia^89^ and acidosis^95^, which are all present in the microenvironment of highly proliferative tumours.

#### Evidence of mitochondrial dysfunction and oxidative stress in oral tumorigenesis is supported by the high levels of several acylcarnitines and of methionine sulfoxide to methionine ratio

In addition to providing building blocks for new membrane formation, lipids may act as second messengers and modulators of protein posttranslational modifications and play important roles in regulating the biophysical properties of the cell membrane associated with migration, invasion and metastatic processes^80^. In addition, lipid metabolites are an important energy source, both for immediate and later use. Lipid metabolism involves several processes, including β-oxidation and lipogenesis. The mitochondrial β-oxidation cycle is one of the most important catabolic processes in which fatty acids taken up by cells are converted into acetyl Co-A in the mitochondrial matrix. Acetyl-CoA can thus be used in the synthesis of metabolic intermediates or can also fuel the citric acid cycle for energy production.

To be oxidized, short- and medium-chain fatty acids (SCFAs and MCFAs, chain lengths of 1 to 6 and 7 to 12 total carbon atoms, respectively) permeate the mitochondrial membrane, whereas long-chain fatty acids (LCFAs, chains longer than 12 carbon atoms) require the carnitine shuttle to be transferred to the mitochondrial matrix^102^. For this transport to occur, LCFAs are converted to LCF acyl-CoAs at the outer mitochondrial membrane and are conjugated to carnitine. Acylcarnitines are then translocated across the inner mitochondrial membrane, and once inside mitochondria, carnitines are removed from acylcarnitines, regenerating acyl-CoAs. Carnitines then return to the cytoplasm for another cycle, and acyl-CoAs are sequestered to yield several molecules of acetyl-CoA^103^. Therefore, acylcarnitines regulate the availability of free CoA.

**Figure 8:**
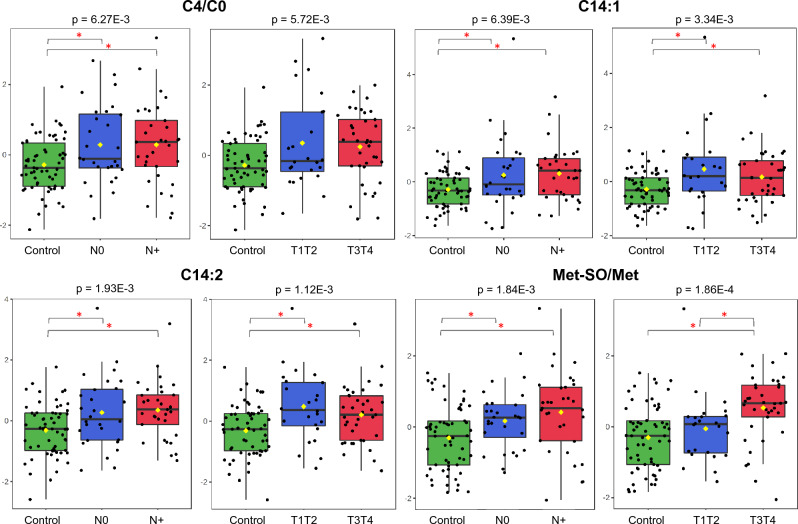
Mitochondrial dysfunction and oxidative stress were observed in the plasma MS data from patients. Acylcarnitine C14:1, C14:2 levels, and C4/C0, Met-SO/Met ratios (Y-axis) were increased in N0, N+, T1T2 or T3T4 stages compared with controls (X-axis), suggesting significant oxidative stress and potentially mitochondrial disfunction in OSCC patients (one-way ANOVA, Fisher’s LSD).

Carnitine is an amino acid derivative that is endogenously formed from lysine and methionine or provided by the diet, whereas acylcarnitines, as mentioned above, are products and substrates of mitochondrial beta-oxidation. The levels of acylcarnitines C4, C14:1 and C14:2 (Fig. 8) and C5 (Fig. 4) were increased, and free carnitine (C0) and C3 levels were decreased in our plasma samples from patients. Reduced concentrations of lysine and methionine were also detected, which may explain the deficiency of C0 in plasma and may indicate an unfavourable nutritional status, corroborating our data on BMI and the data of low levels of free carnitine observed by Takagi and collaborators^104^ in the serum of a sarcopenia group of gastrointestinal cancer patients.

Impaired acylcarnitine profiles are found in inherited diseases of mitochondrial fatty acid oxidation, which are characterized by their accumulation and release into the circulation and by specific dysfunctional/missing enzyme or transporter deficiencies^105^. The consequences for acylcarnitine accumulation, at least in animal models and in vitro experiments, include an increase in cell membrane permeability, apoptosis, disruption of tight junctions and activation of the inflammatory response regulator (NF-kB)^106^. Particularly in relation to C14 acylcarnitine, Rutkowsky and collaborators confirmed that this byproduct of incomplete β-oxidation induced the expression and secretion of proinflammatory cytokines in a dose-dependent manner^107^. The review by Bota et al.^108^ observed that most studies on alcohol use disorders detected altered plasma and tissue levels of the total carnitines (analysing free and acylcarnitines together), particularly in the presence of alcoholic cirrhosis. Langenau and collaborators^109^, using MS to quantify targeted metabolites in a subsample of 2500 individuals from the European Prospective Investigation into Cancer and Nutrition (EPIC)-Potsdam Study, found that alcohol consumption is indeed significantly associated with an increase in serum of long- and short-chain acylcarnitines.

The mean plasma levels of essential and specific glucogenic amino acids were reduced in plasma from our cases compared to controls. These findings may be related to increased demands for energy, biosynthetic units and modulators of redox balance by tumour cells and may explain the decreased median body mass index observed in patients. Among the essential amino acids, methionine (Met) is specifically linked to cancer, given its participation in one-carbon metabolism, which regulates nucleotide, polyamine and lipid synthesis; cellular redox status; and epigenetic modifications^110^. In fact, cancer cells are unable to proliferate in methionine-free medium, a characteristic referred to as methionine dependence, methionine stress sensitivity or the Hoffman effect^111^. Similar to our data, Bian et al.^112^ also found low levels of methionine in the serum of ovarian carcinoma patients. These authors observed that tumour cells consume and outcompete T cells for methionine, which impairs T-cell survival and function and causes a decrease in substrates in one-carbon metabolism.

Methionine residues can be oxidized into methionine sulfoxide (Met-SO) and, depending on its position in the molecule, can alter the physicochemical properties of a protein and therefore modulate its function. The ratio of Met SO/Met is increased in the serum of patients with type 2 diabetes and renal failure and is influenced by chronic smoking, probably due to the high glucose levels that stimulate oxidative stress and to the cigarette smoke, which is rich in free radicals^113^. The plasma of our patients showed an elevated Met-SO/Met ratio compared with controls (Fig. 8), which indicates significant oxidative stress and potentially mitochondrial disfunction. However, independent of the underlying mechanism, a high circulating level of Met-SO may be a potential prognostic tool, as observed by Strand and collaborators in endometrial cancer^114^ and by da Silva and collaborators in multiple myeloma^115^.

#### Biological meaning of metabolite ratios

Several ratios that included amino acids, carnitines, sphingomyelins, phosphatidylcholines and lysophosphatidylcholines showed significant differences between cancer cases and controls (Supplementary Figs. S8–S15). Most ratios reveal the equilibrium between a product and its substrate. For example, the ratios including ornithine, asparagine and proline were deregulated in patients, suggesting that the urea cycle is compromised in these cases. Although no clear biological meaning could be linked to all metabolite ratios, they may show alterations in pathways or potential markers with higher diagnostic and prognostic power than individual metabolites because they depend less on the absolute values, as referred to by Knific and collaborators^116^. This is the case of the (Ala/Gln)/(Tyr/Phe) ratio, which showed the lowest t test *p* value among all underexpressed metabolites in more aggressive tumors (Supplementary Tables S7–S11).

#### Pathway analysis

**Figure 9:**
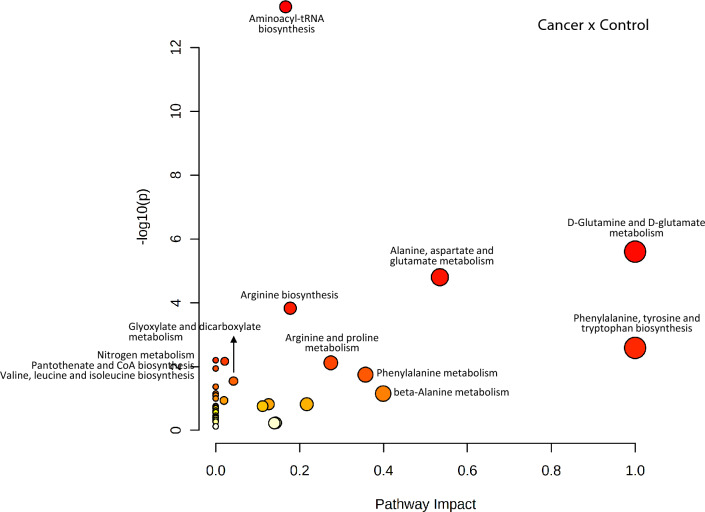
Metabolic pathway enrichment and topology analyses using the MetaboAnalyst platform and 72 important features identified by t-tests (*p* < 0.05) comparing MS data from Cancer versus Control (set A). Each data point of the graph represents a biologic pathway with quantified plasma metabolites.

To obtain a more detailed picture of the OSCC metabolome, pathway enrichment and topology analyses were carried out by MetaboAnalyst software. Influential pathways (number of hits ≥ 2 and Holm *p* value < 0.05) were detected in the comparisons of cancer patients vs. controls (Fig. 9) and in N0, N+ or T1T2 vs. controls (set A): (a) aminoacyl-tRNA biosynthesis; (b) D-glutamine and D-glutamate metabolism; (c) alanine, aspartate and glutamate metabolism; and/or (d) arginine biosynthesis. Using the same scores, only one significantly enriched pathway was identified in the comparison of T3T4 versus controls (Supplementary Figs. S24-S28; Supplementary Tables S14–S18, in dark grey). A similar scenario was observed for cancer patients vs. controls (set B) (Supplementary Fig. S29; Supplementary Table S19). The pathway analysis for C02 vs. C04 was not performed since no significant differences between these subsites were found by t test.

Using set A and set B data and number of hits ≥ 2 and raw *p* < 0.05, other enriched pathways were identified in the total cancer group (most of them were also in different stage groups): phenylalanine, tyrosine and tryptophan biosynthesis; histidine metabolism; glyoxylate and dicarboxylate metabolism; nitrogen metabolism; pantothenate and CoA biosynthesis; arginine and proline metabolism; beta-alanine metabolism; valine, leucine and isoleucine biosynthesis; phenylalanine metabolism; glutathione metabolism; and glycine, serine and threonine metabolism) (Supplementary Tables S14–S19, in light grey). In contrast with other comparisons, the valine, leucine and isoleucine biosynthesis pathway was not observed to have a significant score between the N0 and control groups, and the same was true for nitrogen metabolism and pantothenate and CoA biosynthesis pathways between T3T4 and controls.

In part because of the limited number of metabolites in the targeted analysis and the small number of samples in the subgroups, the results indicated homogeneity independent of the disease stage, although with different impacts and *p* values. Aminoacyl-tRNA biosynthesis and glutamine and glutamate metabolism were the most influential pathways in most OSCC plasma samples, which is expected since they are involved in providing the building blocks of proteins for proliferating cells, as well as for neoplastic and immune cells. Glutamine is a nitrogen carrier that is transported into cells and deamidated to glutamate and ammonia. Glutamate in turn is converted to α-ketoglutarate, which is a citric acid cycle intermediate for energy production. Glutamine also acts as a carbon and nitrogen donor for nucleotides, nonessential amino acids and the antioxidant glutathione and may be used in reductive metabolism for lipid biosynthesis^117^.

Our plasma OSCC samples showed high levels of glutamine in relation to alanine, asparagine, leucine and threonine levels (Fig. 5). Low concentrations of essential and branched-chain amino acids and aspartate were also observed (Supplementary Tables S7–S11, t test, *p* < 0.05). These data suggest an exogenous supply or a response of the organism to the tumour to cover the demands of consuming tissues with different metabolic adaptations, as mentioned above and by other authors^118–120^. The known influx of branched-chain and bulky amino acids in exchange for the efflux of other intracellular amino acids or glutamine also explains the low aspartate, leucine, isoleucine, valine, methionine, phenylalanine, and tryptophan plasma levels^78,121,122^. Branched-chain amino acids are used for protein synthesis. They can also be catabolized to yield acetyl- and succinyl-coenzyme A, which participate in the TCA cycle and contribute to ATP generation^123^.

Ammonia (NH_3_) is a toxic product generated predominantly by nucleotide and amino acid metabolism, especially by glutamine deamination. To prevent toxicity, ammonia is converted into urea through a series of biochemical reactions—the urea cycle—and is excreted in urine. The urea cycle involves catalytic enzymes, transporters and intermediates (ornithine, citrulline, argininosuccinate and arginine) and is regulated by the availability of precursors, mainly glutamine/glutamate and aspartate^124^. Spinelli and collaborators observed that cancer cells may assimilate and redirect ammonia to anabolic pathways, permitting amino acids, such as proline and aspartate, to incorporate this nitrogen^125^. The Takeuchi study also revealed that ammonia can be utilized for glutamate production, leading to cell proliferation under glutamine-depleted conditions^126^. Similar to these findings, our results showed altered pathways involving aspartate, glutamate, arginine and proline, suggesting a deregulated urea cycle that may provide ammonia for tumour demands.

Low concentrations of extracellular alanine were observed in our OSCC samples by using NMR and MS methodologies (Figs. 1, 4). Alanine is required for T-cell activation^127^ and may be taken up by the tumour to supply its own demands while at the same time inhibiting immune defences^128^. Other amino acids, such as arginine^129,130^, asparagine^131^, serine and glycine^132^, and tryptophan also promote T-cell antitumor activity and survival and are extracellular targets for depletion by tumour cells, which leads to their reduced availability in the tumour microenvironment and T-cell functional impairment.

#### Metabolite concentrations by MS and survival

Among 235 variables quantified by mass spectrometry, 11 metabolites or metabolite ratios were potentially considered to have association with overall survival by univariate Cox regression analysis (*p* value < 0.1; Supplementary Table S20) Their discriminatory accuracy and optimal cut-off threshold were thus obtained by the ROC curves and Youden Index (Supplementary Tables S21 and S22). Of these 11 variables, eight have shown significant differences by t-test between controls versus advanced stages, or less discriminating differences versus early stages, apparently exhibiting a continuum of events occurring from normal to metastatic and more aggressive tumor (Supplementary Tables S7–S11). The one that showed the lowest t test *p* values (7.55E-08 to 2.28E-09; Supplementary Tables S8 and S9) in more aggressive tumors versus controls was (Ala/Gln)/(Tyr/Phe) ratio. Three variables (lysoPC a 18:2, PC ae 34:3 and BCAA/AAA) have presented no significant differences by t-test between the total group of patients or subgroups and controls, which suggested that these metabolites may affect survival but not cancer-specific survival.

**Figure 10:**
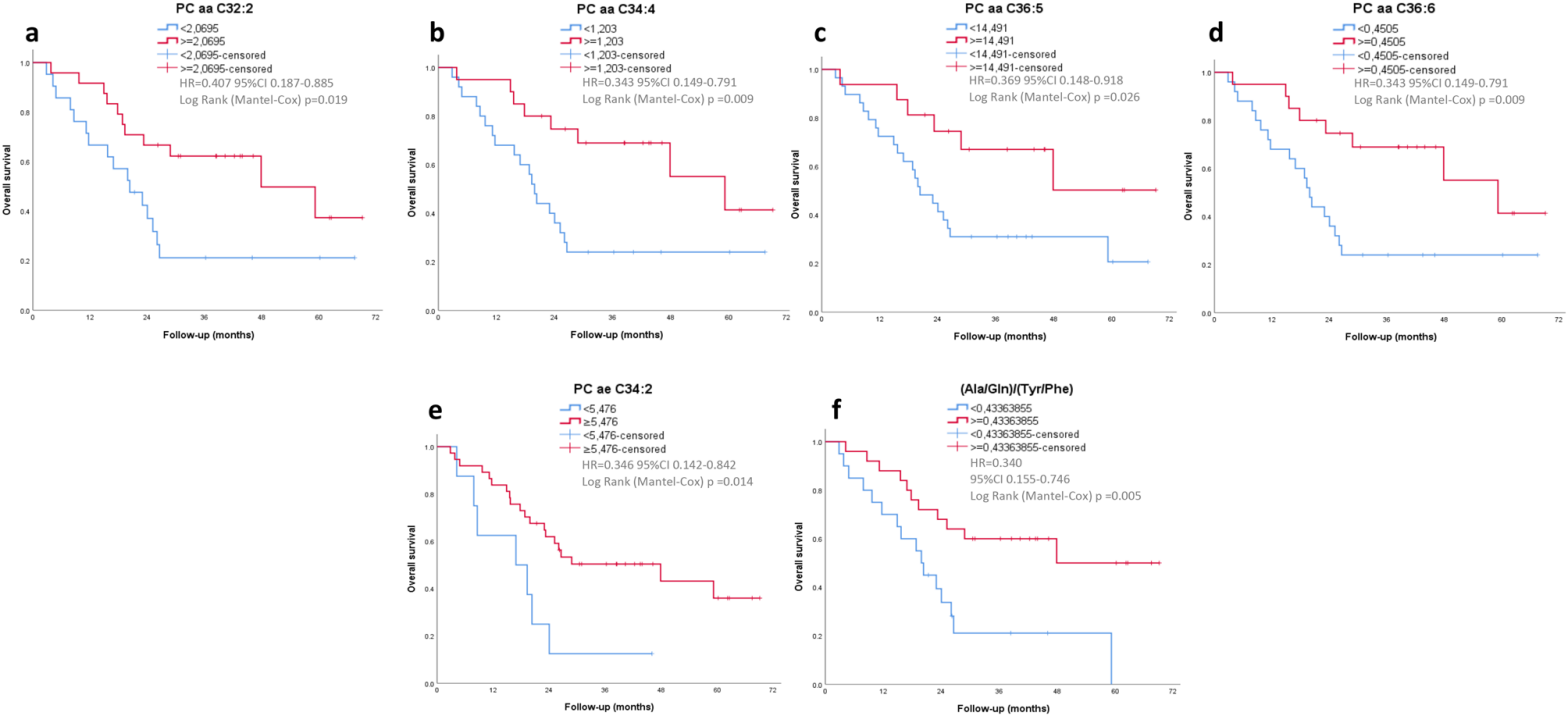
Prognostic significance of plasma metabolites and metabolite ratios. Kaplan–Meier survival curves for HNSCC patients stratified by seven plasma metabolites and metabolite ratios with a cut-off according to the Youden index: (**a**) PC aa C32:2; (**b**) PC aa C34:4; (**c**) PC aa C36:5; (**d**) PC aa C36:6; (**e**) PC ae 34:2; (**f**) (Ala/Gln)/(Tyr/Phe). HR = Hazard Ratio; CI = 95% Confidence Interval.

The eight most promising variables were thus selected for Kaplan–Meier curve analysis. The results demonstrated that reduced levels of six variables, including amino acids and phosphatidylcholines with fatty acid chains totalling 32–36 carbons [(Ala/Gln)/(Tyr/Phe) ratio and PC aa 32.2, PC aa 34:4, PC aa 36:5, PC aa 36:6, PC ae 34:2], were associated with an unfavorable prognosis (*p* < 0.05) (Fig. 10). These results reinforce the role of glutamine and phosphatidylcholines in tumor progression.

Our analysis of survival regarding to plasma metabolomics data of OSCC is believed to be the first using a large number of variables. The results are promising and encouraging for further studies and efforts in developing a metabolite panel for predictive screening.

## Conclusion

The number of protein-coding genes is estimated to be approximately 20,000, and a priori, the number of proteins is approximately the same. However, this number may increase to several million due to alternative splicing, posttranslational modifications, and variation caused by somatic recombination or polymorphisms. In contrast, the metabolome is smaller. The Human Metabolome Database/HMDB includes approximately 220 thousand metabolite entries. In addition, the metabolome is more precisely characterized, and specific metabolites may be used for prognostic and treatment monitoring and as therapeutic targets. Despite these attractive features, there is diversity and heterogeneity in cancer patients, which creates a need for the enhancement of our knowledge of the cellular metabolism of particular tumours, including oral carcinomas.

The present study observed decreased levels of specific amino acids and phosphatidylcholines associated with reduced survival, reinforcing their role in tumor progression and standing out as a potential member of a metabolite panel for predictive screening. In addition, we identified a distinct plasma metabolic OSCC profile suggestive of abnormal ketogenesis, lipogenesis and energy metabolism, which is more evident in advanced stages of the disease and may contribute to inflammation, inhibit immune response and promote tumour growth. Four nonexclusive and broad views may explain the data: differential synthesis, uptake, release, and degradation of metabolites. The best interpretation that assimilates these views is the cross talk between tumour cells and associated/normal cells, both nearby in the microenvironment or in more distant anatomical sites, that are connected by biofluids and their signalling molecules and vesicles. Therefore, it is important that additional population samples and metabolomic studies be conducted to unravel the details of these molecular processes, which may further increase the power for detecting new biomarkers and for developing novel strategies for OSCC treatment and prognostic approaches.

## Methods

### Study subjects and sample/data collection

Plasma samples from 72 OSCC patients and 61 controls were collected by four institutions: the Faculty of Medicine (University of São Paulo), Heliopolis Hospital, Arnaldo Vieira de Carvalho Cancer Institute, and Barretos Cancer Hospital. These institutions and their investigators are members of the Head and Neck Genome Project (GENCAPO), a collaborative consortium of research groups from hospitals and universities in São Paulo State, Brazil, whose aim is to develop clinical, genetic and epidemiological analyses of head and neck squamous cell carcinomas. The first three institutions are located in the same urban area—São Paulo city—with 12 million people, but 50 million people are concentrated in the metropolitan area. The fourth institution is located in Barretos, a smaller city 430 km from São Paulo, with a metropolitan area that includes 16 counties and approximately 400 thousand people. The São Paulo population has a mixed background, with descendants of different Brazilian regions and nationalities, whereas the Barretos population, both urban and rural, has a more homogeneous background.

The inclusion criteria comprised OSCC patients who received no preoperative radiation or chemotherapy and were diagnosed with clinically and histologically confirmed primary squamous cell carcinoma of the oral cavity (subsites C02 = other and unspecified parts of tongue; C03 = gum; C04 = floor of mouth; C06 = other and unspecified parts of mouth; according to the International Classification of Diseases, 10th revision—ICD-10), classified by the TNM system as lymph node-positive (N+ or N1-3) or lymph node-negative (N0) tumours in the T1-3 categories^133^. Control plasma samples were obtained from age- and sex-matched volunteers recruited among in- and outpatients of the same hospitals in which patients were treated, with admission diagnosis not related to tobacco smoking or alcohol consumption. Clinical information on the participants is presented in Supplementary Tables 2 and 3.

A total of 10 mL of venous blood was withdrawn before or 2 h after breakfast (between 8:00 and 10:00 AM), according to a detailed standard operating procedure. The blood was collected into EDTA tubes and centrifuged for 30 min at 2000 g within 1 h. Plasma samples were then aliquoted, evaluated for haemolysis by spectrophotometric analysis at 414-nm wavelength and stored at − 80 °C until use.

Body mass index (BMI) was measured by medical staff and/or self-reported. Patients and controls with BMI < 18.50 were classified as underweight, those with BMI of 18.50–24.99 as ideal weight, and those with BMI > 25.00 as overweight^134^.

The study protocol and the informed consent were approved by the Committees on Ethics in Research of Clinics Hospital—Faculty of Medicine (University of São Paulo), Heliopolis Hospital, Arnaldo Vieira de Carvalho Cancer Institute, Barretos Cancer Hospital, and by the National Committee on Ethics in Research/CONEP (reference number 1763/05, 18/05/2005). All experiments were performed in accordance with the 1964 Declaration of Helsinki and amendments, and all patients provided their written consent to participate in the study after being informed about the research purposes.

### ^1^H Nuclear magnetic resonance (NMR) spectroscopy

Plasma samples (1 mL) were subjected to filtration on Amicon Ultra Centrifugal Filters* (*Ultracel—3 K Membrane Regenerated Cellulose 3000 MWCO) (Millipore, Billerica, MA, USA). After centrifugation (14,000 g, 4 °C, 30 min), 480 µL (80% v/v), of the filtrate was mixed with 60 µL (10% v/v) of a phosphate solution (1 M sodium phosphate, 30 mM sodium azide, 100 mM imidazole, 5 mM trimethylsilyl propanoic acid) and 60 µL (10% v/v) of deuterium oxide (D_2_O). Trimethylsilyl propanoic acid (TSP) was added to the sample to provide an internal reference for the chemical shifts (0 ppm).

*NMR data acquisition.* The mixtures (600 µL) were transferred into 5-mm NMR capillary tubes and kept on ice until the measurement. Measurements were made in two different NMR facilities, initially in the National Biosciences Laboratory in Campinas, SP, and later in the facility at the University of São Paulo State in São José do Rio Preto, SP, after the facility was commissioned. The experiments were conducted with the same relevant parameters in both spectrometers, and the source of the data and robustness of analises are complementary steps in the assertion of the minimization of inherent biases.

^1^H NMR spectra were acquired on two spectrometers: on a Varian Inova 500 MHz spectrometer (Agilent Technologies, Inc., Santa Clara, CA) or on a Bruker Avance-III 600 NMR (Bruker BioSpin Billerica, MA, USA).

*Inova equipment*. ^1^H NMR spectra were acquired on two spectrometers. Spectra of 20 patient samples were measured on a Varian Inova 500 MHz spectrometer (Agilent Technologies, Inc., Santa Clara, CA) equipped with a 5-mm triple-gradient probe, pulsed field gradient in Z axis, temperature at 20 °C. Each sample was measured with the excitation sculping method ZGESGP using the parameters: 128 scans, 8 dummy scans, spectral width of 16 ppm centered at 4.695 ppm, acquisition time of 1.664 s.

*Bruker equipment*. Spectra of the other 27 patient and 49 control samples were measured on a Bruker Avance-III 600 MHz NMR (Bruker Germany) equipped with a 5-mm triple-gradient cryoprobe, pulsed field gradient in Z axis, temperature at 20 °C. Each sample was measured with the excitation sculping method ZGESGP using the parameters: 128 scans, 8 dummy scans, spectral width of 16 ppm centered at 4.695 ppm, acquisition time of 1.664 s.

All spectra were manually phased and baseline-corrected. The chemical shifts were referenced to the internal standard, TSP. Identification and quantification of metabolites in spectra was performed using the Chenomx NMR Suite v7.7 (Chenomx Inc., Calgary, Canada).

### Mass spectrometry (MS) analysis

The Absolute*IDQ*® p180 Kit (BIOCRATES Life Sciences AG, Innsbruck, Austria) was used for the MS analysis. The platform quantifies up to 188 metabolites: 21 amino acids; 21 biogenic amines; 1 hexose (sum of hexoses); 40 acylcarnitines (Cx:y), hydroxylacylcarnitines [C (OH) x:y] and dicarboxylacylcarnitines (Cx:y-DC); 90 glycerophospholipids (76 phosphatidylcholines/PC and 14 lyso-phosphatidylcholines); and 15 sphingolipids/sphingomyelins (SMx:y) and sphingomyelin derivatives [SM (OH) x:y]. Amino acids were abbreviated according to standard three letter nomenclature, and acylcarnitines were denominated according to the fatty acid that was bound (e.g., C2 = acetyl-L-carnitine). Glycerophospholipids were differentiated with respect to ester (“a”) and ether (“e”) bonds, with two letters [“aa” (= diacyl) and “ae” (= acyl-alkyl)] indicating that two glycerol positions are bound to a fatty acid residue and one letter [“a” (= acyl)] indicating the presence of a single fatty acid residue. Lipid side chains were abbreviated as Cx:y, where x means the number of carbons in the side chain and y the number of double bonds.

The assay is based on PITC (phenylisothiocyanate) derivatization in the presence of internal standards followed by flow injection analysis-tandem MS/FIA-MS/MS (acylcarnitines, lipids, and hexoses) and liquid chromatography–MS/LC‒MS/MS (amino acids and biogenic amines) using a SCIEX 4000 QTrap® mass spectrometer (SCIEX, Darmstadt, Germany) or a Waters XEVO TQMS® mass spectrometer (Waters, Vienna, Austria) with electrospray ionization. The experimental metabolomics measurement technique is described in detail in patent US 2007/0004044 (S. L. Ramsay, W. M. Stoegg, K. M. Weinberger, A. Graber, and W. Guggenbichler, “Apparatus and method for analyzing a metabolite profile,” EP 1875401 A211-Jan-2007). Sample preparation and metabolomic analyses were performed at Biocrates Life Sciences AG (Innsbruck, Austria).

To increase the accuracy of the analysis, the data were filtered by excluding metabolites that showed concentration values below the limit of detection (LOD) or were not detected (“no peak” detected).

### Statistical analysis

Statistical analysis was performed using MetaboAnalyst 5.0 software (http://www.metaboanalyst.ca/)^135^. Metabolite concentration values (in μM) were normalized by the median, log transformed and autoscaled (mean-centred and divided by the standard deviation of each variable), and the t test (*p* < 0.05; FDR-adjusted), volcano plot (fold change threshold 2.0; *p* value threshold 0.05) and partial least squares discriminant analysis (PLS-DA) were performed on the distribution of these metabolites in different groups of subjects. Metabolite hierarchical clustering was performed using Ward’s method. The variables were also ranked according to their variable importance in projection (VIP) value, a measure of its contribution in the PLS regression. VIP scores greater than 1 were considered important for the PLS prediction. The PLS-DA models were validated using cross-validation and permutation tests (number of permutations = 1000).

Multivariate receiver operating characteristic (ROC) curves were generated by Monte-Carlo cross validation (MCCV) using balanced subsampling. In each MCCV, two-thirds of the samples were used to evaluate the feature importance. The top important features were used to build classification models that were validated on the remaining one-third of the samples. The procedure was repeated multiple times to calculate the performance and confidence interval of each model. ROC curves for individual biomarkers were also generated and ranked based on the area under the curve (AUC), t-statistics or log2 fold change (FC).

Metabolic pathway enrichment and topology analyses were also carried out using the important features selected by t tests (*p* < 0.05) and the MetaboAnalyst platform, which provides high-quality metabolic pathways as the backend knowledgebase. The statistical *p* values from enrichment analysis were adjusted for multiple testings: the raw original *p* value was calculated from the enrichment analysis; the Holm *p* value and the FDR were adjusted by the Holm‒Bonferroni method and the false discovery rate, respectively; and the impact value was calculated from pathway topology analysis. The KEGG (Kyoto Encyclopedia of Genes and Genomes) database was used as the pathway library for database selection. Pathways with a number of hits ≥ 2, Holm and/or impact *p* values < 0.05 or = 1.0, respectively, were considered influential pathways.

For prognostic analyses, overall survival was defined as the number of months between the date of the histological diagnosis and the date of death or last follow-up for surviving patients of set A. The univariate Cox regression (continuous variables) was initially applied to calculate the hazard ratio (HR) values and the 95% confidence intervals (CIs). Variables with *p* values ≤ 0.10 were potentially considered to have prognostic significance. The discriminatory accuracy and the optimal cut-off threshold of a variable were obtained by the area under the ROC curve (AUC) and by the Youden Index. Kaplan–Meier curves were generated and compared using log-rank test, and the variables with *p* values < 0.05 were considered to have prognostic significance. The analyses were conducted on the Statistical Package for the Social Sciences (SPSS, version 18.0 for Windows; IBM, Corp., Armonk, NY) software.

## Supplementary Information

Supplementary Tables.

Supplementary Figures.

## Data Availability

All data generated or analysed during this study are included in the article and its supplementary information files.
