# Supplementary material for: Plasma metabolomics of oral squamous cell carcinomas based on NMR and MS approaches provides biomarker identification and survival prediction
Source: Sci Rep. 2023 May 26;13:8588. doi: 10.1038/s41598-023-34808-2 (PMC10220089; doi:10.1038/s41598-023-34808-2)
Supplement: Supplementary file 2 — Supplementary Figures. [file 41598_2023_34808_MOESM2_ESM.pptx]

## Slide 1
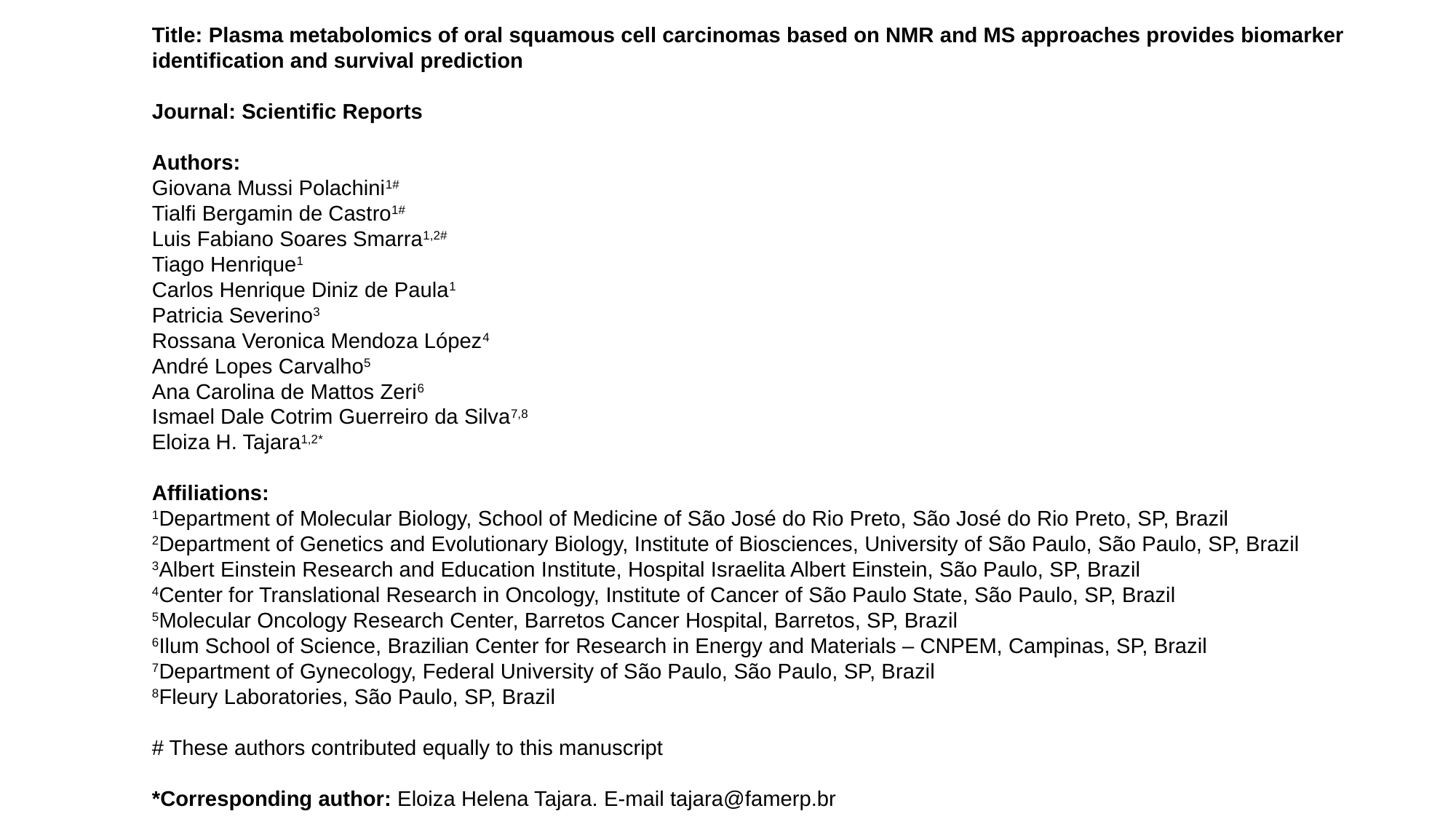

Title: Plasma metabolomics of oral squamous cell carcinomas based on NMR and MS approaches provides biomarker
identification and survival prediction
Journal: Scientific Reports
Authors:
Giovana Mussi Polachini1#
Tialfi Bergamin de Castro1#
Luis Fabiano Soares Smarra1,2#
Tiago Henrique1
Carlos Henrique Diniz de Paula1
Patricia Severino3
Rossana Veronica Mendoza López4
André Lopes Carvalho5
Ana Carolina de Mattos Zeri6
Ismael Dale Cotrim Guerreiro da Silva7,8
Eloiza H. Tajara1,2*
Affiliations:
1Department of Molecular Biology, School of Medicine of São José do Rio Preto, São José do Rio Preto, SP, Brazil
2Department of Genetics and Evolutionary Biology, Institute of Biosciences, University of São Paulo, São Paulo, SP, Brazil
3Albert Einstein Research and Education Institute, Hospital Israelita Albert Einstein, São Paulo, SP, Brazil
4Center for Translational Research in Oncology, Institute of Cancer of São Paulo State, São Paulo, SP, Brazil
5Molecular Oncology Research Center, Barretos Cancer Hospital, Barretos, SP, Brazil
6Ilum School of Science, Brazilian Center for Research in Energy and Materials – CNPEM, Campinas, SP, Brazil
7Department of Gynecology, Federal University of São Paulo, São Paulo, SP, Brazil
8Fleury Laboratories, São Paulo, SP, Brazil
# These authors contributed equally to this manuscript
*Corresponding author: Eloiza Helena Tajara. E-mail tajara@famerp.br

## Slide 2
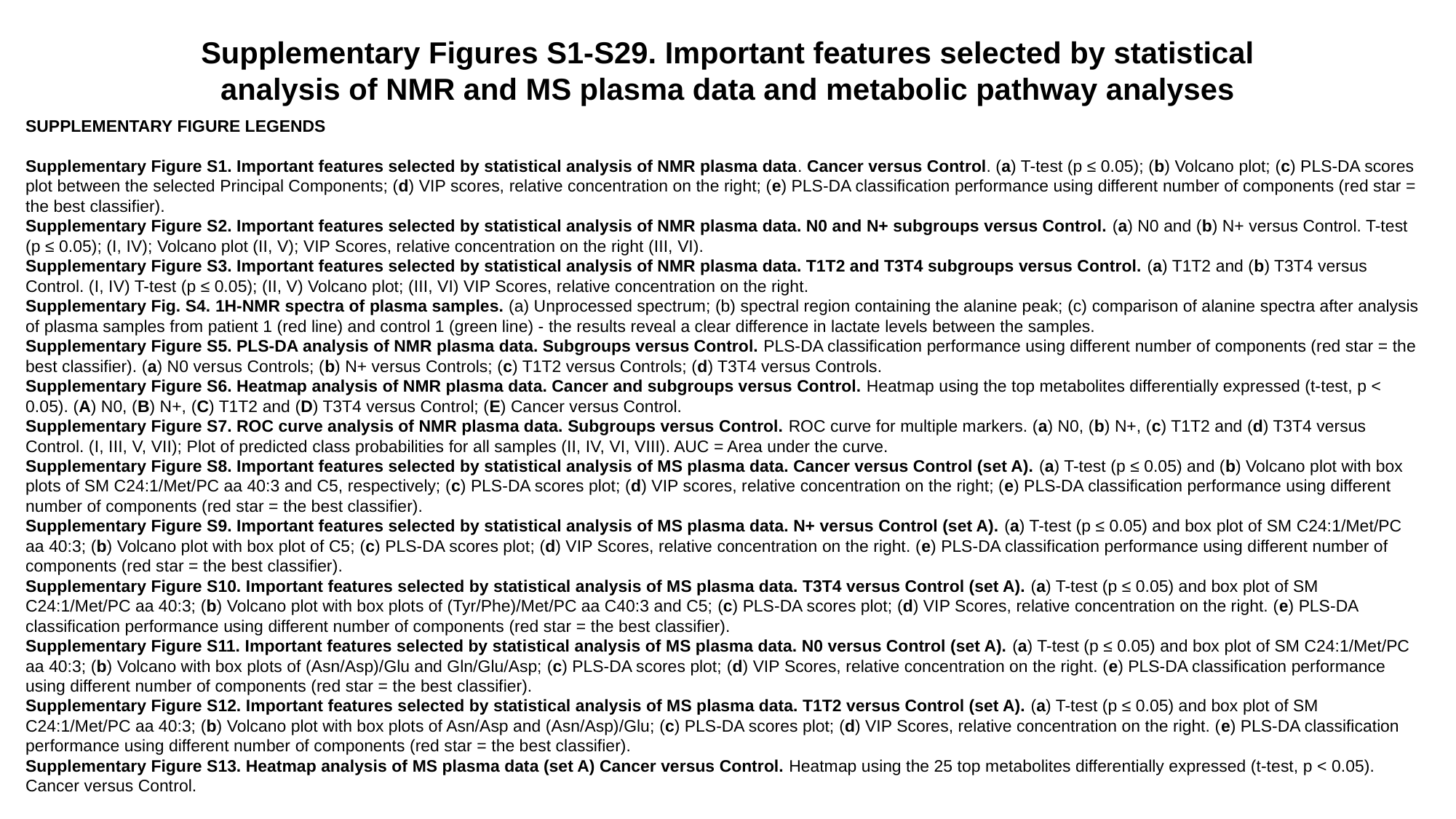

Supplementary Figures S1-S29. Important features selected by statistical analysis of NMR and MS plasma data and metabolic pathway analyses
SUPPLEMENTARY FIGURE LEGENDS
Supplementary Figure S1. Important features selected by statistical analysis of NMR plasma data. Cancer versus Control. (a) T-test (p ≤ 0.05); (b) Volcano plot; (c) PLS-DA scores plot between the selected Principal Components; (d) VIP scores, relative concentration on the right; (e) PLS-DA classification performance using different number of components (red star = the best classifier).
Supplementary Figure S2. Important features selected by statistical analysis of NMR plasma data. N0 and N+ subgroups versus Control. (a) N0 and (b) N+ versus Control. T-test (p ≤ 0.05); (I, IV); Volcano plot (II, V); VIP Scores, relative concentration on the right (III, VI).
Supplementary Figure S3. Important features selected by statistical analysis of NMR plasma data. T1T2 and T3T4 subgroups versus Control. (a) T1T2 and (b) T3T4 versus Control. (I, IV) T-test (p ≤ 0.05); (II, V) Volcano plot; (III, VI) VIP Scores, relative concentration on the right.
Supplementary Fig. S4. 1H-NMR spectra of plasma samples. (a) Unprocessed spectrum; (b) spectral region containing the alanine peak; (c) comparison of alanine spectra after analysis of plasma samples from patient 1 (red line) and control 1 (green line) - the results reveal a clear difference in lactate levels between the samples.
Supplementary Figure S5. PLS-DA analysis of NMR plasma data. Subgroups versus Control. PLS-DA classification performance using different number of components (red star = the best classifier). (a) N0 versus Controls; (b) N+ versus Controls; (c) T1T2 versus Controls; (d) T3T4 versus Controls.
Supplementary Figure S6. Heatmap analysis of NMR plasma data. Cancer and subgroups versus Control. Heatmap using the top metabolites differentially expressed (t-test, p < 0.05). (A) N0, (B) N+, (C) T1T2 and (D) T3T4 versus Control; (E) Cancer versus Control.
Supplementary Figure S7. ROC curve analysis of NMR plasma data. Subgroups versus Control. ROC curve for multiple markers. (a) N0, (b) N+, (c) T1T2 and (d) T3T4 versus Control. (I, III, V, VII); Plot of predicted class probabilities for all samples (II, IV, VI, VIII). AUC = Area under the curve.
Supplementary Figure S8. Important features selected by statistical analysis of MS plasma data. Cancer versus Control (set A). (a) T-test (p ≤ 0.05) and (b) Volcano plot with box plots of SM C24:1/Met/PC aa 40:3 and C5, respectively; (c) PLS-DA scores plot; (d) VIP scores, relative concentration on the right; (e) PLS-DA classification performance using different number of components (red star = the best classifier).
Supplementary Figure S9. Important features selected by statistical analysis of MS plasma data. N+ versus Control (set A). (a) T-test (p ≤ 0.05) and box plot of SM C24:1/Met/PC aa 40:3; (b) Volcano plot with box plot of C5; (c) PLS-DA scores plot; (d) VIP Scores, relative concentration on the right. (e) PLS-DA classification performance using different number of components (red star = the best classifier).
Supplementary Figure S10. Important features selected by statistical analysis of MS plasma data. T3T4 versus Control (set A). (a) T-test (p ≤ 0.05) and box plot of SM C24:1/Met/PC aa 40:3; (b) Volcano plot with box plots of (Tyr/Phe)/Met/PC aa C40:3 and C5; (c) PLS-DA scores plot; (d) VIP Scores, relative concentration on the right. (e) PLS-DA classification performance using different number of components (red star = the best classifier).
Supplementary Figure S11. Important features selected by statistical analysis of MS plasma data. N0 versus Control (set A). (a) T-test (p ≤ 0.05) and box plot of SM C24:1/Met/PC aa 40:3; (b) Volcano with box plots of (Asn/Asp)/Glu and Gln/Glu/Asp; (c) PLS-DA scores plot; (d) VIP Scores, relative concentration on the right. (e) PLS-DA classification performance using different number of components (red star = the best classifier).
Supplementary Figure S12. Important features selected by statistical analysis of MS plasma data. T1T2 versus Control (set A). (a) T-test (p ≤ 0.05) and box plot of SM C24:1/Met/PC aa 40:3; (b) Volcano plot with box plots of Asn/Asp and (Asn/Asp)/Glu; (c) PLS-DA scores plot; (d) VIP Scores, relative concentration on the right. (e) PLS-DA classification performance using different number of components (red star = the best classifier).
Supplementary Figure S13. Heatmap analysis of MS plasma data (set A) Cancer versus Control. Heatmap using the 25 top metabolites differentially expressed (t-test, p < 0.05). Cancer versus Control.

## Slide 3
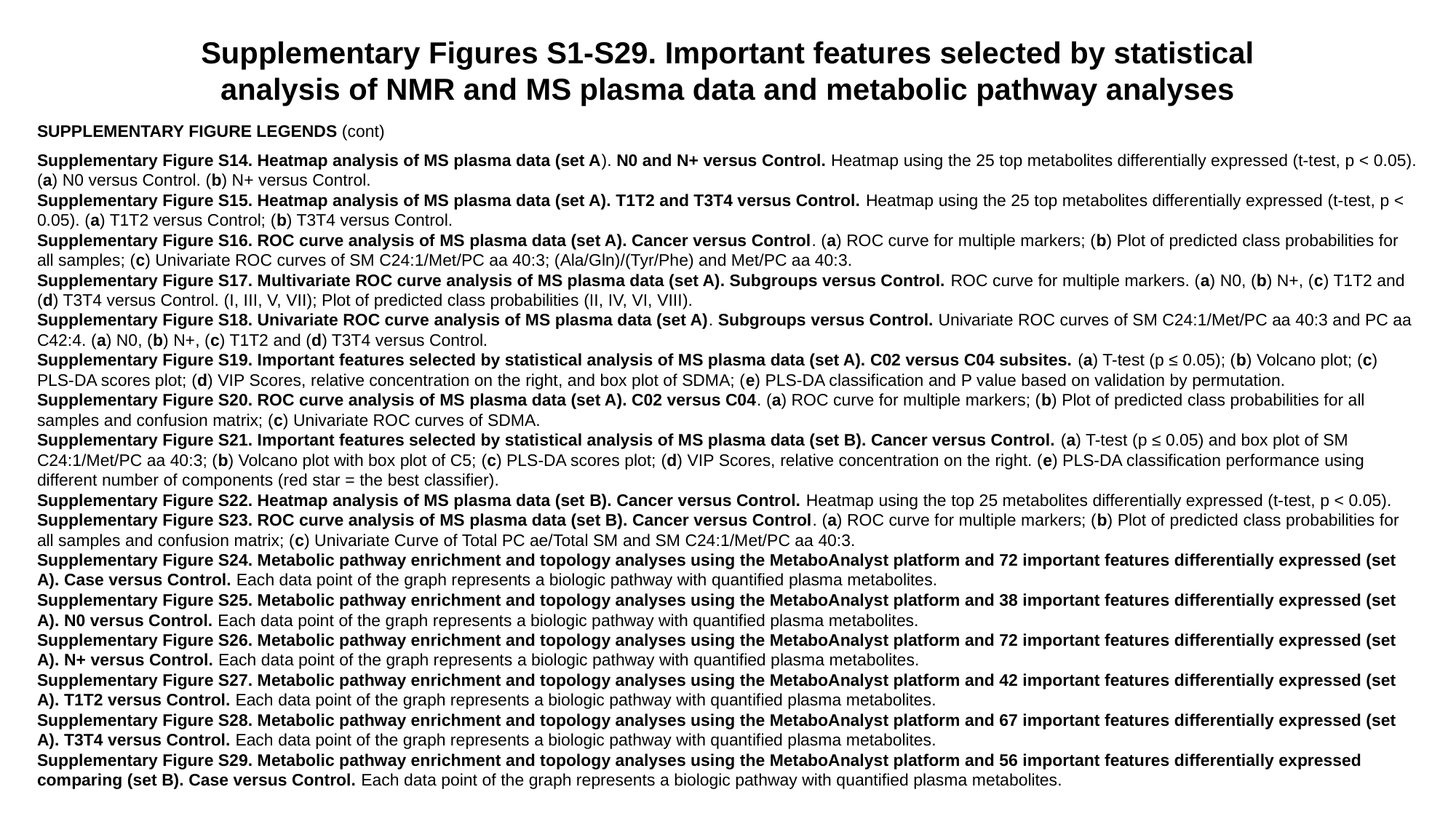

Supplementary Figures S1-S29. Important features selected by statistical analysis of NMR and MS plasma data and metabolic pathway analyses
SUPPLEMENTARY FIGURE LEGENDS (cont)
Supplementary Figure S14. Heatmap analysis of MS plasma data (set A). N0 and N+ versus Control. Heatmap using the 25 top metabolites differentially expressed (t-test, p < 0.05). (a) N0 versus Control. (b) N+ versus Control.
Supplementary Figure S15. Heatmap analysis of MS plasma data (set A). T1T2 and T3T4 versus Control. Heatmap using the 25 top metabolites differentially expressed (t-test, p < 0.05). (a) T1T2 versus Control; (b) T3T4 versus Control.
Supplementary Figure S16. ROC curve analysis of MS plasma data (set A). Cancer versus Control. (a) ROC curve for multiple markers; (b) Plot of predicted class probabilities for all samples; (c) Univariate ROC curves of SM C24:1/Met/PC aa 40:3; (Ala/Gln)/(Tyr/Phe) and Met/PC aa 40:3.
Supplementary Figure S17. Multivariate ROC curve analysis of MS plasma data (set A). Subgroups versus Control. ROC curve for multiple markers. (a) N0, (b) N+, (c) T1T2 and (d) T3T4 versus Control. (I, III, V, VII); Plot of predicted class probabilities (II, IV, VI, VIII).
Supplementary Figure S18. Univariate ROC curve analysis of MS plasma data (set A). Subgroups versus Control. Univariate ROC curves of SM C24:1/Met/PC aa 40:3 and PC aa C42:4. (a) N0, (b) N+, (c) T1T2 and (d) T3T4 versus Control.
Supplementary Figure S19. Important features selected by statistical analysis of MS plasma data (set A). C02 versus C04 subsites. (a) T-test (p ≤ 0.05); (b) Volcano plot; (c) PLS-DA scores plot; (d) VIP Scores, relative concentration on the right, and box plot of SDMA; (e) PLS-DA classification and P value based on validation by permutation.
Supplementary Figure S20. ROC curve analysis of MS plasma data (set A). C02 versus C04. (a) ROC curve for multiple markers; (b) Plot of predicted class probabilities for all samples and confusion matrix; (c) Univariate ROC curves of SDMA.
Supplementary Figure S21. Important features selected by statistical analysis of MS plasma data (set B). Cancer versus Control. (a) T-test (p ≤ 0.05) and box plot of SM C24:1/Met/PC aa 40:3; (b) Volcano plot with box plot of C5; (c) PLS-DA scores plot; (d) VIP Scores, relative concentration on the right. (e) PLS-DA classification performance using different number of components (red star = the best classifier).
Supplementary Figure S22. Heatmap analysis of MS plasma data (set B). Cancer versus Control. Heatmap using the top 25 metabolites differentially expressed (t-test, p < 0.05).
Supplementary Figure S23. ROC curve analysis of MS plasma data (set B). Cancer versus Control. (a) ROC curve for multiple markers; (b) Plot of predicted class probabilities for all samples and confusion matrix; (c) Univariate Curve of Total PC ae/Total SM and SM C24:1/Met/PC aa 40:3.
Supplementary Figure S24. Metabolic pathway enrichment and topology analyses using the MetaboAnalyst platform and 72 important features differentially expressed (set A). Case versus Control. Each data point of the graph represents a biologic pathway with quantified plasma metabolites.
Supplementary Figure S25. Metabolic pathway enrichment and topology analyses using the MetaboAnalyst platform and 38 important features differentially expressed (set A). N0 versus Control. Each data point of the graph represents a biologic pathway with quantified plasma metabolites.
Supplementary Figure S26. Metabolic pathway enrichment and topology analyses using the MetaboAnalyst platform and 72 important features differentially expressed (set A). N+ versus Control. Each data point of the graph represents a biologic pathway with quantified plasma metabolites.
Supplementary Figure S27. Metabolic pathway enrichment and topology analyses using the MetaboAnalyst platform and 42 important features differentially expressed (set A). T1T2 versus Control. Each data point of the graph represents a biologic pathway with quantified plasma metabolites.
Supplementary Figure S28. Metabolic pathway enrichment and topology analyses using the MetaboAnalyst platform and 67 important features differentially expressed (set A). T3T4 versus Control. Each data point of the graph represents a biologic pathway with quantified plasma metabolites.
Supplementary Figure S29. Metabolic pathway enrichment and topology analyses using the MetaboAnalyst platform and 56 important features differentially expressed comparing (set B). Case versus Control. Each data point of the graph represents a biologic pathway with quantified plasma metabolites.

## Slide 4
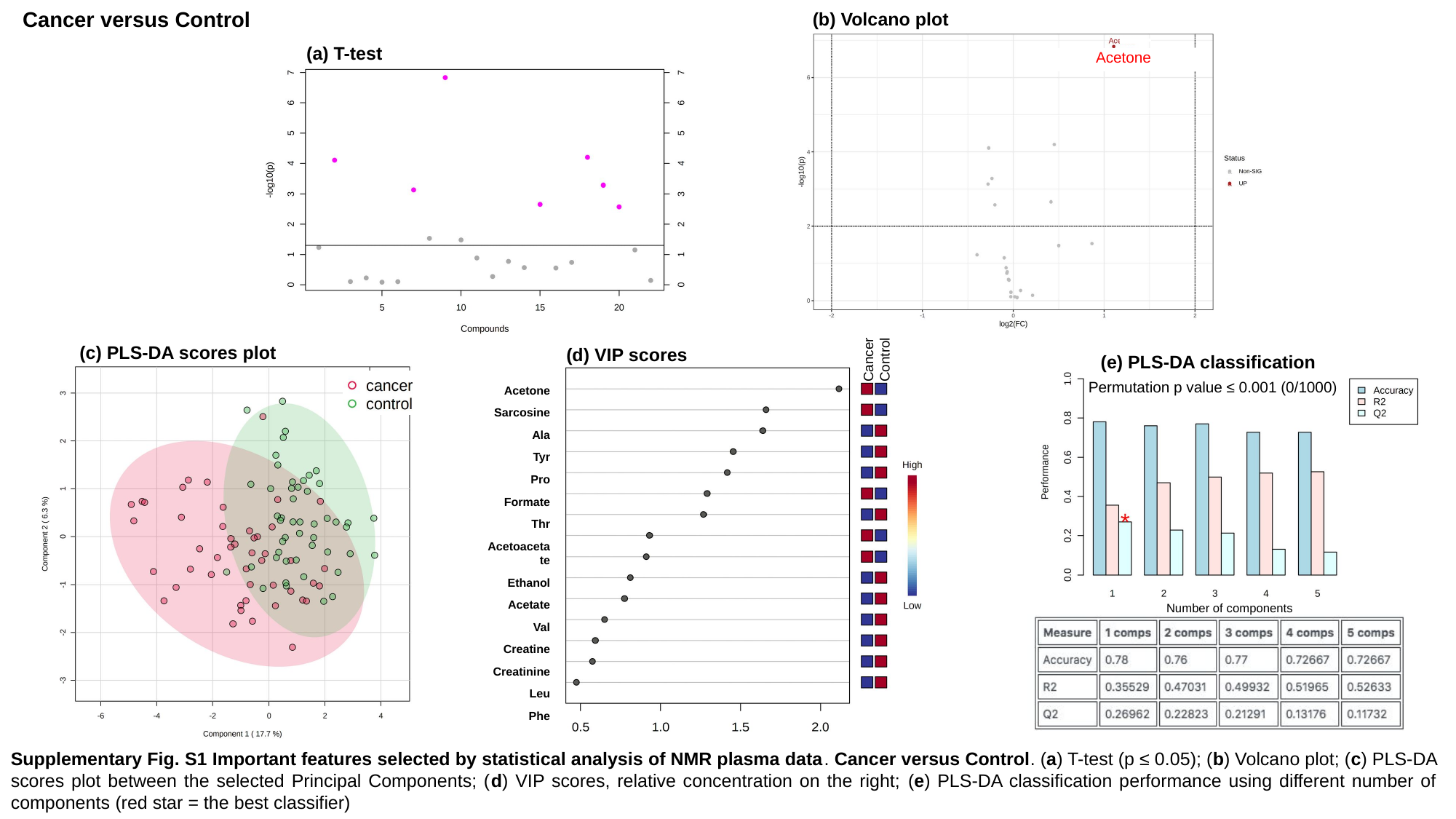

Cancer versus Control
(b) Volcano plot
(a) T-test
(e) PLS-DA classification
Permutation p value ≤ 0.001 (0/1000)
Number of components
Supplementary Fig. S1 Important features selected by statistical analysis of NMR plasma data. Cancer versus Control. (a) T-test (p ≤ 0.05); (b) Volcano plot; (c) PLS-DA scores plot between the selected Principal Components; (d) VIP scores, relative concentration on the right; (e) PLS-DA classification performance using different number of components (red star = the best classifier)
Acetone
Sarcosine
Ala
Tyr
Pro
Formate
Thr
Acetoacetate
Ethanol
Acetate
Val
Creatine
Creatinine
Leu
Phe
(c) PLS-DA scores plot
(d) VIP scores
Acetone
Cancer
Control

## Slide 5
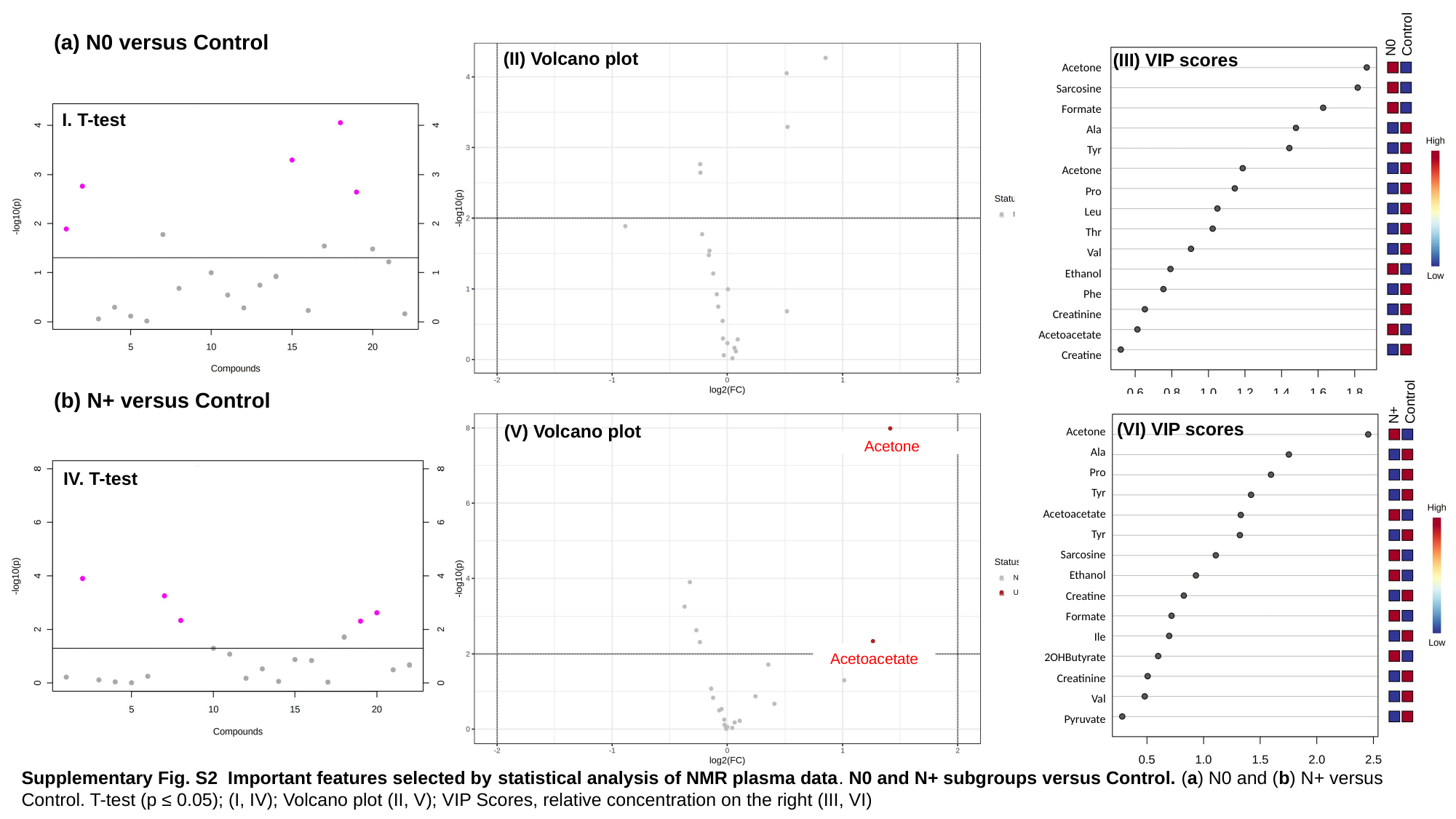

N0
Control
(a) N0 versus Control
(II) Volcano plot
(III) VIP scores
I. T-test
(b) N+ versus Control
(VI) VIP scores
(V) Volcano plot
(III) Volcano plot
IV. T-test
Supplementary Fig. S2 Important features selected by statistical analysis of NMR plasma data. N0 and N+ subgroups versus Control. (a) N0 and (b) N+ versus Control. T-test (p ≤ 0.05); (I, IV); Volcano plot (II, V); VIP Scores, relative concentration on the right (III, VI)
Acetone
Sarcosine
Formate
Ala
Tyr
Acetone
Pro
Leu
Thr
Val
Ethanol
Phe
Creatinine
Acetoacetate
Creatine
Acetone
Ala
Pro
Tyr
Acetoacetate
Tyr
Sarcosine
Ethanol
Creatine
Formate
Ile
2OHButyrate
Creatinine
Val
Pyruvate
0,00
N+
Control
0,00
Acetone
Acetoacetate

## Slide 6
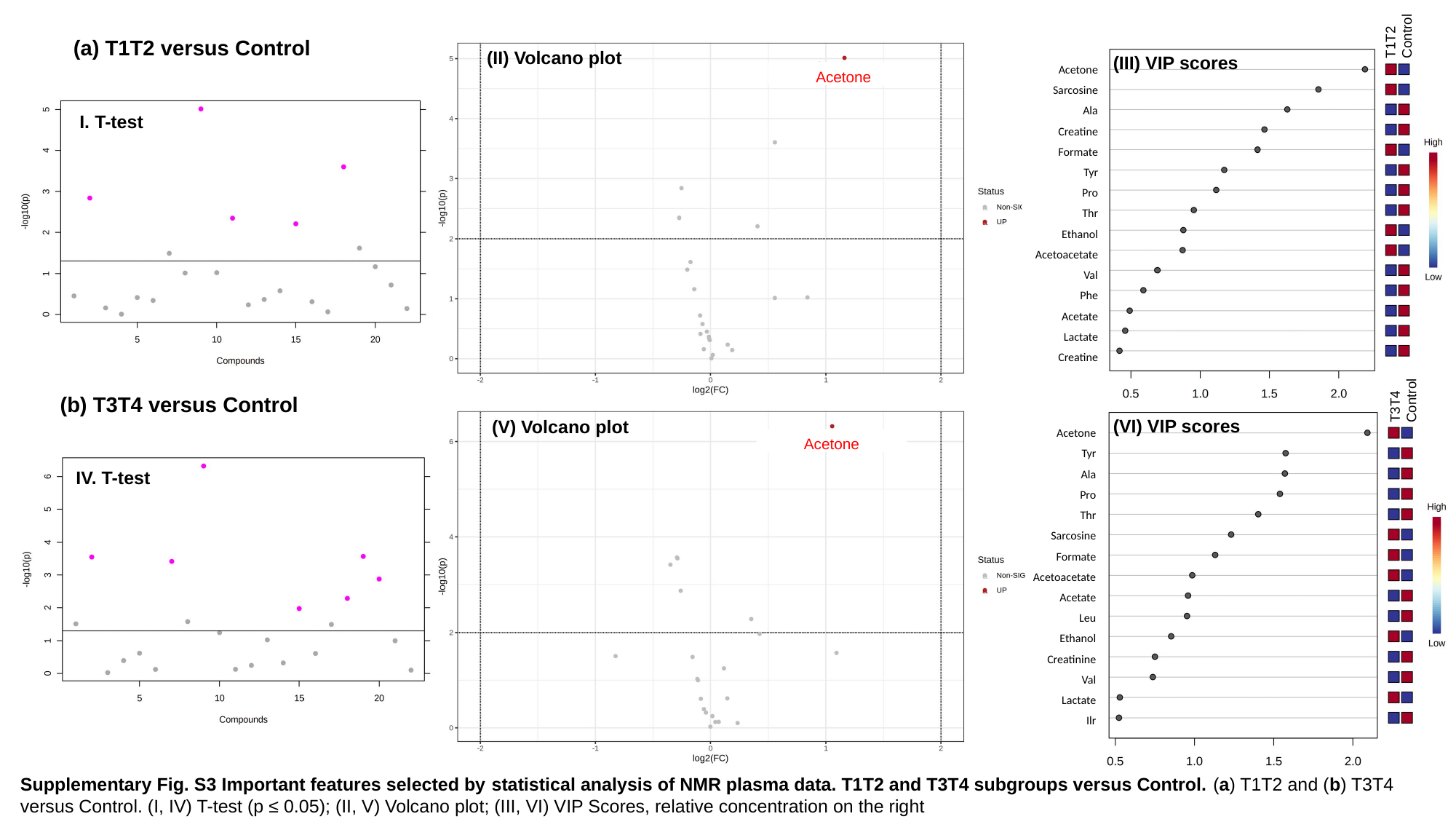

T1T2
Control
II. Volcano plot
V. Volcano plot
Acetone
Sarcosine
Ala
Creatine
Formate
Tyr
Pro
Thr
Ethanol
Acetoacetate
Val
Phe
Acetate
Lactate
Creatine
Acetone
Tyr
Ala
Pro
Thr
Sarcosine
Formate
Acetoacetate
Acetate
Leu
Ethanol
Creatinine
Val
Lactate
Ilr
Supplementary Fig. S3 Important features selected by statistical analysis of NMR plasma data. T1T2 and T3T4 subgroups versus Control. (a) T1T2 and (b) T3T4 versus Control. (I, IV) T-test (p ≤ 0.05); (II, V) Volcano plot; (III, VI) VIP Scores, relative concentration on the right
(a) T1T2 versus Control
(II) Volcano plot
(III) VIP scores
Acetone
I. T-test
T3T4
Control
(b) T3T4 versus Control
(VI) VIP scores
(V) Volcano plot
Acetone
IV. T-test

## Slide 7
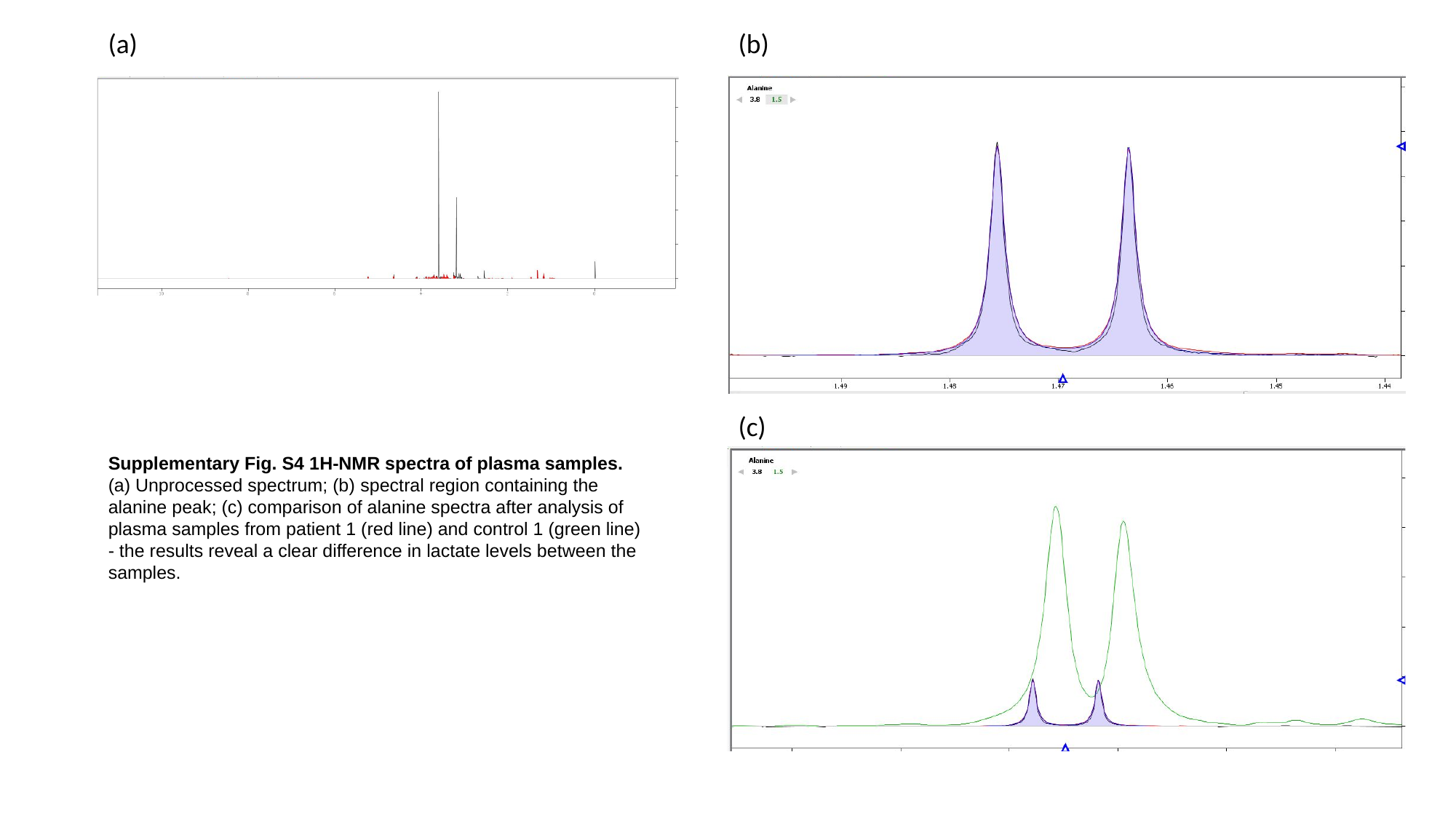

(a)
(b)
(c)
Supplementary Fig. S4 1H-NMR spectra of plasma samples. (a) Unprocessed spectrum; (b) spectral region containing the alanine peak; (c) comparison of alanine spectra after analysis of plasma samples from patient 1 (red line) and control 1 (green line) - the results reveal a clear difference in lactate levels between the samples.

## Slide 8
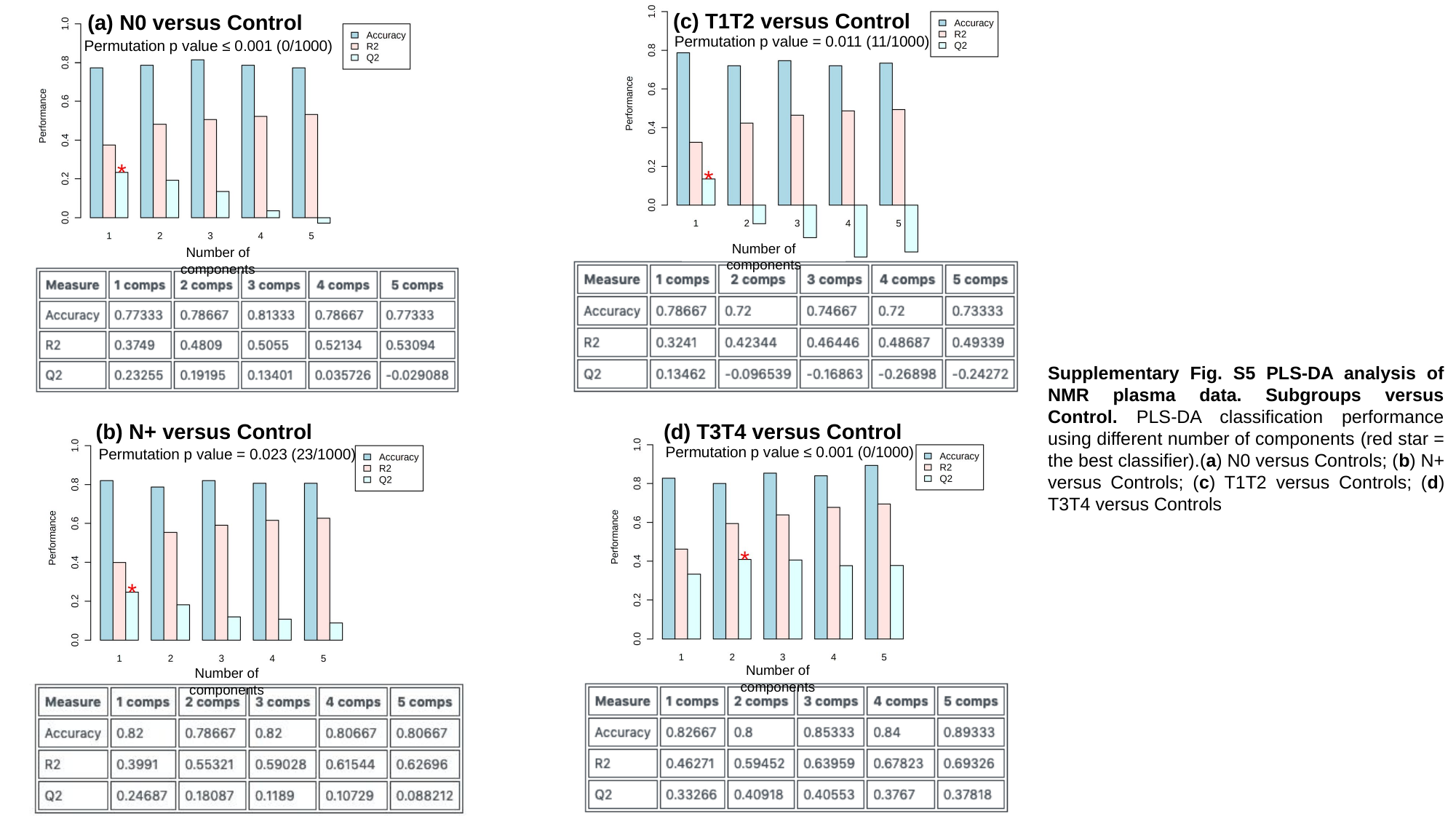

(c) T1T2 versus Control
Number of components
Permutation p value = 0.011 (11/1000)
(d) T3T4 versus Control
Permutation p value ≤ 0.001 (0/1000)
(a) N0 versus Control
Number of components
(b) N+ versus Control
Number of components
Permutation p value ≤ 0.001 (0/1000)
Permutation p value = 0.023 (23/1000)
Number of components
Supplementary Fig. S5 PLS-DA analysis of NMR plasma data. Subgroups versus Control. PLS-DA classification performance using different number of components (red star = the best classifier).(a) N0 versus Controls; (b) N+ versus Controls; (c) T1T2 versus Controls; (d) T3T4 versus Controls

## Slide 9
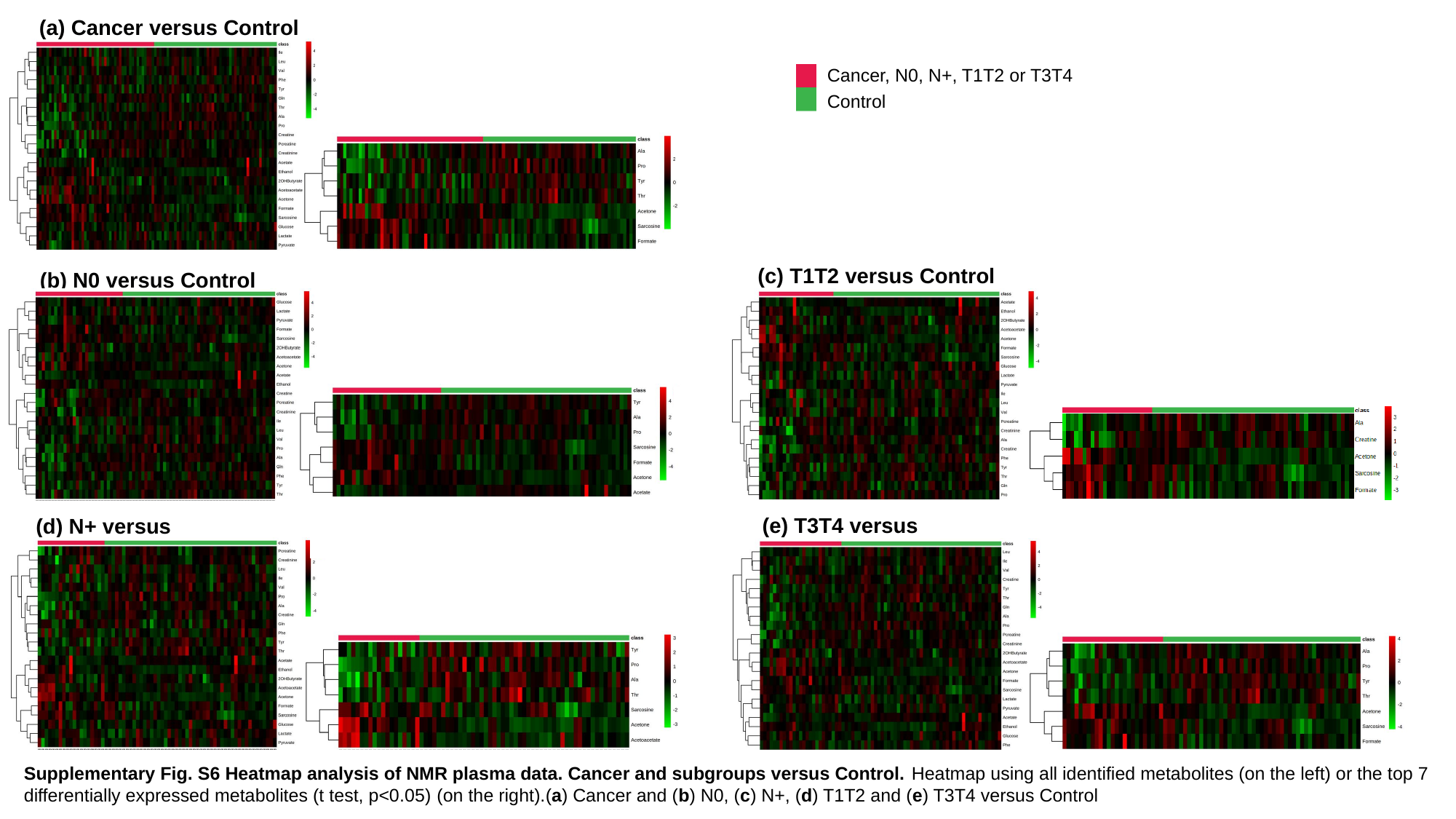

(a) Cancer versus Control
Cancer, N0, N+, T1T2 or T3T4
Control
(c) T1T2 versus Control
(b) N0 versus Control
(e) T3T4 versus Control
(d) N+ versus Control
Supplementary Fig. S6 Heatmap analysis of NMR plasma data. Cancer and subgroups versus Control. Heatmap using all identified metabolites (on the left) or the top 7 differentially expressed metabolites (t test, p<0.05) (on the right).(a) Cancer and (b) N0, (c) N+, (d) T1T2 and (e) T3T4 versus Control

## Slide 10
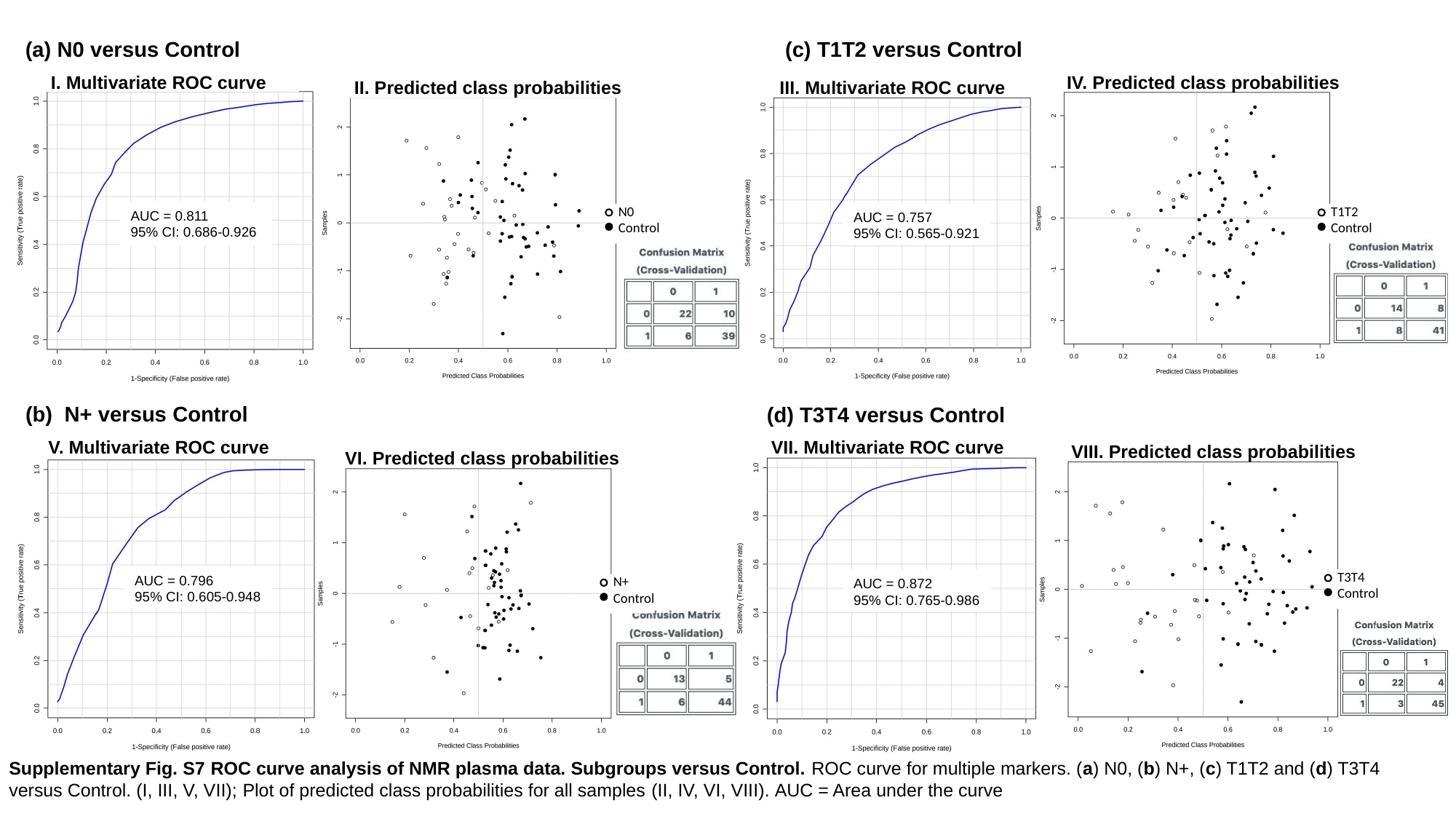

(a) N0 versus Control
(c) T1T2 versus Control
I. Multivariate ROC curve
IV. Predicted class probabilities
II. Predicted class probabilities
III. Multivariate ROC curve
AUC = 0.811
95% CI: 0.686-0.926
AUC = 0.757
95% CI: 0.565-0.921
(b) N+ versus Control
(d) T3T4 versus Control
VII. Multivariate ROC curve
V. Multivariate ROC curve
VIII. Predicted class probabilities
VI. Predicted class probabilities
AUC = 0.796
95% CI: 0.605-0.948
AUC = 0.872
95% CI: 0.765-0.986
Supplementary Fig. S7 ROC curve analysis of NMR plasma data. Subgroups versus Control. ROC curve for multiple markers. (a) N0, (b) N+, (c) T1T2 and (d) T3T4 versus Control. (I, III, V, VII); Plot of predicted class probabilities for all samples (II, IV, VI, VIII). AUC = Area under the curve
N0
Control
T1T2
Control
T3T4
Control
N+
Control

## Slide 11
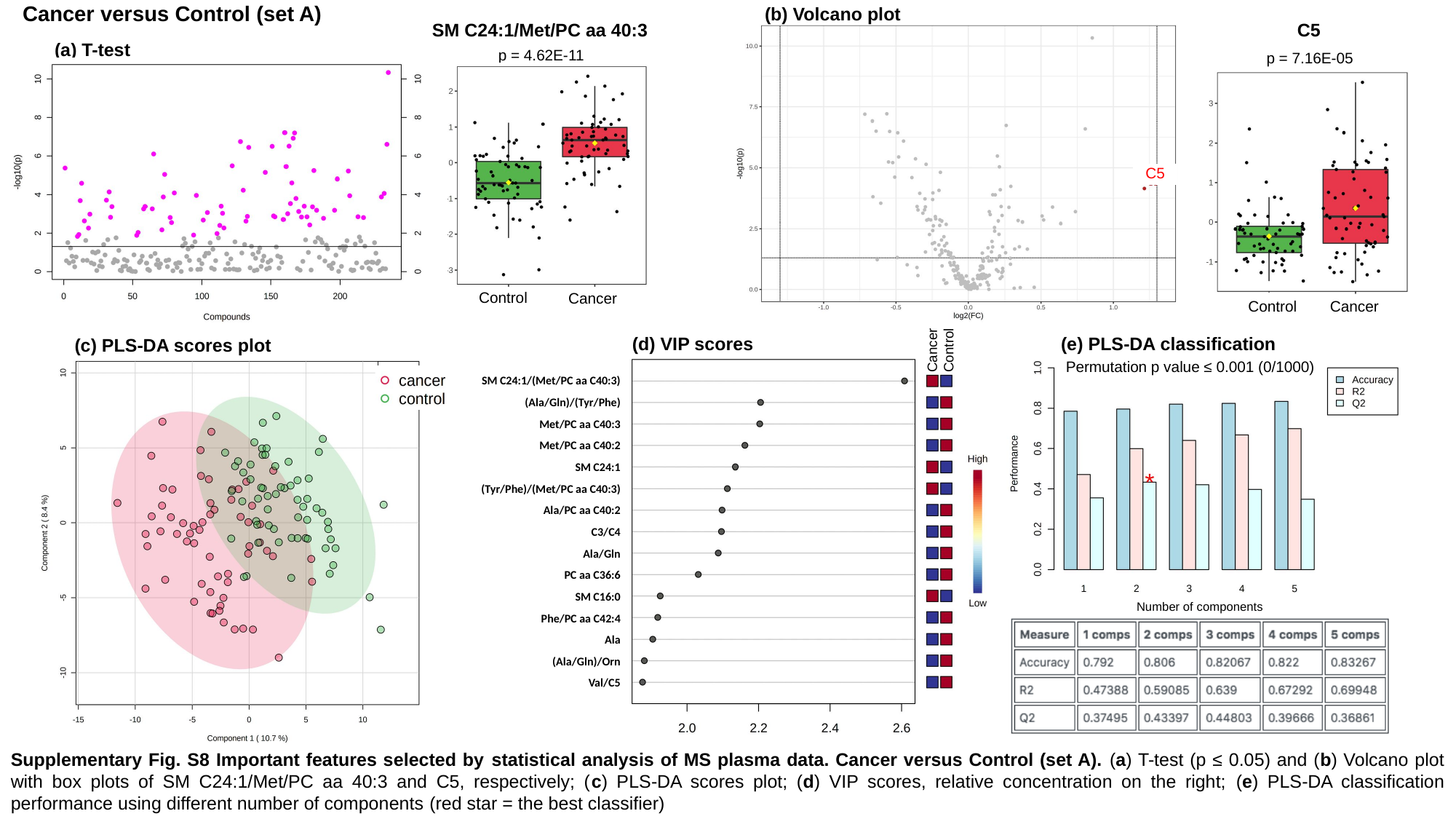

Cancer versus Control (set A)
(b) Volcano plot
SM C24:1/Met/PC aa 40:3
C5
(a) T-test
p = 4.62E-11
p = 7.16E-05
(d) VIP scores
(e) PLS-DA classification
Permutation p value ≤ 0.001 (0/1000)
SM C24:1/(Met/PC aa C40:3)
(Ala/Gln)/(Tyr/Phe)
Met/PC aa C40:3
Met/PC aa C40:2
SM C24:1
(Tyr/Phe)/(Met/PC aa C40:3)
Ala/PC aa C40:2
C3/C4
Ala/Gln
PC aa C36:6
SM C16:0
Phe/PC aa C42:4
Ala
(Ala/Gln)/Orn
Val/C5
Number of components
Supplementary Fig. S8 Important features selected by statistical analysis of MS plasma data. Cancer versus Control (set A). (a) T-test (p ≤ 0.05) and (b) Volcano plot with box plots of SM C24:1/Met/PC aa 40:3 and C5, respectively; (c) PLS-DA scores plot; (d) VIP scores, relative concentration on the right; (e) PLS-DA classification performance using different number of components (red star = the best classifier)
(c) PLS-DA scores plot
C5
Control
Cancer
Cancer
Cancer
Control
Control

## Slide 12
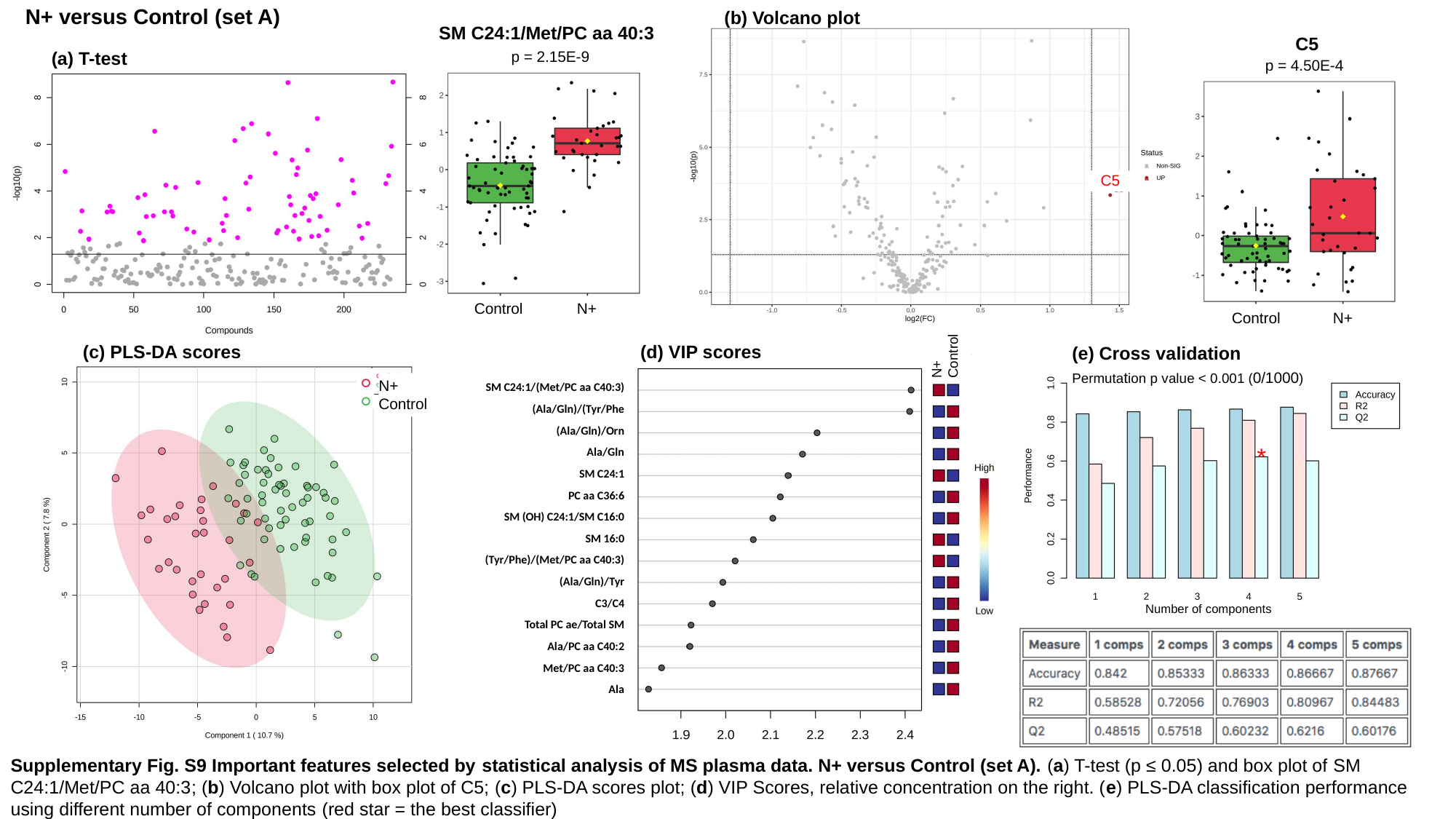

N+ versus Control (set A)
(b) Volcano plot
SM C24:1/Met/PC aa 40:3
SM C24:1/(Met/PC aa C40:3)
(Ala/Gln)/(Tyr/Phe
(Ala/Gln)/Orn
Ala/Gln
SM C24:1
PC aa C36:6
SM (OH) C24:1/SM C16:0
SM 16:0
(Tyr/Phe)/(Met/PC aa C40:3)
(Ala/Gln)/Tyr
C3/C4
Total PC ae/Total SM
Ala/PC aa C40:2
Met/PC aa C40:3
Ala
Number of components
Supplementary Fig. S9 Important features selected by statistical analysis of MS plasma data. N+ versus Control (set A). (a) T-test (p ≤ 0.05) and box plot of SM C24:1/Met/PC aa 40:3; (b) Volcano plot with box plot of C5; (c) PLS-DA scores plot; (d) VIP Scores, relative concentration on the right. (e) PLS-DA classification performance using different number of components (red star = the best classifier)
C5
(a) T-test
(d) VIP scores
(c) PLS-DA scores plot
(e) Cross validation
Permutation p value < 0.001 (0/1000)
p = 2.15E-9
p = 4.50E-4
C5
N+
Control
N+
Control
N+
Control
N+
Control

## Slide 13
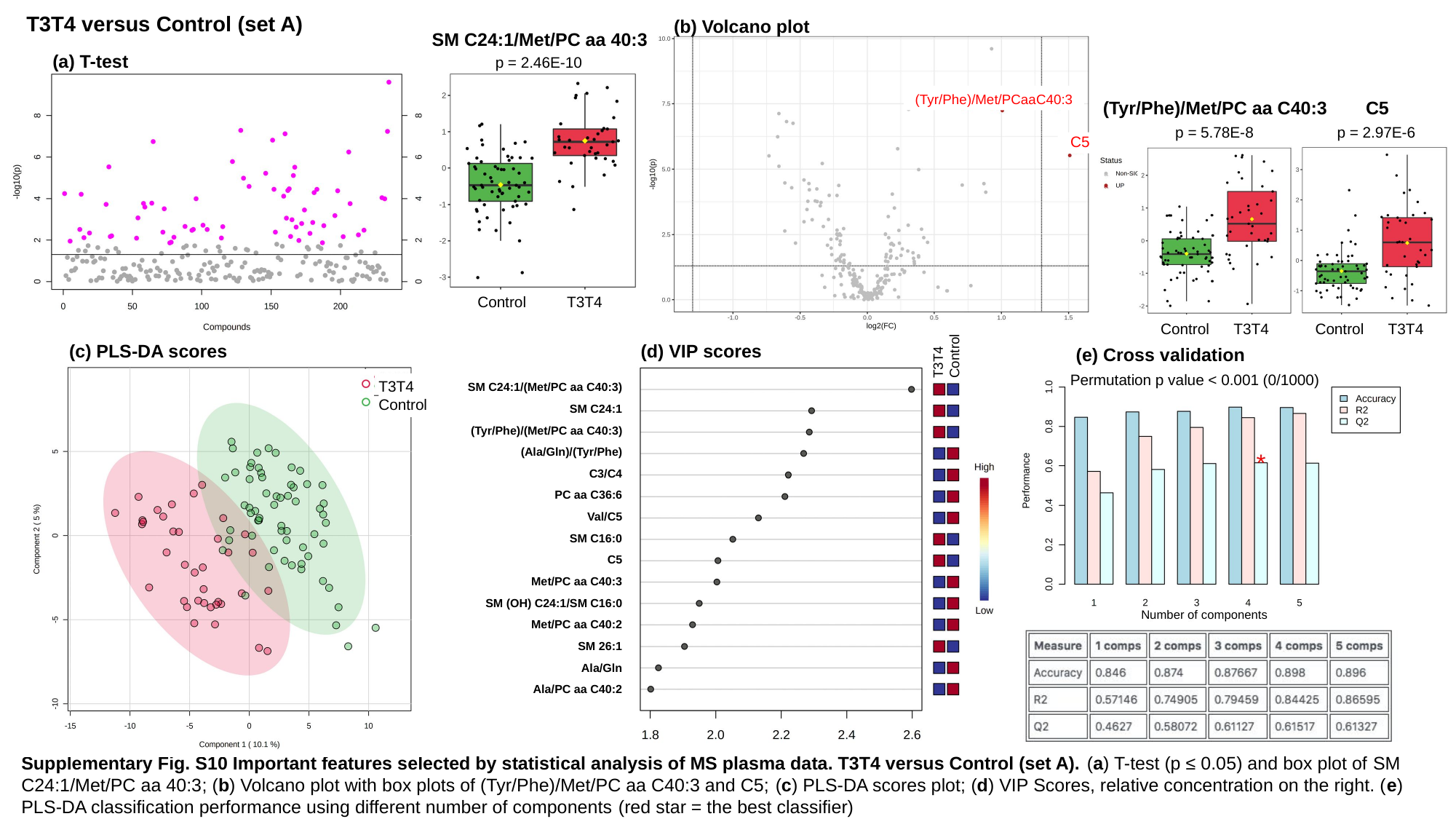

T3T4 versus Control (set A)
(b) Volcano plot
SM C24:1/Met/PC aa 40:3
Permutation p value < 0.001 (0/1000)
Number of components
Supplementary Fig. S10 Important features selected by statistical analysis of MS plasma data. T3T4 versus Control (set A). (a) T-test (p ≤ 0.05) and box plot of SM C24:1/Met/PC aa 40:3; (b) Volcano plot with box plots of (Tyr/Phe)/Met/PC aa C40:3 and C5; (c) PLS-DA scores plot; (d) VIP Scores, relative concentration on the right. (e) PLS-DA classification performance using different number of components (red star = the best classifier)
SM C24:1/(Met/PC aa C40:3)
SM C24:1
(Tyr/Phe)/(Met/PC aa C40:3)
(Ala/Gln)/(Tyr/Phe)
C3/C4
PC aa C36:6
Val/C5
SM C16:0
C5
Met/PC aa C40:3
SM (OH) C24:1/SM C16:0
Met/PC aa C40:2
SM 26:1
Ala/Gln
Ala/PC aa C40:2
C5
(a) T-test
(Tyr/Phe)/Met/PC aa C40:3
(d) VIP scores
(c) PLS-DA scores plot
(e) Cross validation
p = 2.46E-10
(Tyr/Phe)/Met/PCaaC40:3
p = 5.78E-8
p = 2.97E-6
C5
T3T4
Control
T3T4
Control
Control
T3T4
Control
Control
T3T4
T3T4

## Slide 14
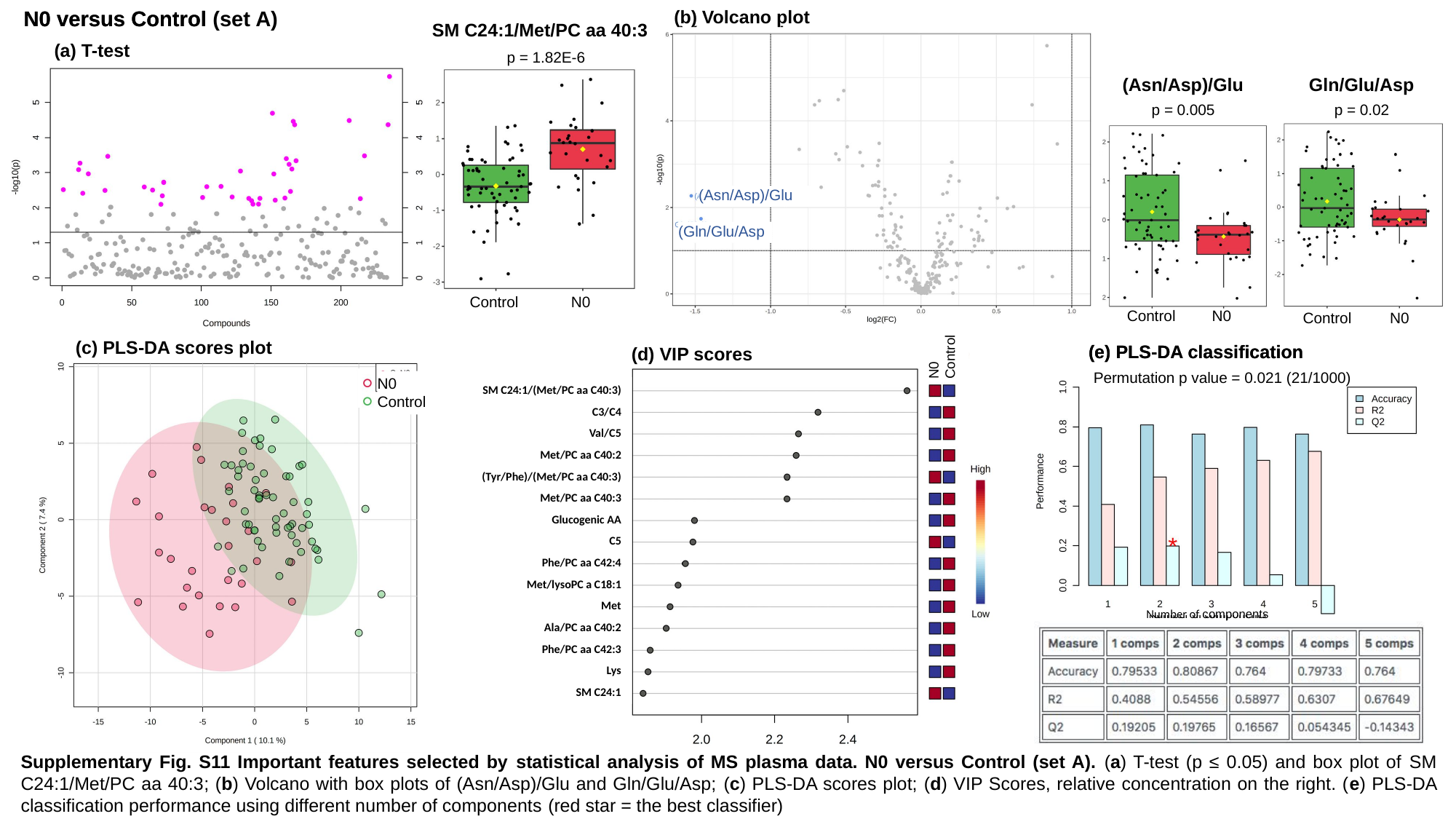

N0 versus Control
(b) Volcano plot
SM C24:1/Met/PC aa 40:3
(Asn/Asp)/Glu
Gln/Glu/Asp
SM C24:1/(Met/PC aa C40:3)
C3/C4
Val/C5
Met/PC aa C40:2
(Tyr/Phe)/(Met/PC aa C40:3)
Met/PC aa C40:3
Glucogenic AA
C5
Phe/PC aa C42:4
Met/lysoPC a C18:1
Met
Ala/PC aa C40:2
Phe/PC aa C42:3
Lys
SM C24:1
Number of components
Supplementary Fig. S11 Important features selected by statistical analysis of MS plasma data. N0 versus Control (set A). (a) T-test (p ≤ 0.05) and box plot of SM C24:1/Met/PC aa 40:3; (b) Volcano with box plots of (Asn/Asp)/Glu and Gln/Glu/Asp; (c) PLS-DA scores plot; (d) VIP Scores, relative concentration on the right. (e) PLS-DA classification performance using different number of components (red star = the best classifier)
(a) T-test
(c) PLS-DA scores plot
(e) PLS-DA classification
(d) VIP scores
p = 0.005
p = 0.02
(Asn/Asp)/Glu
(Gln/Glu/Asp
N0
Control
N0
Control
N0 versus Control (set A)
(b) Volcano plot
N0
Control
Permutation p value = 0.021 (21/1000)
SM C24:1/(Met/PC aa C40:3)
C3/C4
Val/C5
Met/PC aa C40:2
(Tyr/Phe)/(Met/PC aa C40:3)
Met/PC aa C40:3
Glucogenic AA
C5
Phe/PC aa C42:4
Met/lysoPC a C18:1
Met
Ala/PC aa C40:2
Phe/PC aa C42:3
Lys
SM C24:1
Number of components
(a) T-test
(c) PLS-DA scores plot
(e) PLS-DA classification
(d) VIP scores
(Asn/Asp)/Glu
(Gln/Glu/Asp
N0
Control
N0
Control
p = 1.82E-6
N0
Control
N0
Control

## Slide 15
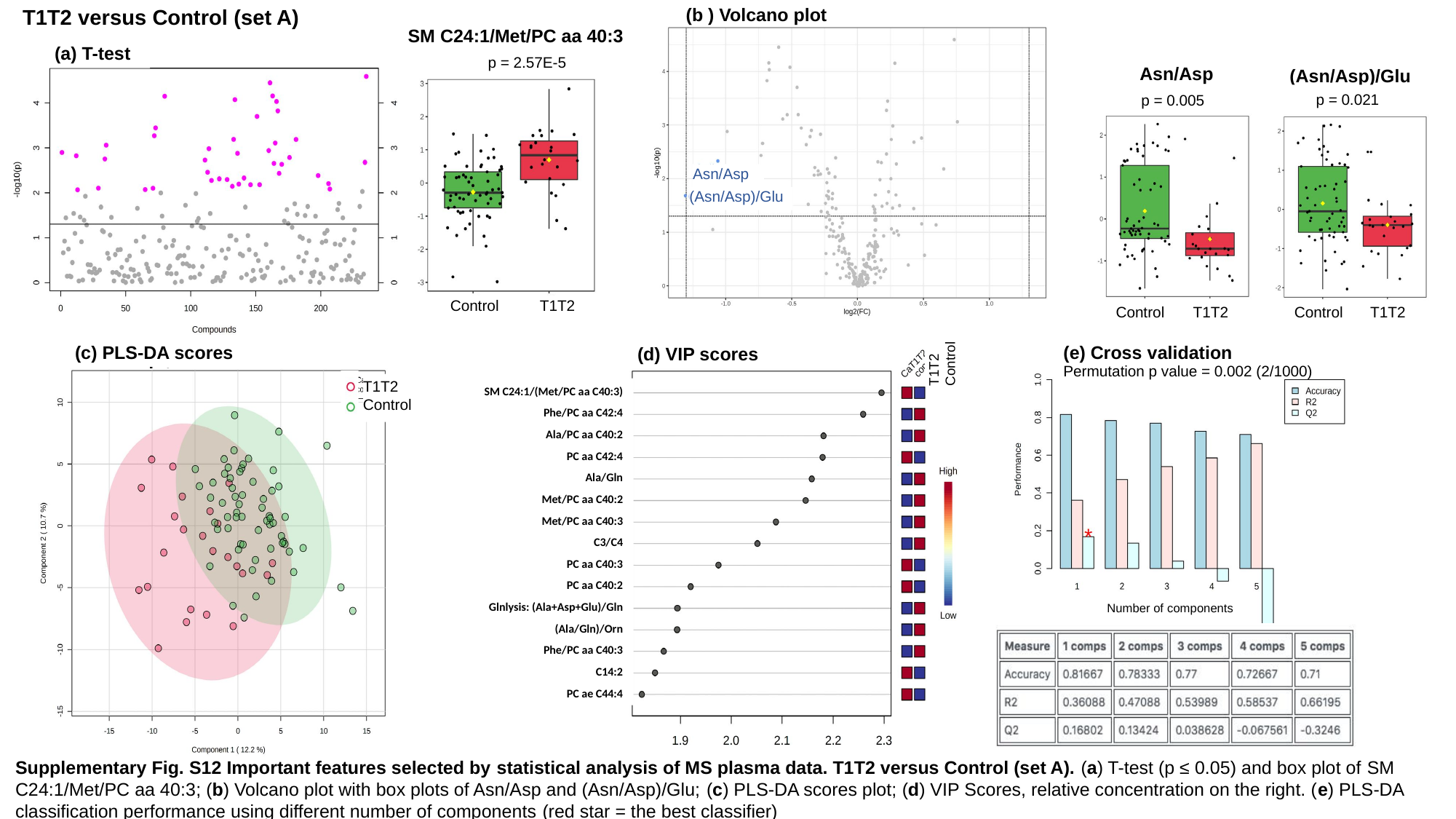

(b ) Volcano plot
T1T2 versus Control (set A)
SM C24:1/Met/PC aa 40:3
(a) T-test
p = 2.57E-5
Asn/Asp
(Asn/Asp)/Glu
p = 0.021
p = 0.005
Asn/Asp
(Asn/Asp)/Glu
Control
T1T2
Control
T1T2
Control
T1T2
(c) PLS-DA scores plot
(e) Cross validation
T1T2
Control
(d) VIP scores
Permutation p value = 0.002 (2/1000)
T1T2
Control
SM C24:1/(Met/PC aa C40:3)
Phe/PC aa C42:4
Ala/PC aa C40:2
PC aa C42:4
Ala/Gln
Met/PC aa C40:2
Met/PC aa C40:3
C3/C4
PC aa C40:3
PC aa C40:2
Glnlysis: (Ala+Asp+Glu)/Gln
(Ala/Gln)/Orn
Phe/PC aa C40:3
C14:2
PC ae C44:4
Number of components
Supplementary Fig. S12 Important features selected by statistical analysis of MS plasma data. T1T2 versus Control (set A). (a) T-test (p ≤ 0.05) and box plot of SM C24:1/Met/PC aa 40:3; (b) Volcano plot with box plots of Asn/Asp and (Asn/Asp)/Glu; (c) PLS-DA scores plot; (d) VIP Scores, relative concentration on the right. (e) PLS-DA classification performance using different number of components (red star = the best classifier)

## Slide 16
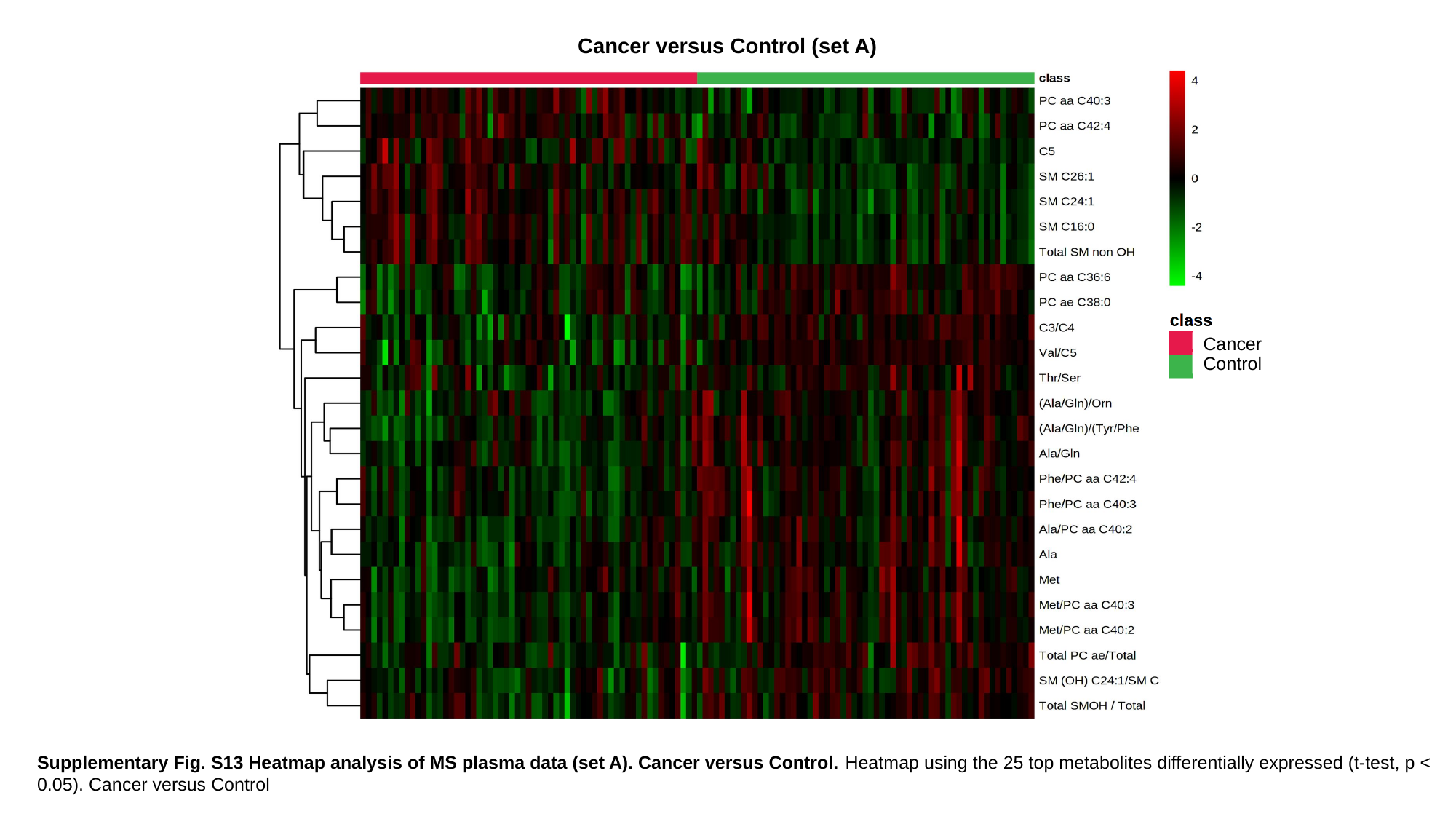

Cancer versus Control (set A)
Cancer
Control
Supplementary Fig. S13 Heatmap analysis of MS plasma data (set A). Cancer versus Control. Heatmap using the 25 top metabolites differentially expressed (t-test, p < 0.05). Cancer versus Control

## Slide 17
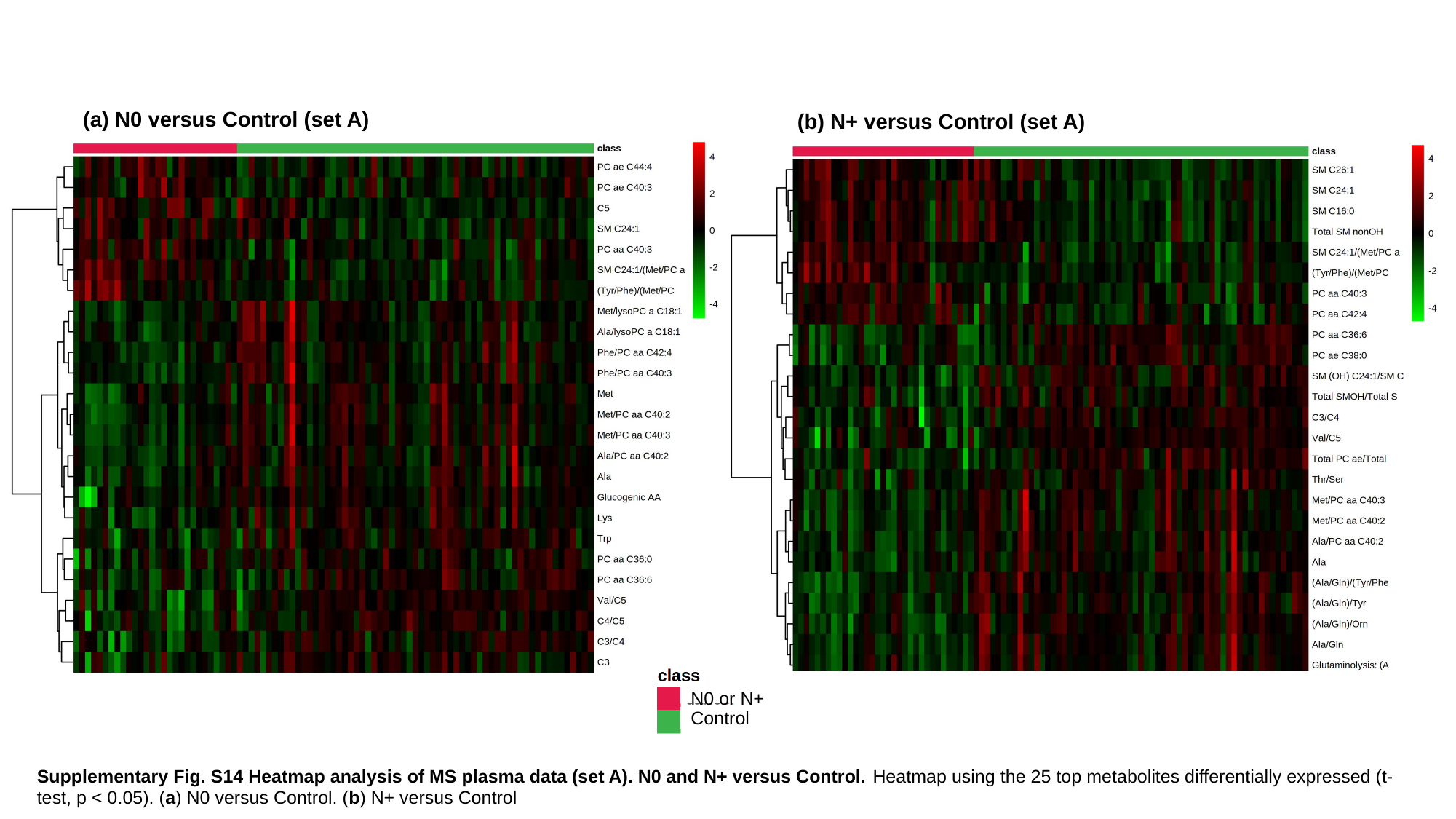

(a) N0 versus Control (set A)
(b) N+ versus Control (set A)
N0 or N+
Control
Supplementary Fig. S14 Heatmap analysis of MS plasma data (set A). N0 and N+ versus Control. Heatmap using the 25 top metabolites differentially expressed (t-test, p < 0.05). (a) N0 versus Control. (b) N+ versus Control

## Slide 18
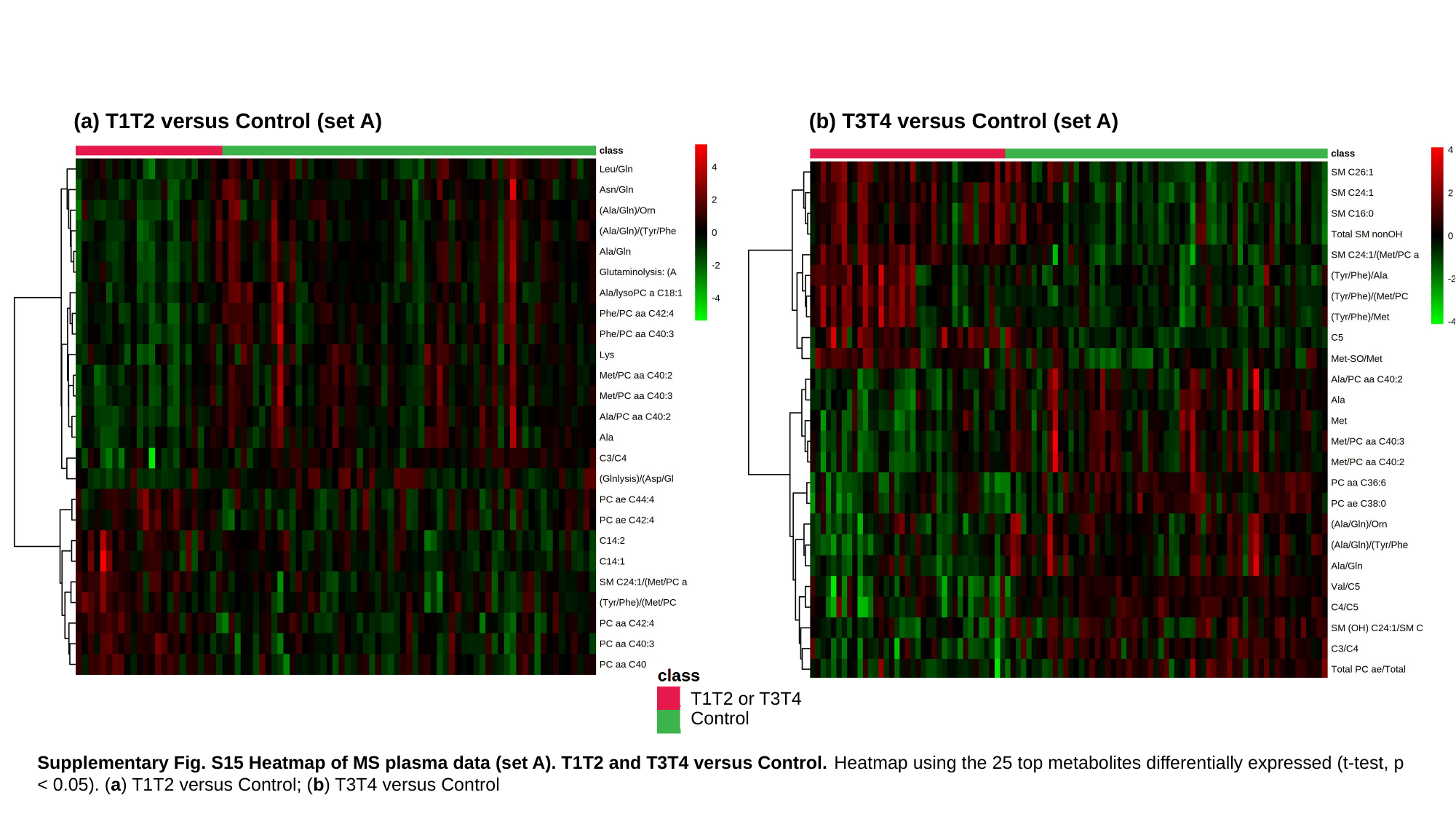

(a) T1T2 versus Control (set A)
(b) T3T4 versus Control (set A)
T1T2 or T3T4
Control
Supplementary Fig. S15 Heatmap of MS plasma data (set A). T1T2 and T3T4 versus Control. Heatmap using the 25 top metabolites differentially expressed (t-test, p < 0.05). (a) T1T2 versus Control; (b) T3T4 versus Control

## Slide 19
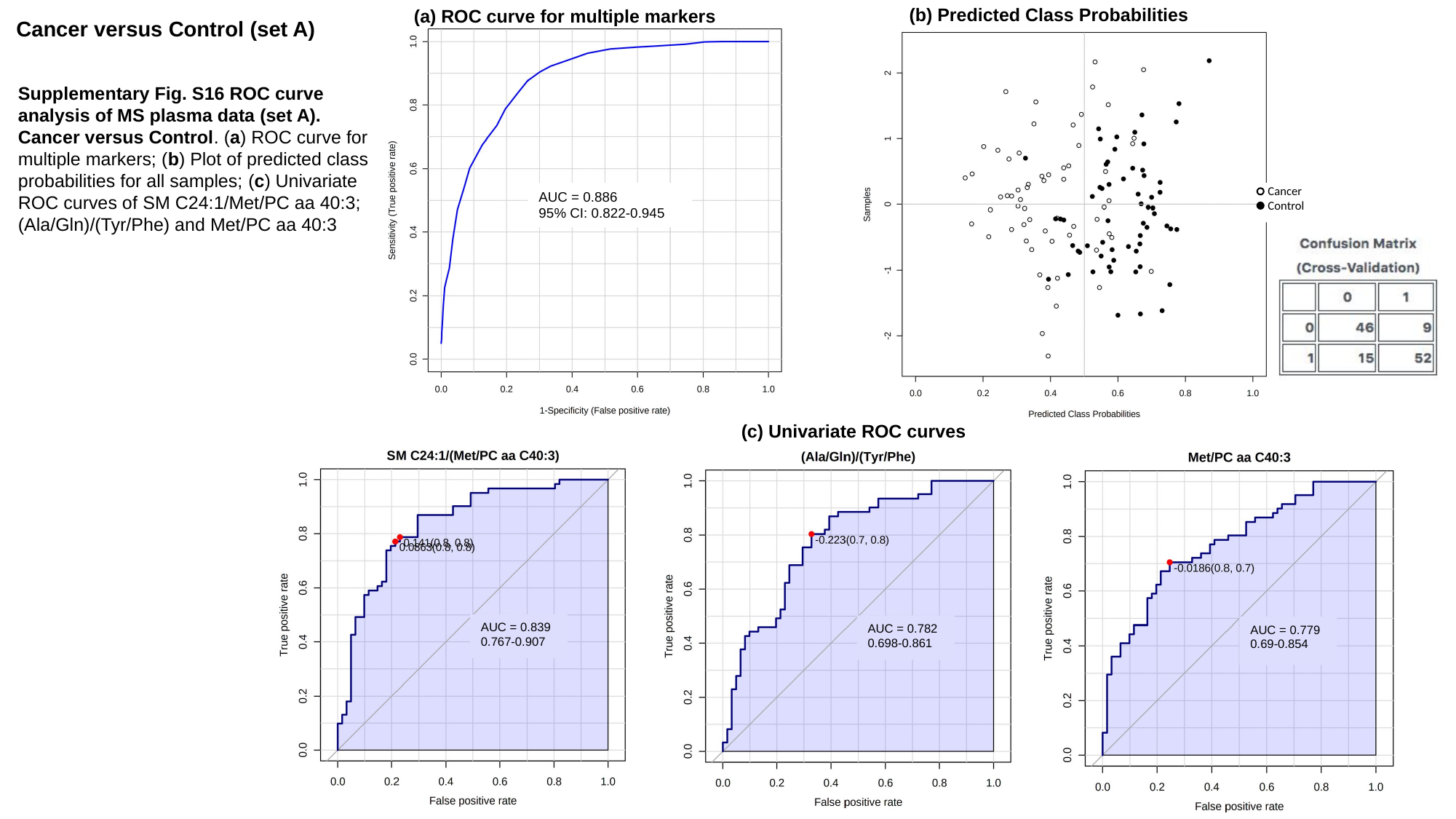

(b) Predicted Class Probabilities
(a) ROC curve for multiple markers
Cancer versus Control (set A)
Supplementary Fig. S16 ROC curve analysis of MS plasma data (set A). Cancer versus Control. (a) ROC curve for multiple markers; (b) Plot of predicted class probabilities for all samples; (c) Univariate ROC curves of SM C24:1/Met/PC aa 40:3; (Ala/Gln)/(Tyr/Phe) and Met/PC aa 40:3
AUC = 0.886
95% CI: 0.822-0.945
(c) Univariate ROC curves
AUC = 0.839
0.767-0.907
AUC = 0.782
0.698-0.861
AUC = 0.779
0.69-0.854
Cancer
Control

## Slide 20
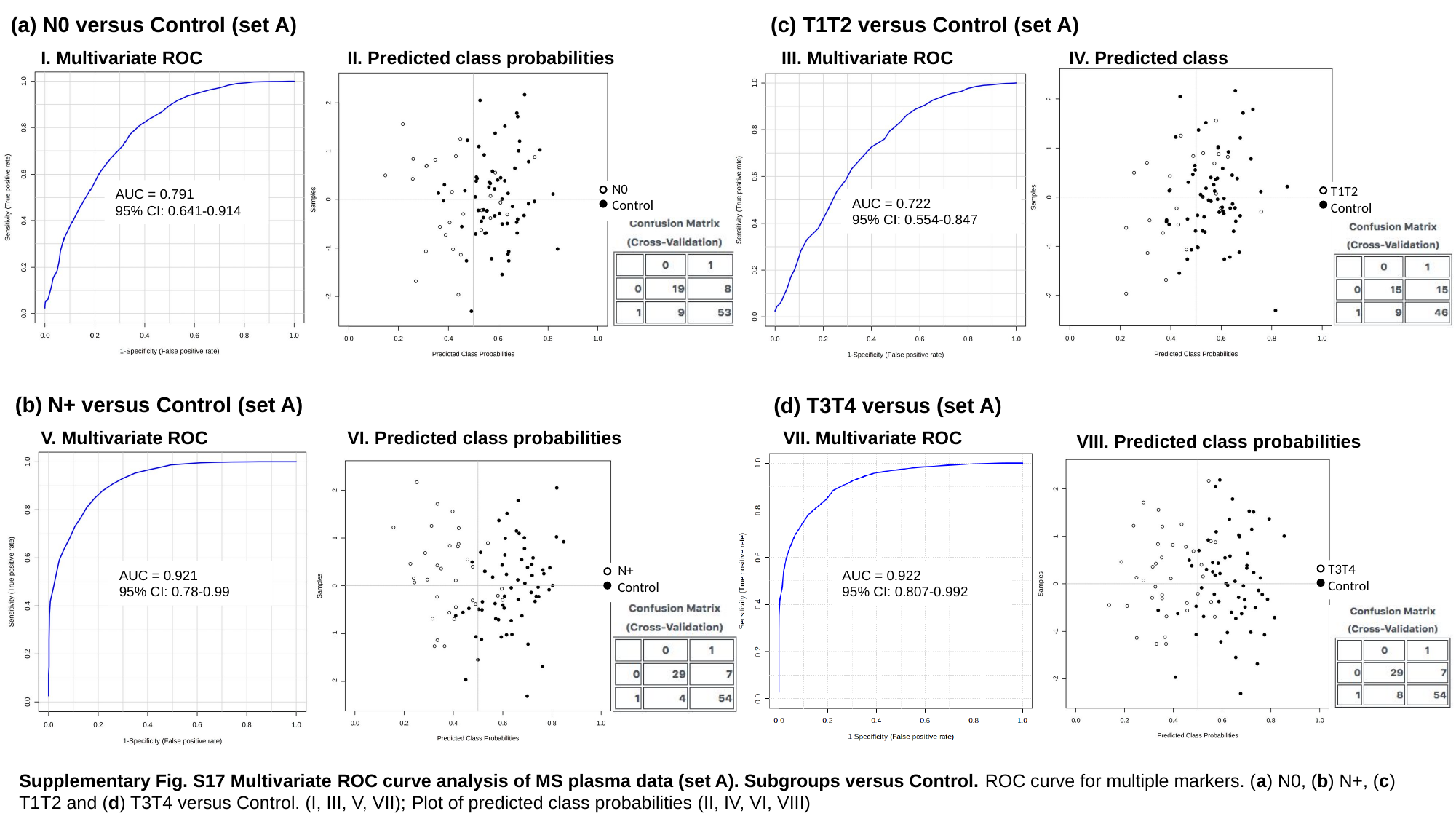

(a) N0 versus Control (set A)
(c) T1T2 versus Control (set A)
I. Multivariate ROC curve
II. Predicted class probabilities
III. Multivariate ROC curve
IV. Predicted class probabilities
(b) N+ versus Control (set A)
(d) T3T4 versus (set A) Control
VI. Predicted class probabilities
V. Multivariate ROC curve
VII. Multivariate ROC curve
VIII. Predicted class probabilities
AUC = 0.791
95% CI: 0.641-0.914
AUC = 0.722
95% CI: 0.554-0.847
AUC = 0.921
95% CI: 0.78-0.99
AUC = 0.922
95% CI: 0.807-0.992
Supplementary Fig. S17 Multivariate ROC curve analysis of MS plasma data (set A). Subgroups versus Control. ROC curve for multiple markers. (a) N0, (b) N+, (c) T1T2 and (d) T3T4 versus Control. (I, III, V, VII); Plot of predicted class probabilities (II, IV, VI, VIII)
N0
Control
T1T2
Control
T3T4
Control
N+
Control

## Slide 21
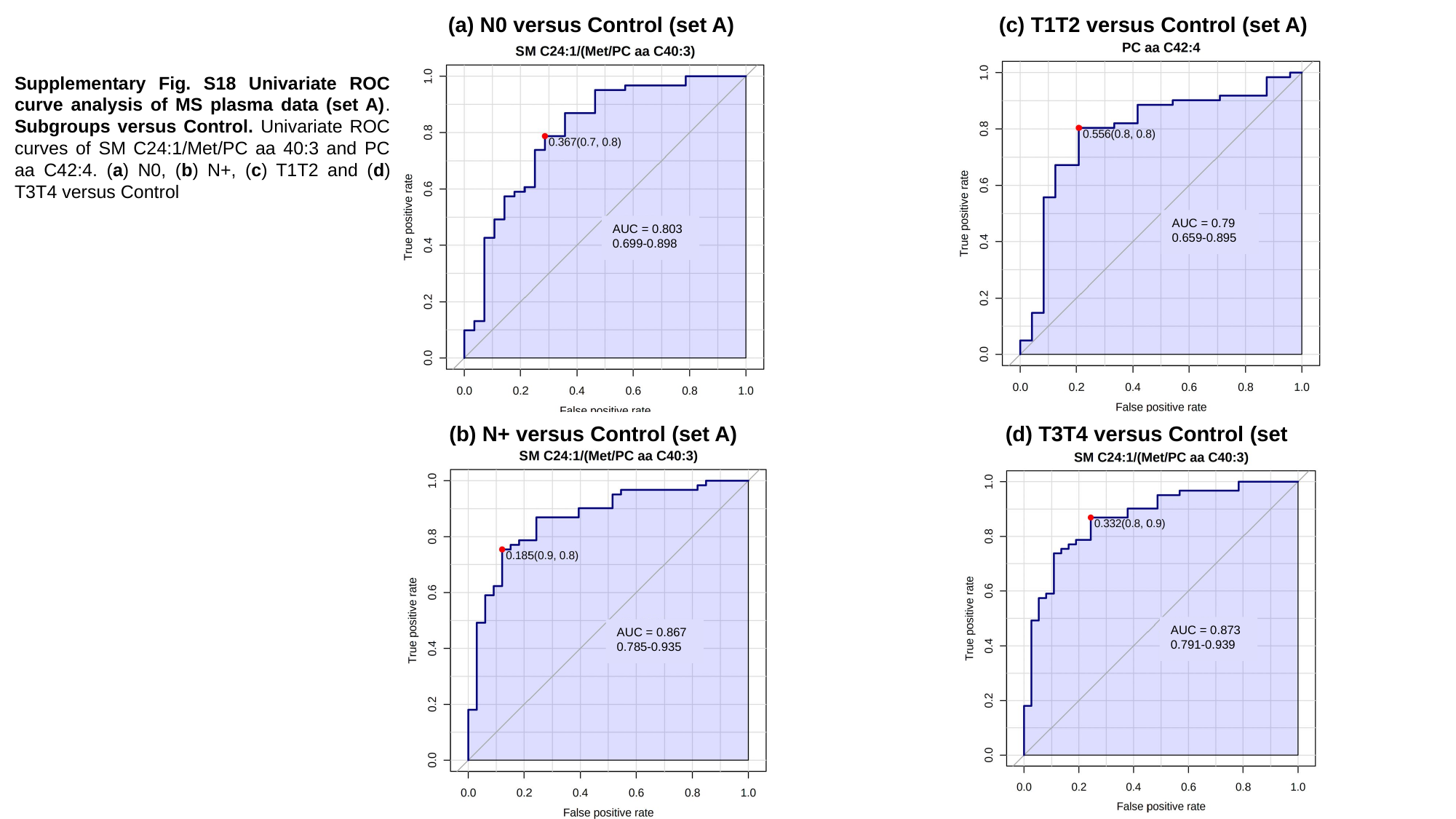

(a) N0 versus Control (set A)
(c) T1T2 versus Control (set A)
(b) N+ versus Control (set A)
(d) T3T4 versus Control (set A)
Supplementary Fig. S18 Univariate ROC curve analysis of MS plasma data (set A). Subgroups versus Control. Univariate ROC curves of SM C24:1/Met/PC aa 40:3 and PC aa C42:4. (a) N0, (b) N+, (c) T1T2 and (d) T3T4 versus Control
AUC = 0.79
0.659-0.895
AUC = 0.803
0.699-0.898
AUC = 0.873
0.791-0.939
AUC = 0.867
0.785-0.935

## Slide 22
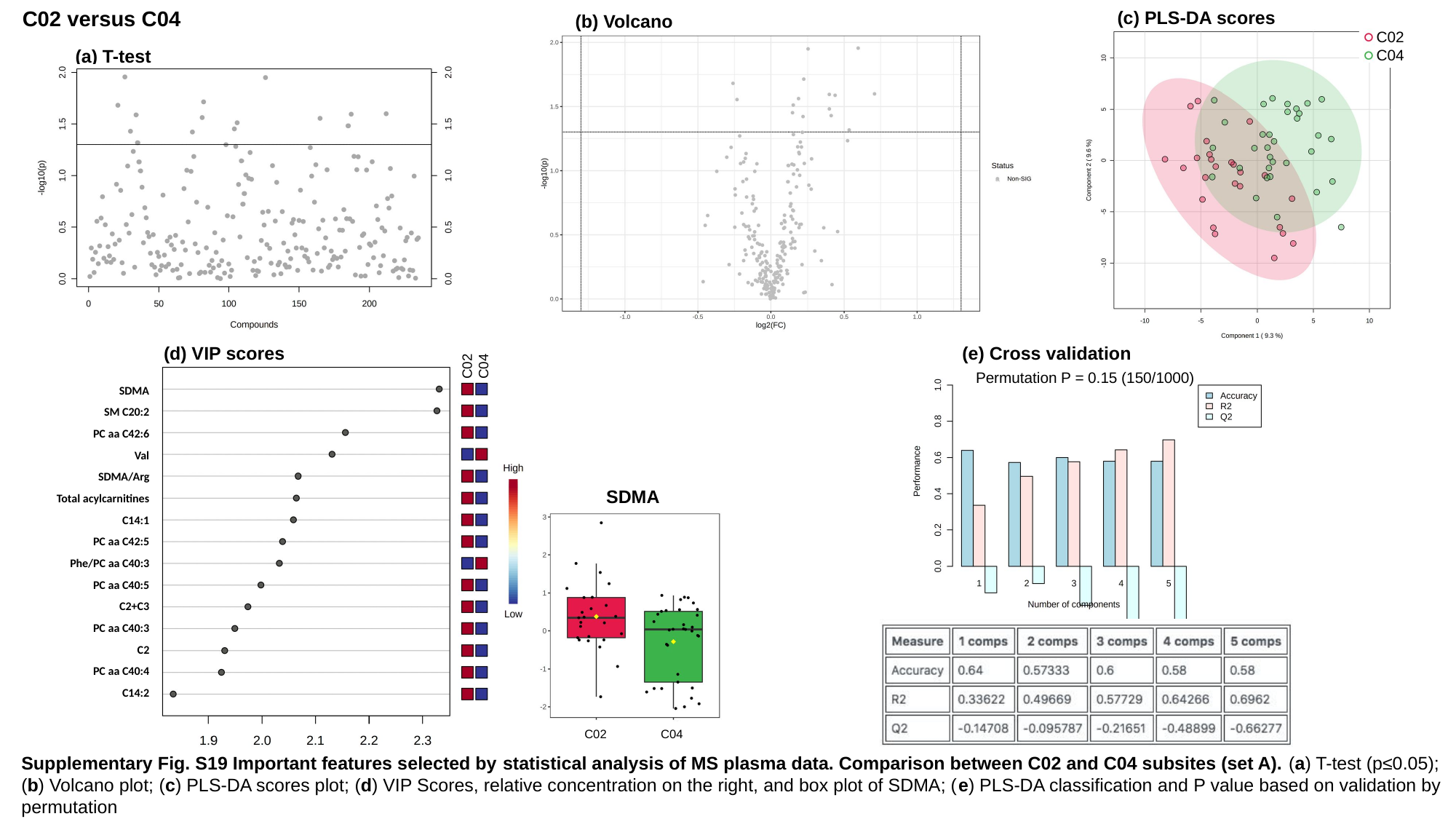

C02 versus C04
(c) PLS-DA scores plot
(b) Volcano plot
C02
C04
(a) T-test
C02
C04
(d) VIP scores
(e) Cross validation
Permutation P = 0.15 (150/1000)
SDMA
SM C20:2
PC aa C42:6
Val
SDMA/Arg
Total acylcarnitines
C14:1
PC aa C42:5
Phe/PC aa C40:3
PC aa C40:5
C2+C3
PC aa C40:3
C2
PC aa C40:4
C14:2
SDMA
C02
C04
Supplementary Fig. S19 Important features selected by statistical analysis of MS plasma data. Comparison between C02 and C04 subsites (set A). (a) T-test (p≤0.05); (b) Volcano plot; (c) PLS-DA scores plot; (d) VIP Scores, relative concentration on the right, and box plot of SDMA; (e) PLS-DA classification and P value based on validation by permutation

## Slide 23
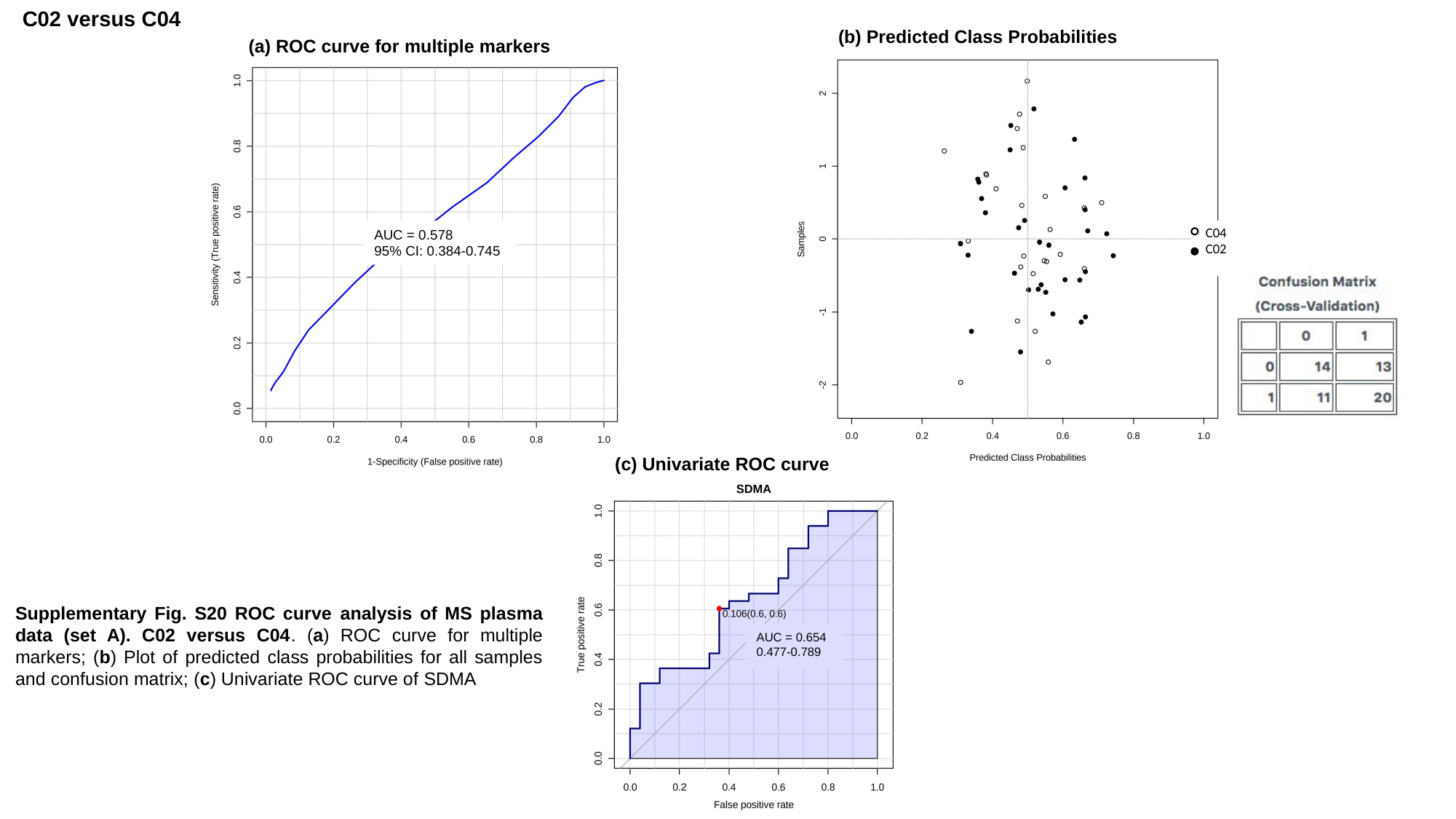

C02 versus C04
(b) Predicted Class Probabilities
(a) ROC curve for multiple markers
C04
C02
AUC = 0.578
95% CI: 0.384-0.745
(c) Univariate ROC curve
Supplementary Fig. S20 ROC curve analysis of MS plasma data (set A). C02 versus C04. (a) ROC curve for multiple markers; (b) Plot of predicted class probabilities for all samples and confusion matrix; (c) Univariate ROC curve of SDMA
AUC = 0.654
0.477-0.789

## Slide 24
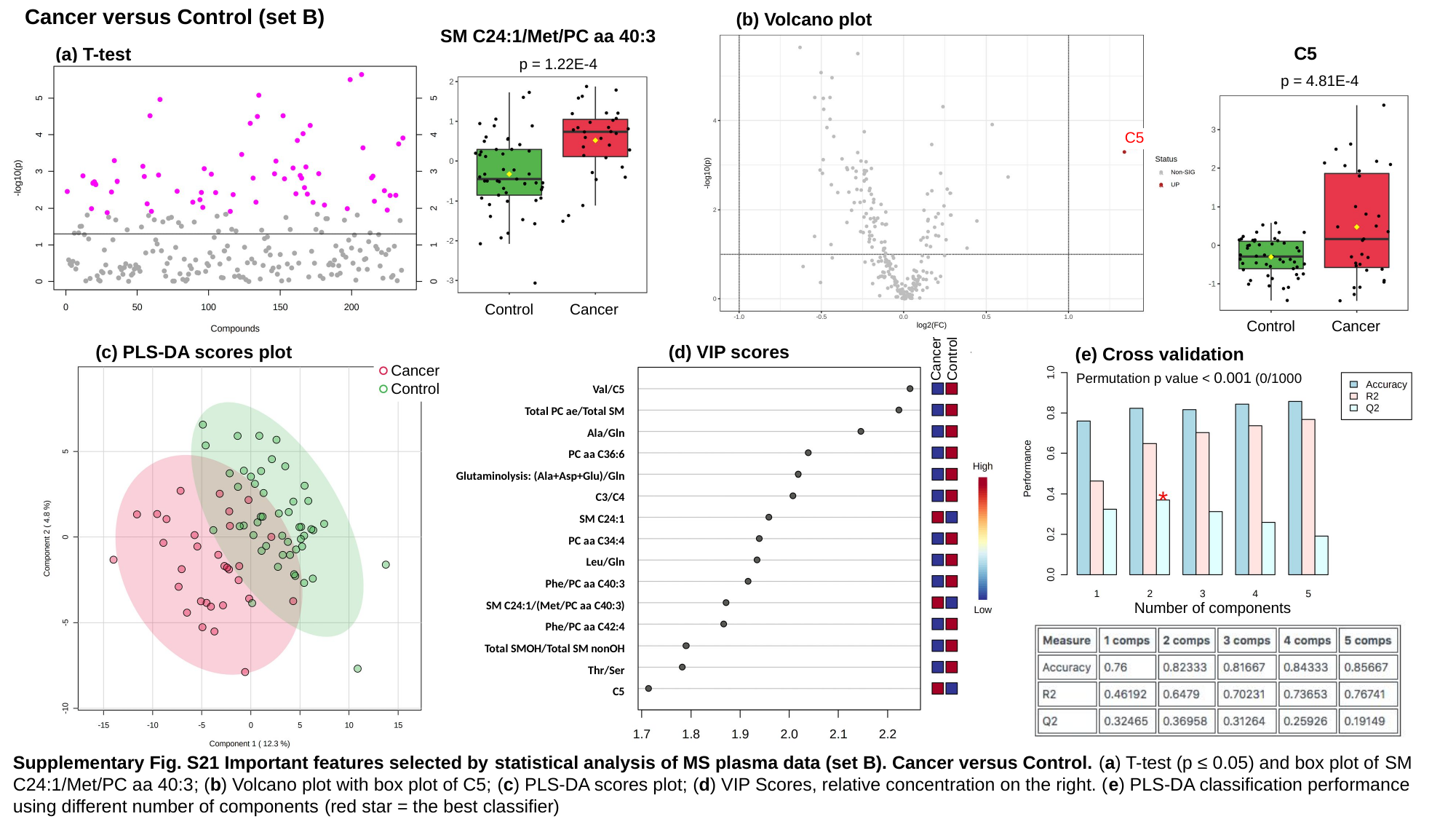

Cancer versus Control (set B)
(b) Volcano plot
SM C24:1/Met/PC aa 40:3
C5
(a) T-test
(e) Cross validation
Number of components
Supplementary Fig. S21 Important features selected by statistical analysis of MS plasma data (set B). Cancer versus Control. (a) T-test (p ≤ 0.05) and box plot of SM C24:1/Met/PC aa 40:3; (b) Volcano plot with box plot of C5; (c) PLS-DA scores plot; (d) VIP Scores, relative concentration on the right. (e) PLS-DA classification performance using different number of components (red star = the best classifier)
p = 1.22E-4
p = 4.81E-4
C5
Control
Cancer
Cancer
Control
(c) PLS-DA scores plot
(d) VIP scores
Cancer
Control
Permutation p value < 0.001 (0/1000
Val/C5
Total PC ae/Total SM
Ala/Gln
PC aa C36:6
Glutaminolysis: (Ala+Asp+Glu)/Gln
C3/C4
SM C24:1
PC aa C34:4
Leu/Gln
Phe/PC aa C40:3
SM C24:1/(Met/PC aa C40:3)
Phe/PC aa C42:4
Total SMOH/Total SM nonOH
Thr/Ser
C5
Control
Cancer

## Slide 25
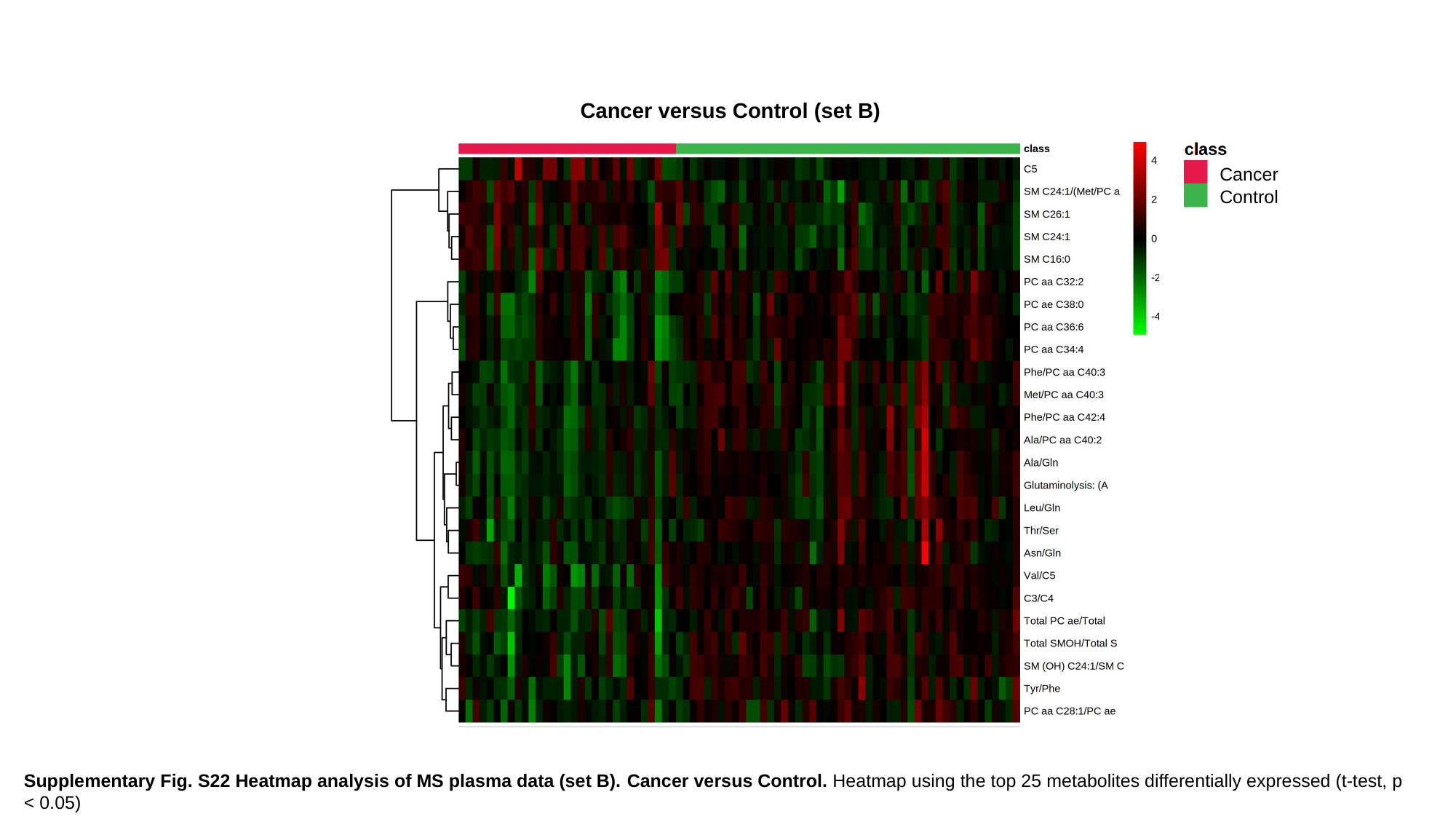

Cancer versus Control (set B)
Cancer
Control
Supplementary Fig. S22 Heatmap analysis of MS plasma data (set B). Cancer versus Control. Heatmap using the top 25 metabolites differentially expressed (t-test, p < 0.05)

## Slide 26
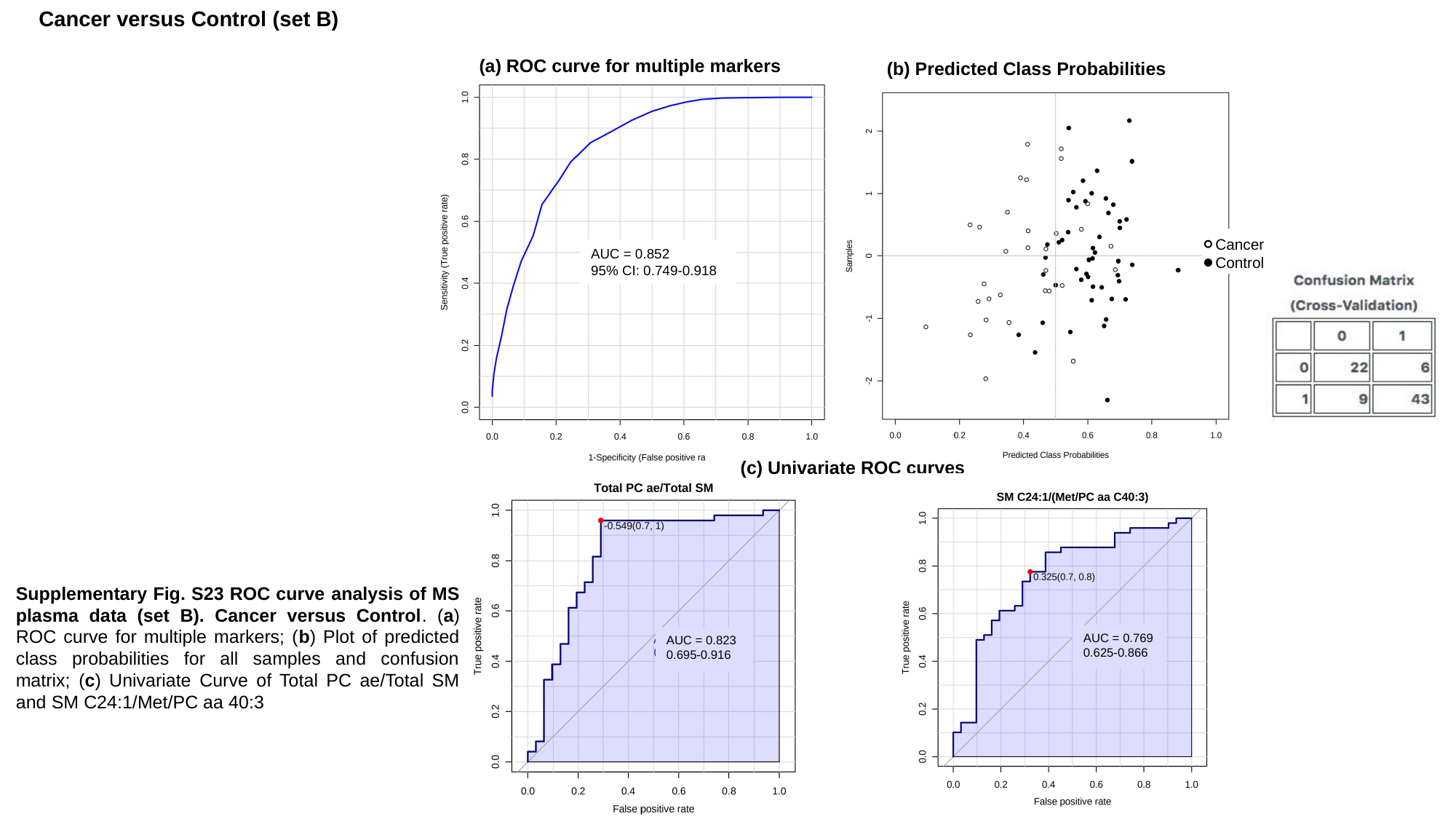

Cancer versus Control (set B)
(a) ROC curve for multiple markers
(b) Predicted Class Probabilities
AUC = 0.852
95% CI: 0.749-0.918
(c) Univariate ROC curves
Supplementary Fig. S23 ROC curve analysis of MS plasma data (set B). Cancer versus Control. (a) ROC curve for multiple markers; (b) Plot of predicted class probabilities for all samples and confusion matrix; (c) Univariate Curve of Total PC ae/Total SM and SM C24:1/Met/PC aa 40:3
AUC = 0.823
0.695-0.916
Cancer
Control
AUC = 0.769
0.625-0.866

## Slide 27
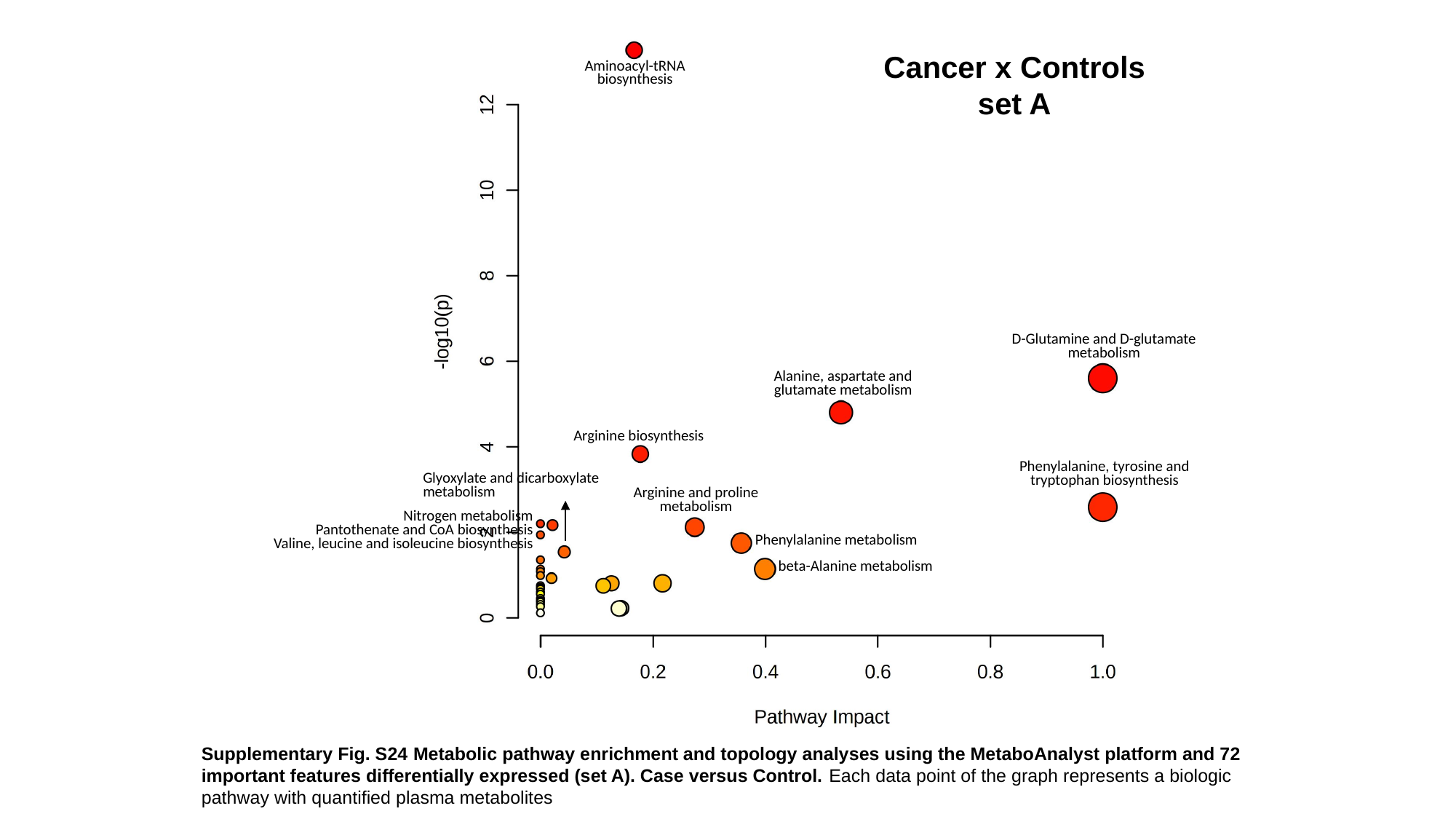

Aminoacyl-tRNA biosynthesis
D-Glutamine and D-glutamate metabolism
Alanine, aspartate and glutamate metabolism
Arginine biosynthesis
Phenylalanine, tyrosine and tryptophan biosynthesis
Glyoxylate and dicarboxylate metabolism
Arginine and proline metabolism
Nitrogen metabolism
Pantothenate and CoA biosynthesis
Valine, leucine and isoleucine biosynthesis
Phenylalanine metabolism
beta-Alanine metabolism
Cancer x Controls
set A
Supplementary Fig. S24 Metabolic pathway enrichment and topology analyses using the MetaboAnalyst platform and 72 important features differentially expressed (set A). Case versus Control. Each data point of the graph represents a biologic pathway with quantified plasma metabolites

## Slide 28
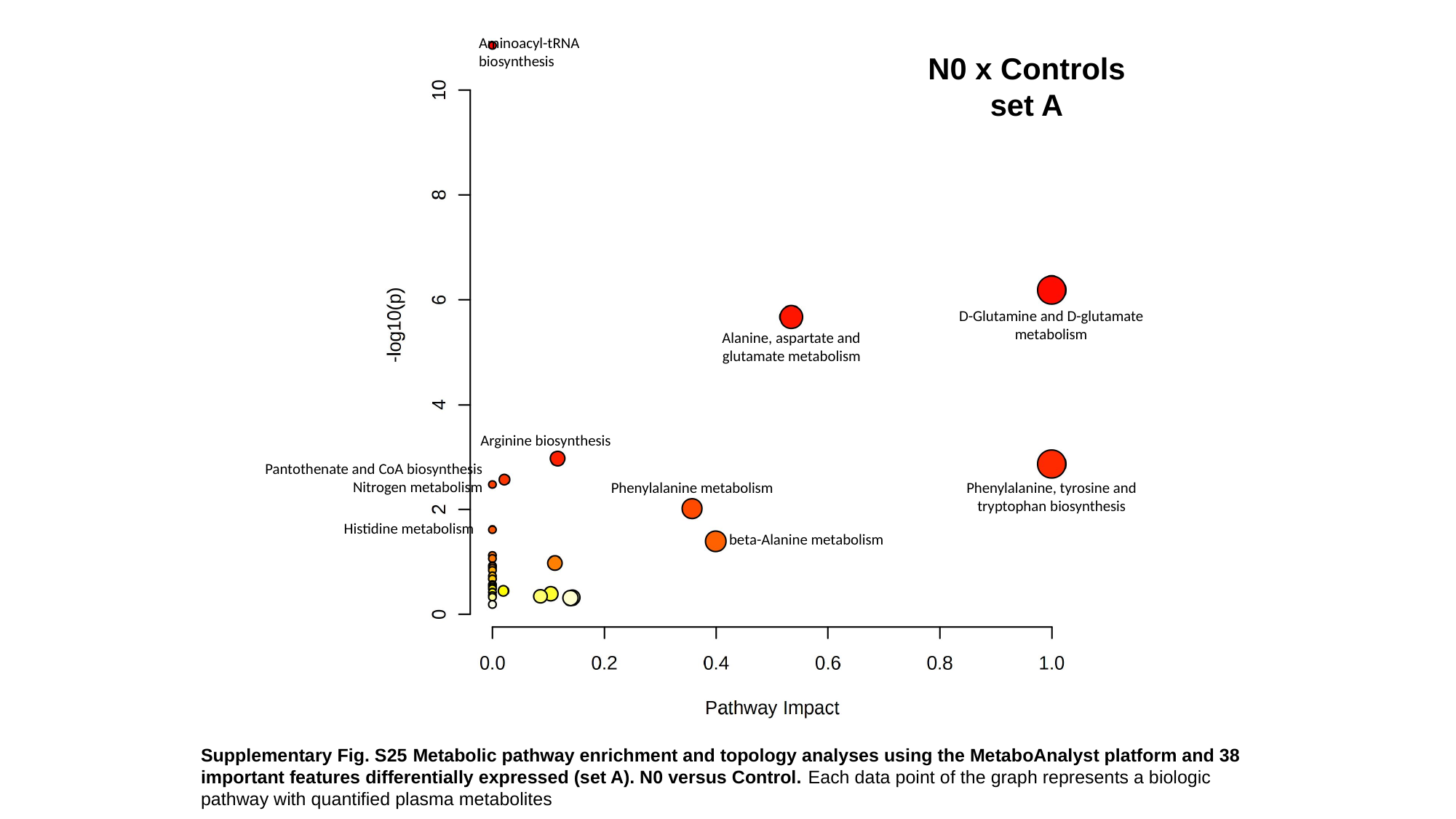

Aminoacyl-tRNA biosynthesis
D-Glutamine and D-glutamate metabolism
Alanine, aspartate and glutamate metabolism
Phenylalanine, tyrosine and tryptophan biosynthesis
Phenylalanine metabolism
Histidine metabolism
beta-Alanine metabolism
Arginine biosynthesis
Pantothenate and CoA biosynthesis
Nitrogen metabolism
Supplementary Fig. S25 Metabolic pathway enrichment and topology analyses using the MetaboAnalyst platform and 38 important features differentially expressed (set A). N0 versus Control. Each data point of the graph represents a biologic pathway with quantified plasma metabolites
N0 x Controls
set A

## Slide 29
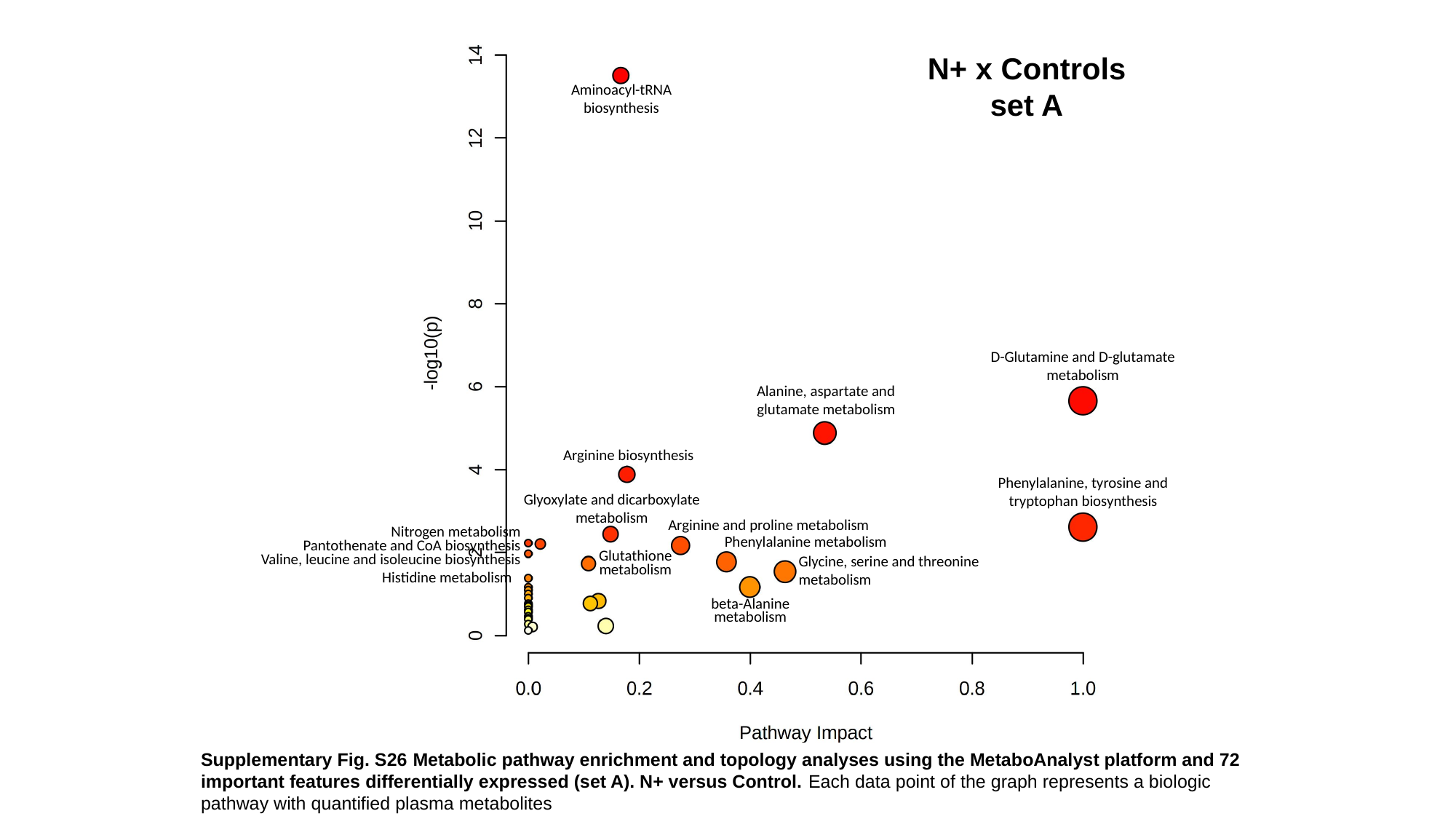

N+ x Controls
set A
Aminoacyl-tRNA biosynthesis
D-Glutamine and D-glutamate metabolism
Alanine, aspartate and glutamate metabolism
Arginine biosynthesis
Phenylalanine, tyrosine and tryptophan biosynthesis
Glyoxylate and dicarboxylate metabolism
Arginine and proline metabolism
Nitrogen metabolism
Pantothenate and CoA biosynthesis
Valine, leucine and isoleucine biosynthesis
Phenylalanine metabolism
Glutathione metabolism
Glycine, serine and threonine metabolism
Histidine metabolism
beta-Alanine metabolism
Supplementary Fig. S26 Metabolic pathway enrichment and topology analyses using the MetaboAnalyst platform and 72 important features differentially expressed (set A). N+ versus Control. Each data point of the graph represents a biologic pathway with quantified plasma metabolites

## Slide 30
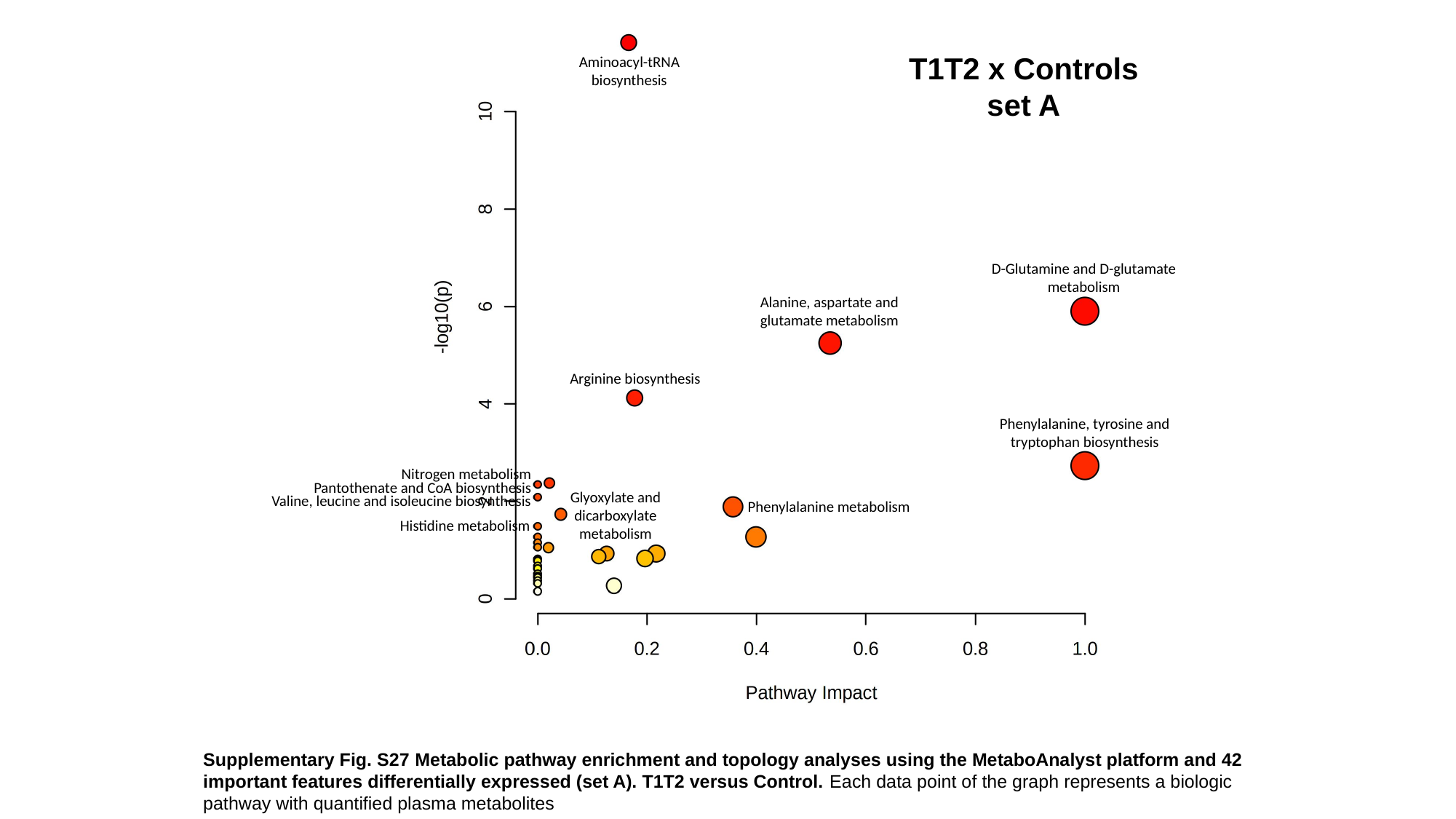

Arginine biosynthesis
T1T2 x Controls
set A
Aminoacyl-tRNA biosynthesis
D-Glutamine and D-glutamate metabolism
Alanine, aspartate and glutamate metabolism
Phenylalanine, tyrosine and tryptophan biosynthesis
Nitrogen metabolism
Pantothenate and CoA biosynthesis
Valine, leucine and isoleucine biosynthesis
Glyoxylate and dicarboxylate metabolism
Phenylalanine metabolism
Histidine metabolism
Supplementary Fig. S27 Metabolic pathway enrichment and topology analyses using the MetaboAnalyst platform and 42 important features differentially expressed (set A). T1T2 versus Control. Each data point of the graph represents a biologic pathway with quantified plasma metabolites

## Slide 31
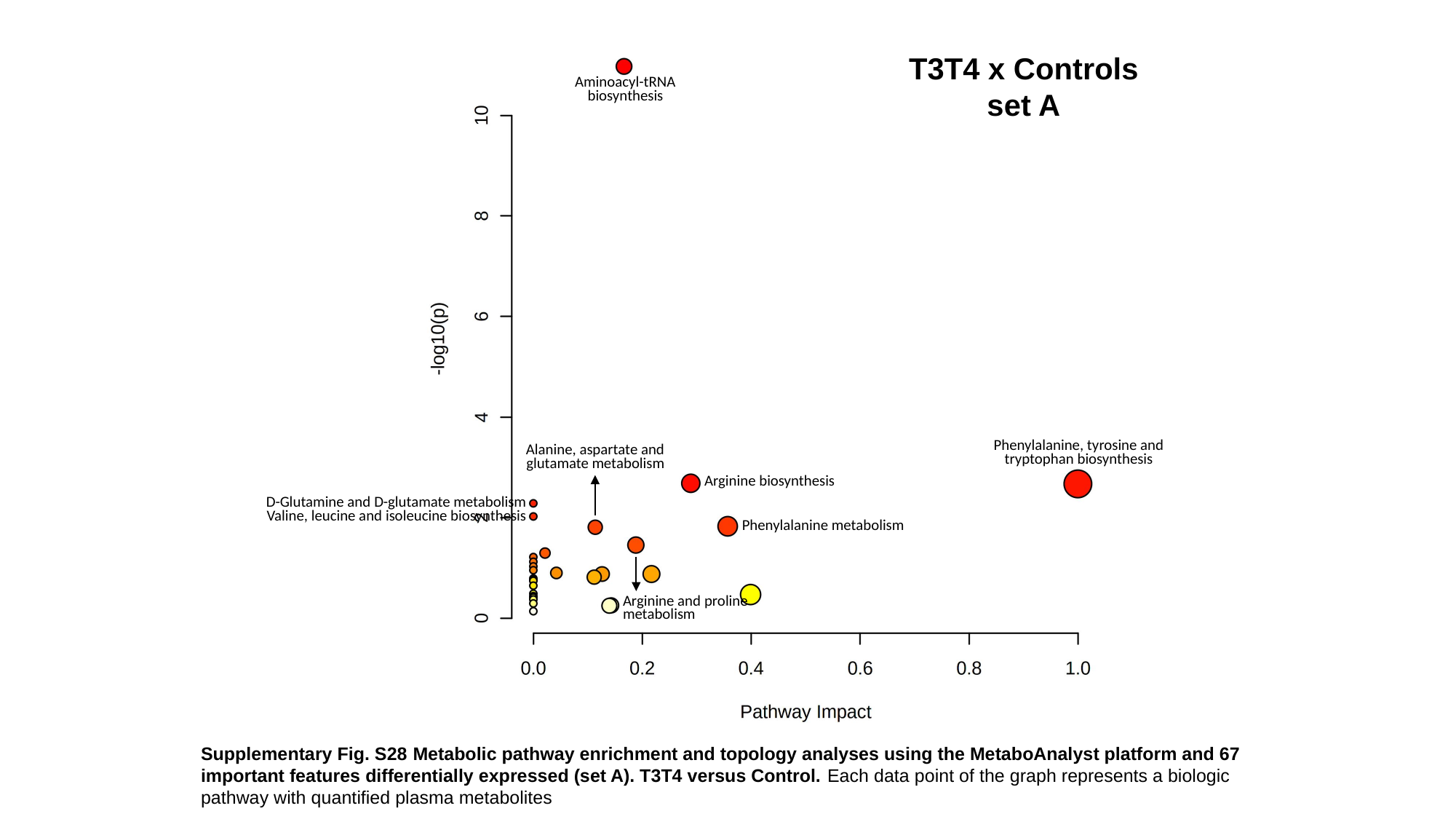

T3T4 x Controls
set A
Aminoacyl-tRNA biosynthesis
Phenylalanine, tyrosine and tryptophan biosynthesis
Alanine, aspartate and glutamate metabolism
Arginine biosynthesis
D-Glutamine and D-glutamate metabolism
Valine, leucine and isoleucine biosynthesis
Phenylalanine metabolism
Arginine and proline metabolism
Supplementary Fig. S28 Metabolic pathway enrichment and topology analyses using the MetaboAnalyst platform and 67 important features differentially expressed (set A). T3T4 versus Control. Each data point of the graph represents a biologic pathway with quantified plasma metabolites

## Slide 32
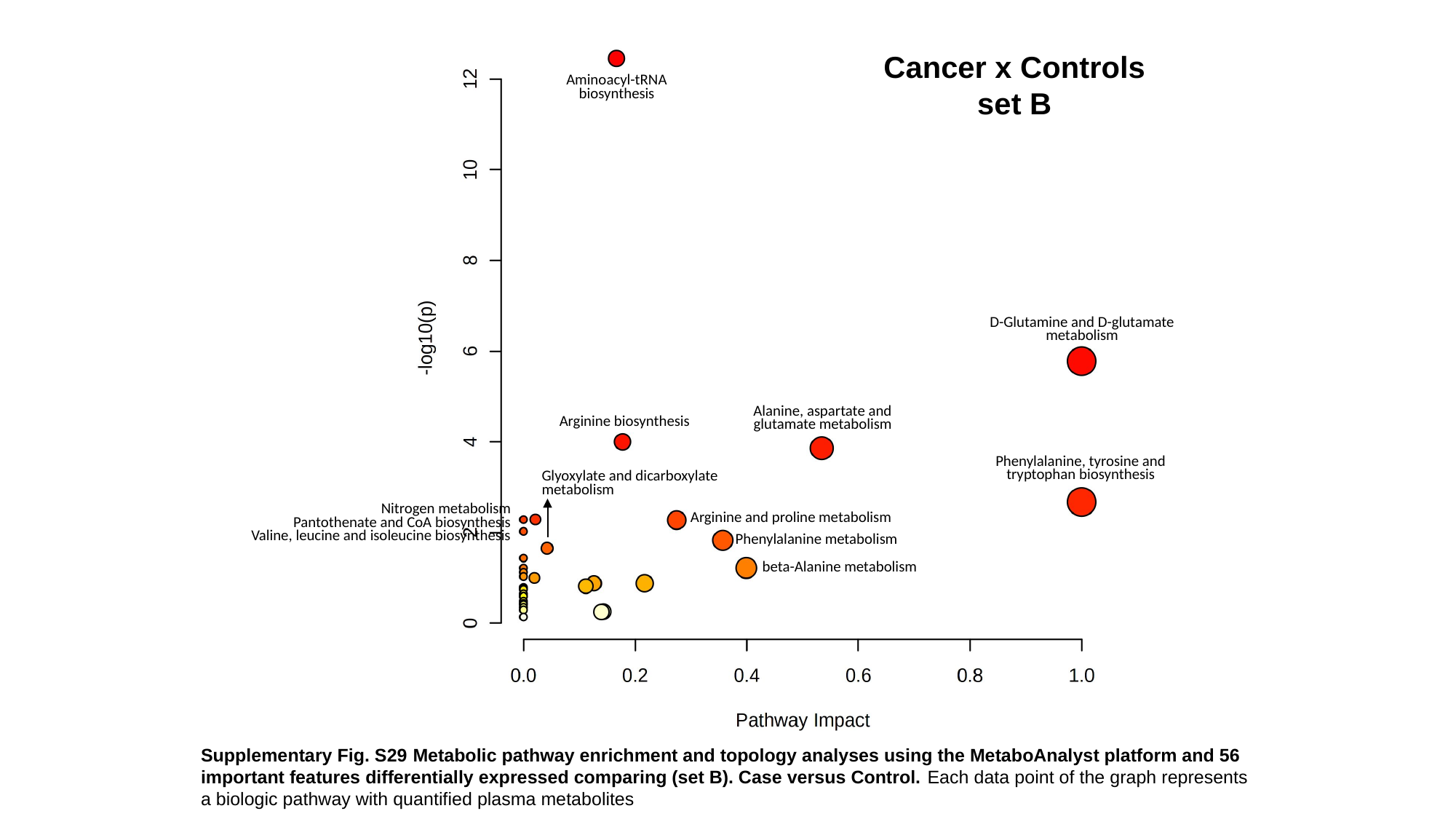

Cancer x Controls
set B
Aminoacyl-tRNA biosynthesis
D-Glutamine and D-glutamate metabolism
Alanine, aspartate and glutamate metabolism
Arginine biosynthesis
Phenylalanine, tyrosine and tryptophan biosynthesis
Glyoxylate and dicarboxylate metabolism
Arginine and proline metabolism
Phenylalanine metabolism
beta-Alanine metabolism
Nitrogen metabolism
Pantothenate and CoA biosynthesis
Valine, leucine and isoleucine biosynthesis
Supplementary Fig. S29 Metabolic pathway enrichment and topology analyses using the MetaboAnalyst platform and 56 important features differentially expressed comparing (set B). Case versus Control. Each data point of the graph represents a biologic pathway with quantified plasma metabolites
